# Supplementary material for: Risk prediction tools for pressure injury occurrence: an umbrella review of systematic reviews reporting model development and validation methods
Source: Diagn Progn Res. 2025 Jan 14;9:2. doi: 10.1186/s41512-024-00182-4 (PMC11730812; doi:10.1186/s41512-024-00182-4)
Supplement: Supplementary file 1 — Supplementary Material 1: Appendix 1. PRISMA 2020 Checklist. Appendix 2. Description of search strategies. Appendix 3. Data extraction form. Appendix 4. AMSTAR-2 Methodology Quality Appraisal. Adapted for application to reviews of prognostic model and accuracy studies. Appendix 5. Detailed results tables. Table S1. Full-text articles excluded, with reasons. Table S2. Systematic review characteristics. Table S3. AMSTAR-2 assessment results per review. Table S4. Risk prediction tool characteristics, ascertained at review level. Table S5. Prognostic model external validation study characteristics. Table S6. Table of Predictors, by tool (predictors were reported for 66 tools), ascertained at review level except in the case of discrepancies between reviews. [file 41512_2024_182_MOESM1_ESM.docx]

Appendices

[Appendix 1: PRISMA 2020 Checklist 2](#_Toc180776883)

[Appendix 2: Description of search strategies 6](#_Toc180776884)

[Appendix 3: Data extraction form 25](#_Toc180776885)

[Appendix 4: AMSTAR-2 Methodology Quality Appraisal. Adapted for application to reviews of prognostic model and accuracy studies. 27](#_Toc180776886)

[Appendix 5: Detailed results tables 32](#_Toc180776887)

[Table S1. Full-text articles excluded, with reasons 32](#_Toc180776888)

[Table S2. Systematic review characteristics 35](#_Toc180776889)

[Table S3. AMSTAR-2 assessment results per review 43](#_Toc180776890)

[Table S4. Risk prediction tool characteristics, ascertained at review level 48](#_Toc180776891)

[Table S5. Prognostic model external validation study characteristics 72](#_Toc180776892)

[Table S6. Table of Predictors, by tool (predictors were reported for 66 tools), ascertained at review level except in the case of discrepancies between reviews 73](#_Toc180776893)

## Appendix 1: PRISMA 2020 Checklist

| **Section and Topic** | **Item #** | **Checklist item** | **Location where item is reported** |
| --- | --- | --- | --- |
| **TITLE** | | |  |
| Title | 1 | Identify the report as a systematic review. | Title page and abstract |
| **ABSTRACT** | | |  |
| Abstract | 2 | See the PRISMA 2020 for Abstracts checklist. | Abstract |
| **INTRODUCTION** | | |  |
| Rationale | 3 | Describe the rationale for the review in the context of existing knowledge. | Introduction |
| Objectives | 4 | Provide an explicit statement of the objective(s) or question(s) the review addresses. | Introduction |
| **METHODS** | | |  |
| Eligibility criteria | 5 | Specify the inclusion and exclusion criteria for the review and how studies were grouped for the syntheses. | Methods: Literature search, Eligibility criteria for this umbrella review, Synthesis methods |
| Information sources | 6 | Specify all databases, registers, websites, organisations, reference lists and other sources searched or consulted to identify studies. Specify the date when each source was last searched or consulted. | Methods: Literature search  Appendix 2: Description of search strategies |
| Search strategy | 7 | Present the full search strategies for all databases, registers and websites, including any filters and limits used. | Appendix 2: Description of search strategies |
| Selection process | 8 | Specify the methods used to decide whether a study met the inclusion criteria of the review, including how many reviewers screened each record and each report retrieved, whether they worked independently, and if applicable, details of automation tools used in the process. | Methods: Literature search |
| Data collection process | 9 | Specify the methods used to collect data from reports, including how many reviewers collected data from each report, whether they worked independently, any processes for obtaining or confirming data from study investigators, and if applicable, details of automation tools used in the process. | Methods: Data extraction and quality assessment |
| Data items | 10a | List and define all outcomes for which data were sought. Specify whether all results that were compatible with each outcome domain in each study were sought (e.g. for all measures, time points, analyses), and if not, the methods used to decide which results to collect. | Methods: Data extraction and quality assessment, Synthesis methods  Appendix 3: Data extraction form |
|  | 10b | List and define all other variables for which data were sought (e.g. participant and intervention characteristics, funding sources). Describe any assumptions made about any missing or unclear information. | Appendix 3: Data extraction form  Methods: Synthesis |
| Study risk of bias assessment | 11 | Specify the methods used to assess risk of bias in the included studies, including details of the tool(s) used, how many reviewers assessed each study and whether they worked independently, and if applicable, details of automation tools used in the process. | Methods: Data extraction and quality assessment  Appendix 4: AMSTAR-2 Methodology Quality Appraisal. Adapted for application to reviews of prognostic model and accuracy studies. |
| Effect measures | 12 | Specify for each outcome the effect measure(s) (e.g. risk ratio, mean difference) used in the synthesis or presentation of results. | Methods: Synthesis methods |
| Synthesis methods | 13a | Describe the processes used to decide which studies were eligible for each synthesis (e.g. tabulating the study intervention characteristics and comparing against the planned groups for each synthesis (item #5)). | Methods: Synthesis methods |
|  | 13b | Describe any methods required to prepare the data for presentation or synthesis, such as handling of missing summary statistics, or data conversions. | -- |
|  | 13c | Describe any methods used to tabulate or visually display results of individual studies and syntheses. | Methods: Synthesis methods |
|  | 13d | Describe any methods used to synthesize results and provide a rationale for the choice(s). If meta-analysis was performed, describe the model(s), method(s) to identify the presence and extent of statistical heterogeneity, and software package(s) used. | Methods: Synthesis methods |
|  | 13e | Describe any methods used to explore possible causes of heterogeneity among study results (e.g. subgroup analysis, meta-regression). | -- |
|  | 13f | Describe any sensitivity analyses conducted to assess robustness of the synthesized results. | -- |
| Reporting bias assessment | 14 | Describe any methods used to assess risk of bias due to missing results in a synthesis (arising from reporting biases). | -- |
| Certainty assessment | 15 | Describe any methods used to assess certainty (or confidence) in the body of evidence for an outcome. | -- |
| **RESULTS** | | |  |
| Study selection | 16a | Describe the results of the search and selection process, from the number of records identified in the search to the number of studies included in the review, ideally using a flow diagram. | Results: Characteristics of included reviews  **Figure 1:** PRISMA flowchart |
|  | 16b | Cite studies that might appear to meet the inclusion criteria, but which were excluded, and explain why they were excluded. | **Appendix 5: Table S1.** Full-text articles excluded, with reasons |
| Study characteristics | 17 | Cite each included study and present its characteristics. | **Table 1.** Summary of included systematic review characteristics  **Appendix 5: Table S2.** Systematic review characteristics |
| Risk of bias in studies | 18 | Present assessments of risk of bias for each included study. | **Figure 2.** Summary of AMSTAR-2 assessment results  **Appendix 5: Table S3.** AMSTAR-2 assessment results per review |
| Results of individual studies | 19 | For all outcomes, present, for each study: (a) summary statistics for each group (where appropriate) and (b) an effect estimate and its precision (e.g. confidence/credible interval), ideally using structured tables or plots. | **Table 2.** Results of reviews reporting model development and validation  **Table 3.** Summary of tool characteristics, from review-level data |
| Results of syntheses | 20a | For each synthesis, briefly summarise the characteristics and risk of bias among contributing studies. | Results: Characteristics of included reviews, Methodological quality of included reviews  **Table 1.** Summary of included systematic review characteristics  **Figure 2.** Summary of AMSTAR-2 assessment results |
|  | 20b | Present results of all ~~statistical~~ syntheses conducted. If meta-analysis was done, present for each the summary estimate and its precision (e.g. confidence/credible interval) and measures of statistical heterogeneity. If comparing groups, describe the direction of the effect. | Results: Findings, Included tools and predictors  **Table 4.** Predictor categories and frequency (%) of inclusion in N=53 models.  **Appendix 5: Tables S4-S6** |
|  | 20c | Present results of all investigations of possible causes of heterogeneity among study results. | -- |
|  | 20d | Present results of all sensitivity analyses conducted to assess the robustness of the synthesized results. | -- |
| Reporting biases | 21 | Present assessments of risk of bias due to missing results (arising from reporting biases) for each synthesis assessed. | -- |
| Certainty of evidence | 22 | Present assessments of certainty (or confidence) in the body of evidence for each outcome assessed. | -- |
| **DISCUSSION** | | |  |
| Discussion | 23a | Provide a general interpretation of the results in the context of other evidence. | Discussion  Discussion: Other existing evidence |
|  | 23b | Discuss any limitations of the evidence included in the review. | Discussion: Strengths and limitations |
|  | 23c | Discuss any limitations of the review processes used. | Discussion: Strengths and limitations |
|  | 23d | Discuss implications of the results for practice, policy, and future research. | Discussion, Conclusions |
| **OTHER INFORMATION** | | |  |
| Registration and protocol | 24a | Provide registration information for the review, including register name and registration number, or state that the review was not registered. | Methods: Protocol registration and reporting of findings  OSF <https://osf.io/>tepyk |
|  | 24b | Indicate where the review protocol can be accessed, or state that a protocol was not prepared. | Methods: Protocol registration and reporting of findings  OSF <https://osf.io/>tepyk |
|  | 24c | Describe and explain any amendments to information provided at registration or in the protocol. | -- |
| Support | 25 | Describe sources of financial or non-financial support for the review, and the role of the funders or sponsors in the review. | Funding |
| Competing interests | 26 | Declare any competing interests of review authors. | Conflicting Interests |
| Availability of data, code and other materials | 27 | Report which of the following are publicly available and where they can be found: template data collection forms; data extracted from included studies; data used for all analyses; analytic code; any other materials used in the review. | **Appendix 3:** Data extraction form  **Appendix 5: Table S4-S6** |

*From:*  Page MJ, McKenzie JE, Bossuyt PM, Boutron I, Hoffmann TC, Mulrow CD, et al. The PRISMA 2020 statement: an updated guideline for reporting systematic reviews. BMJ 2021;372:n71. doi: 10.1136/bmj.n71

For more information, visit: <http://www.prisma-statement.org/>

## Appendix 2: Description of search strategies

ORIGINAL SEARCH: JAN 2023

**Summary table of searches**

| **Source** | **Results before deduplication** | **Results after deduplication** |
| --- | --- | --- |
| MEDLINE | 1643 | 574 |
| EMBASE | 2060 | 1920 |
| CINAHL | 3720 | 3007 |
| EPISTEMONIKOS | 1194 | 574 |
| GOOGLE SCHOLAR | 357 | 226 |
| **TOTAL** | **8974** | **6301** |

SEARCH UPDATE: JUNE 2024

**Searches run from 01/01/23 - 06/24**

**Summary table of searches**

| **Source** | **Results before deduplication** | **Results after deduplication** |
| --- | --- | --- |
| MEDLINE | 229 | 186 |
| EMBASE | 330 | 175 |
| CINAHL | 480 | 383 |
| EPISTEMONIKOS | 232 | 118 |
| GOOGLE SCHOLAR | 80 | 36 |
| **TOTAL** | **1351** | **898** |

**Search approach and sources**

Search concepts:

1. pressure injury terms
2. systematic review terms
3. prediction model terms

Pressure injury (PI) terms were used from previous PI topic reviews and were developed in consultation with the wider review team and customer.

Established systematic review methodological filters were used in OVID Embase and OVID MEDLINE combining the appropriate McMasters best balance reviews filters^[[1]](#footnote-2)^ combining the appropriate McMasters best balance systematic reviews filters^[[2]](#footnote-3)^ with the appropriate CADTH systematic review filter^[[3]](#footnote-4)^.

A number of existing methodological filters are available for prediction/prognostic model terms. The effect of using different combinations of these filters have been tested in order to ensure retrieval of relevant literature at a manageable volume. This testing has informed the choice of prognostic search filters used (Geersing)^[[4]](#footnote-5)^ Haynes Best Balance^[[5]](#footnote-6)^ and Ingui Best Balance^[[6]](#footnote-7)^.

Searches were run in OVID MEDLINE, OVID Embase and EBSCO CINAHL Plus using the search concepts, systematic review and prediction/prognostic filters listed above or adaptations of these filters. No publication date or language restrictions were applied.

Epistemonikos was also searched using PI terms and key prognostic terms limited by publication type systematic review or broad synthesis. The Epistemonikos interface does not support the same search functionality available in OVID or EBSCO (for example adjacency operators are not supported). The Information Specialist ran several separate shorter searches to accommodate for the limitations of the interface. No publication date or language restrictions were applied.

In addition, Google Scholar was searched to pick up any potentially relevant papers not indexed in the other databases. The Google Scholar interface has limited search functionality. The Information Specialist ran several separate shorter searches to accommodate for the limitations of the interface. Searches were limited to review publication types published in the last eleven years only for pragmatic reasons as Google Scholar has poor export functionality.

“Connected papers” was also considered for inclusion, however it is a one ‘seed tool’, i.e., searching for one paper generates one map of connected papers. The platform is also only freely accessible for searching five ‘seed’ papers a month, which appears to be more of a limitation than would be beneficial for this set of reviews.

**MEDLINE ALL (OVID)**

**Date run: 31/01/23**

Database: Ovid MEDLINE(R) ALL <1946 to January 30, 2023>

Search Strategy:

--------------------------------------------------------------------------------

1 (decubit* or bedsore* or bed-sore* or pressure-ulcer* or pressure-wound*).tw. (17989)

2 ((pressure* or bed or bedbound or bed-bound or bedridden or bed-ridden or deep tissue* or deep-tissue) adj3 (wound* or ulcer* or sore* or injur* or lesion*)).tw. (22136)

3 exp pressure ulcer/ or pressure/ae (15198)

4 1 or 2 or 3 (33198)

5 ((supine or immobil*) adj3 (heal or healing or heals or healed or dress*)).tw. (220)

6 ((supine or immobil*) adj3 (wound* or ulcer* or sore* or injur* or lesion*)).tw. (876)

7 ((pressure or bedbound or bedridden or bed-bound or bed-ridden or deep tissue or deep-tissue) adj3 (heal or healing or heals or healed or dress*)).tw. (1863)

8 5 or 6 or 7 (2923)

9 4 or 8 (34931)

10 (systematic review or meta-analysis).pt. (299072)

11 review.pt. (3115171)

12 search:.tw. (617577)

13 meta-analys:.mp. (291117)

14 meta-analysis/ or systematic review/ or systematic reviews as topic/ or meta-analysis as topic/ or "meta analysis (topic)"/ or "systematic review (topic)"/ or exp technology assessment, biomedical/ or network meta-analysis/ (336517)

15 ((systematic* adj3 (review* or overview*)) or (methodologic* adj3 (review* or overview*))).ti,ab,kf. (303236)

16 ((quantitative adj3 (review* or overview* or synthes*)) or (research adj3 (integrati* or overview*))).ti,ab,kf. (15021)

17 ((integrative adj3 (review* or overview*)) or (collaborative adj3 (review* or overview*)) or (pool* adj3 analy*)).ti,ab,kf. (37395)

18 (data synthes* or data extraction* or data abstraction*).ti,ab,kf. (38583)

19 (handsearch* or hand search*).ti,ab,kf. (10921)

20 (mantel haenszel or peto or der simonian or dersimonian or fixed effect* or latin square*).ti,ab,kf. (34465)

21 (met analy* or metanaly* or technology assessment* or HTA or HTAs or technology overview* or technology appraisal*).ti,ab,kf. (11813)

22 (meta regression* or metaregression*).ti,ab,kf. (13870)

23 (meta-analy* or metaanaly* or systematic review* or biomedical technology assessment* or bio-medical technology assessment*).mp,hw. (446528)

24 (medline or cochrane or pubmed or medlars or embase or cinahl).ti,ab,hw. (325867)

25 (cochrane or (health adj2 technology assessment) or evidence report).jw. (21207)

26 (comparative adj3 (efficacy or effectiveness)).ti,ab,kf. (17070)

27 (outcomes research or relative effectiveness).ti,ab,kf. (11017)

28 ((indirect or indirect treatment or mixed-treatment or bayesian) adj3 comparison*).ti,ab,kf. (4214)

29 (multi* adj3 treatment adj3 comparison*).ti,ab,kf. (287)

30 (mixed adj3 treatment adj3 (meta-analy* or metaanaly*)).ti,ab,kf. (177)

31 umbrella review*.ti,ab,kf. (1305)

32 (multi* adj2 paramet* adj2 evidence adj2 synthesis).ti,ab,kf. (13)

33 (multiparamet* adj2 evidence adj2 synthesis).ti,ab,kf. (18)

34 (multi-paramet* adj2 evidence adj2 synthesis).ti,ab,kf. (11)

35 or/10-34 (3717455)

36 predict:.tw. or validat:.mp. or develop.tw. (3128744)

37 (stratification or ROC curve).ti,ab. or exp ROC curve/ or discriminat$.ti,ab. or c-statistic.ti,ab. or "Area under the curve".ti,ab. or AUC.ti,ab. or Calibration.ti,ab. or indices.ti,ab. or algorithm.ti,ab. or multivaria$.mp. (1567005)

38 Validat*.mp. or Predict$.ti. or Rule*.mp. or (Predict* and (Outcome* or Risk* or Model$)).mp. or ((History or Variable$ or Criteria or Scor$ or Characteristic$ or Finding$ or Factor$) and (Predict$ or Model$ or Decision$ or Identif$ or Prognos$)).mp. or (Decision$.mp. and ((Model$ or Clinical$).mp. or Logistic Models/)) or (Prognostic and (History or Variable$ or Criteria or Scor$ or Characteristic$ or Finding$ or Factor$ or Model$)).mp. [mp=title, book title, abstract, original title, name of substance word, subject heading word, floating sub-heading word, keyword heading word, organism supplementary concept word, protocol supplementary concept word, rare disease supplementary concept word, unique identifier, synonyms] (5961362)

39 36 or 37 (6673746)

40 39 or 38 (7368991)

41 9 and 35 and 40 (1643)

**MEDLINE ALL (OVID)**

**Date run: 20/06/24**

Database: Ovid MEDLINE(R) ALL <1946 to June 20, 2024>

Search Strategy:

--------------------------------------------------------------------------------

1 (decubit* or bedsore* or bed-sore* or pressure-ulcer* or pressure-wound*).tw.

2 ((pressure* or bed or bedbound or bed-bound or bedridden or bed-ridden or deep tissue* or deep-tissue) adj3 (wound* or ulcer* or sore* or injur* or lesion*)).tw.

3 exp pressure ulcer/ or pressure/ae

4 1 or 2 or 3

5 ((supine or immobil*) adj3 (heal or healing or heals or healed or dress*)).tw.

6 ((supine or immobil*) adj3 (wound* or ulcer* or sore* or injur* or lesion*)).tw.

7 ((pressure or bedbound or bedridden or bed-bound or bed-ridden or deep tissue or deep-tissue) adj3 (heal or healing or heals or healed or dress*)).tw.

8 5 or 6 or 7

9 4 or 8

10 (systematic review or meta-analysis).pt.

11 review.pt.

12 search:.tw.

13 meta-analys:.mp.

14 meta-analysis/ or systematic review/ or systematic reviews as topic/ or meta-analysis as topic/ or "meta analysis (topic)"/ or "systematic review (topic)"/ or exp technology assessment, biomedical/ or network meta-analysis/

15 ((systematic* adj3 (review* or overview*)) or (methodologic* adj3 (review* or overview*))).ti,ab,kf.

16 ((quantitative adj3 (review* or overview* or synthes*)) or (research adj3 (integrati* or overview*))).ti,ab,kf.

17 ((integrative adj3 (review* or overview*)) or (collaborative adj3 (review* or overview*)) or (pool* adj3 analy*)).ti,ab,kf.

18 (data synthes* or data extraction* or data abstraction*).ti,ab,kf.

19 (handsearch* or hand search*).ti,ab,kf.

20 (mantel haenszel or peto or der simonian or dersimonian or fixed effect* or latin square*).ti,ab,kf.

21 (met analy* or metanaly* or technology assessment* or HTA or HTAs or technology overview* or technology appraisal*).ti,ab,kf.

22 (meta regression* or metaregression*).ti,ab,kf.

23 (meta-analy* or metaanaly* or systematic review* or biomedical technology assessment* or bio-medical technology assessment*).mp,hw.

24 (medline or cochrane or pubmed or medlars or embase or cinahl).ti,ab,hw.

25 (cochrane or (health adj2 technology assessment) or evidence report).jw.

26 (comparative adj3 (efficacy or effectiveness)).ti,ab,kf.

27 (outcomes research or relative effectiveness).ti,ab,kf.

28 ((indirect or indirect treatment or mixed-treatment or bayesian) adj3 comparison*).ti,ab,kf.

29 (multi* adj3 treatment adj3 comparison*).ti,ab,kf.

30 (mixed adj3 treatment adj3 (meta-analy* or metaanaly*)).ti,ab,kf.

31 umbrella review*.ti,ab,kf.

32 (multi* adj2 paramet* adj2 evidence adj2 synthesis).ti,ab,kf.

33 (multiparamet* adj2 evidence adj2 synthesis).ti,ab,kf.

34 (multi-paramet* adj2 evidence adj2 synthesis).ti,ab,kf.

35 or/10-34

36 predict:.tw. or validat:.mp. or develop.tw.

37 (stratification or ROC curve).ti,ab. or exp ROC curve/ or discriminat$.ti,ab. or c-statistic.ti,ab. or "Area under the curve".ti,ab. or AUC.ti,ab. or Calibration.ti,ab. or indices.ti,ab. or algorithm.ti,ab. or multivaria$.mp.

38 Validat*.mp. or Predict$.ti. or Rule*.mp. or (Predict* and (Outcome* or Risk* or Model$)).mp. or ((History or Variable$ or Criteria or Scor$ or Characteristic$ or Finding$ or Factor$) and (Predict$ or Model$ or Decision$ or Identif$ or Prognos$)).mp. or (Decision$.mp. and ((Model$ or Clinical$).mp. or Logistic Models/)) or (Prognostic and (History or Variable$ or Criteria or Scor$ or Characteristic$ or Finding$ or Factor$ or Model$)).mp. [mp=title, book title, abstract, original title, name of substance word, subject heading word, floating sub-heading word, keyword heading word, organism supplementary concept word, protocol supplementary concept word, rare disease supplementary concept word, unique identifier, synonyms]

39 36 or 37

40 39 or 38

41 9 and 35 and 40

42 limit 41 to dt=20230101-20241231

43 limit 41 to ez=20230101-20241231

44 limit 42 to da=20230101-20241231

45 42 or 43 or 44

**Embase (OVID)**

**Date run: 31/01/23**

Database: Embase <1974 to 2023 January 30>

Search Strategy:

--------------------------------------------------------------------------------

1 (decubit* or bedsore* or bed-sore* or pressure-ulcer* or pressure-wound*).tw. (24186)

2 ((pressure* or bed or bedbound or bed-bound or bedridden or bed-ridden or deep tissue* or deep-tissue) adj3 (wound* or ulcer* or sore* or injur* or lesion*)).tw. (28692)

3 exp decubitus/ (24330)

4 1 or 2 or 3 (45109)

5 ((supine or immobil*) adj3 (heal or healing or heals or healed or dress*)).tw. (279)

6 ((supine or immobil*) adj3 (wound* or ulcer* or sore* or injur* or lesion*)).tw. (1181)

7 ((pressure or bedbound or bedridden or bed-bound or bed-ridden or deep tissue or deep-tissue) adj3 (heal or healing or heals or healed or dress*)).tw. (2463)

8 5 or 6 or 7 (3873)

9 4 or 8 (47397)

10 (systematic review or meta-analysis).pt. (0)

11 review.pt. (3006963)

12 search:.tw. (777617)

13 meta-analys:.mp. (420287)

14 meta-analysis/ or systematic review/ or systematic reviews as topic/ or meta-analysis as topic/ or "meta analysis (topic)"/ or "systematic review (topic)"/ or exp technology assessment, biomedical/ or network meta-analysis/ (600348)

15 ((systematic* adj3 (review* or overview*)) or (methodologic* adj3 (review* or overview*))).ti,ab,kf. (374423)

16 ((quantitative adj3 (review* or overview* or synthes*)) or (research adj3 (integrati* or overview*))).ti,ab,kf. (17492)

17 ((integrative adj3 (review* or overview*)) or (collaborative adj3 (review* or overview*)) or (pool* adj3 analy*)).ti,ab,kf. (53239)

18 (data synthes* or data extraction* or data abstraction*).ti,ab,kf. (47755)

19 (handsearch* or hand search*).ti,ab,kf. (13352)

20 (mantel haenszel or peto or der simonian or dersimonian or fixed effect* or latin square*).ti,ab,kf. (45672)

21 (met analy* or metanaly* or technology assessment* or HTA or HTAs or technology overview* or technology appraisal*).ti,ab,kf. (19758)

22 (meta regression* or metaregression*).ti,ab,kf. (17239)

23 (meta-analy* or metaanaly* or systematic review* or biomedical technology assessment* or bio-medical technology assessment*).mp,hw. (709507)

24 (medline or cochrane or pubmed or medlars or embase or cinahl).ti,ab,hw. (427516)

25 (cochrane or (health adj2 technology assessment) or evidence report).jw. (30592)

26 (comparative adj3 (efficacy or effectiveness)).ti,ab,kf. (25197)

27 (outcomes research or relative effectiveness).ti,ab,kf. (15991)

28 ((indirect or indirect treatment or mixed-treatment or bayesian) adj3 comparison*).ti,ab,kf. (7335)

29 (multi* adj3 treatment adj3 comparison*).ti,ab,kf. (423)

30 (mixed adj3 treatment adj3 (meta-analy* or metaanaly*)).ti,ab,kf. (256)

31 umbrella review*.ti,ab,kf. (1367)

32 (multi* adj2 paramet* adj2 evidence adj2 synthesis).ti,ab,kf. (28)

33 (multiparamet* adj2 evidence adj2 synthesis).ti,ab,kf. (21)

34 (multi-paramet* adj2 evidence adj2 synthesis).ti,ab,kf. (23)

35 or/10-34 (3937855)

36 validat:.mp. or index.tw. or model.tw. (5261274)

37 (stratification or ROC curve).ti,ab. or exp receiver operating characteristic/ or discriminat$.ti,ab. or c-statistic.ti,ab. or "Area under the curve".ti,ab. or AUC.ti,ab. or Calibration.ti,ab. or indices.ti,ab. or algorithm.ti,ab. or multivaria$.mp. (2151295)

38 Validat*.mp. or Predict$.ti. or Rule*.mp. or (Predict* and (Outcome* or Risk* or Model$)).mp. or ((History or Variable$ or Criteria or Scor$ or Characteristic$ or Finding$ or Factor$) and (Predict$ or Model$ or Decision$ or Identif$ or Prognos$)).mp. or (Decision$.mp. and ((Model$ or Clinical$).mp. or Statistical model/)) or (Prognostic and (History or Variable$ or Criteria or Scor$ or Characteristic$ or Finding$ or Factor$ or Model$)).mp. [mp=title, abstract, heading word, drug trade name, original title, device manufacturer, drug manufacturer, device trade name, keyword heading word, floating subheading word, candidate term word] (8141367)

39 36 or 37 (9046587)

40 39 or 38 (10998837)

47 9 and 35 and 40 (2060)

**Embase (OVID)**

**Date run: 20/06/24**

Database: Embase <1974 to 2024 June 20>

Search Strategy:

--------------------------------------------------------------------------------

1 (decubit* or bedsore* or bed-sore* or pressure-ulcer* or pressure-wound*).tw.

2 ((pressure* or bed or bedbound or bed-bound or bedridden or bed-ridden or deep tissue* or deep-tissue) adj3 (wound* or ulcer* or sore* or injur* or lesion*)).tw.

3 exp decubitus/

4 1 or 2 or 3

5 ((supine or immobil*) adj3 (heal or healing or heals or healed or dress*)).tw.

6 ((supine or immobil*) adj3 (wound* or ulcer* or sore* or injur* or lesion*)).tw.

7 ((pressure or bedbound or bedridden or bed-bound or bed-ridden or deep tissue or deep-tissue) adj3 (heal or healing or heals or healed or dress*)).tw.

8 5 or 6 or 7

9 4 or 8

10 (systematic review or meta-analysis).pt.

11 review.pt.

12 search:.tw.

13 meta-analys:.mp.

14 meta-analysis/ or systematic review/ or systematic reviews as topic/ or meta-analysis as topic/ or "meta analysis (topic)"/ or "systematic review (topic)"/ or exp technology assessment, biomedical/ or network meta-analysis/

15 ((systematic* adj3 (review* or overview*)) or (methodologic* adj3 (review* or overview*))).ti,ab,kf.

16 ((quantitative adj3 (review* or overview* or synthes*)) or (research adj3 (integrati* or overview*))).ti,ab,kf.

17 ((integrative adj3 (review* or overview*)) or (collaborative adj3 (review* or overview*)) or (pool* adj3 analy*)).ti,ab,kf.

18 (data synthes* or data extraction* or data abstraction*).ti,ab,kf.

19 (handsearch* or hand search*).ti,ab,kf.

20 (mantel haenszel or peto or der simonian or dersimonian or fixed effect* or latin square*).ti,ab,kf.

21 (met analy* or metanaly* or technology assessment* or HTA or HTAs or technology overview* or technology appraisal*).ti,ab,kf.

22 (meta regression* or metaregression*).ti,ab,kf.

23 (meta-analy* or metaanaly* or systematic review* or biomedical technology assessment* or bio-medical technology assessment*).mp,hw.

24 (medline or cochrane or pubmed or medlars or embase or cinahl).ti,ab,hw.

25 (cochrane or (health adj2 technology assessment) or evidence report).jw.

26 (comparative adj3 (efficacy or effectiveness)).ti,ab,kf.

27 (outcomes research or relative effectiveness).ti,ab,kf.

28 ((indirect or indirect treatment or mixed-treatment or bayesian) adj3 comparison*).ti,ab,kf.

29 (multi* adj3 treatment adj3 comparison*).ti,ab,kf.

30 (mixed adj3 treatment adj3 (meta-analy* or metaanaly*)).ti,ab,kf.

31 umbrella review*.ti,ab,kf.

32 (multi* adj2 paramet* adj2 evidence adj2 synthesis).ti,ab,kf.

33 (multiparamet* adj2 evidence adj2 synthesis).ti,ab,kf.

34 (multi-paramet* adj2 evidence adj2 synthesis).ti,ab,kf.

35 or/10-34

36 validat:.mp. or index.tw. or model.tw.

37 (stratification or ROC curve).ti,ab. or exp receiver operating characteristic/ or discriminat$.ti,ab. or c-statistic.ti,ab. or "Area under the curve".ti,ab. or AUC.ti,ab. or Calibration.ti,ab. or indices.ti,ab. or algorithm.ti,ab. or multivaria$.mp.

38 Validat*.mp. or Predict$.ti. or Rule*.mp. or (Predict* and (Outcome* or Risk* or Model$)).mp. or ((History or Variable$ or Criteria or Scor$ or Characteristic$ or Finding$ or Factor$) and (Predict$ or Model$ or Decision$ or Identif$ or Prognos$)).mp. or (Decision$.mp. and ((Model$ or Clinical$).mp. or Statistical model/)) or (Prognostic and (History or Variable$ or Criteria or Scor$ or Characteristic$ or Finding$ or Factor$ or Model$)).mp. [mp=title, abstract, heading word, drug trade name, original title, device manufacturer, drug manufacturer, device trade name, keyword heading word, floating subheading word, candidate term word] (8141367)

39 36 or 37

40 39 or 38

47 9 and 35 and 40

48 limit 47 to dc=20230101-20243112

**CINAHL PLUS (EBSCOhost)***SEARCH DATE 02/02/23*

S1 ( ((TI decubit* OR AB decubit*) OR (TI bedsore* OR AB bedsore*) OR (TI bed-sore* OR AB bed-sore*) OR (TI pressure-ulcer* OR AB pressure-ulcer*) OR (TI pressure-wound* OR AB pressure-wound*)) ) OR ( (((TI pressure* OR AB pressure*) OR (TI bed OR AB bed) OR (TI bedbound OR AB bedbound) OR (TI bed-bound OR AB bed-bound) OR (TI bedridden OR AB bedridden) OR (TI bed-ridden OR AB bed-ridden) OR (TI "deep tissue*" OR AB "deep tissue*") OR (TI deep-tissue OR AB deep-tissue)) N3 ((TI wound* OR AB wound*) OR (TI ulcer* OR AB ulcer*) OR (TI sore* OR AB sore*) OR (TI injur* OR AB injur*) OR (TI lesion* OR AB lesion*))) ) OR ( (MH "pressure ulcer"+) OR (MH pressure) ) Expanders - Apply equivalent subjects (28 401)

S2 ( ((TI heal OR AB heal) OR (TI healing OR AB healing) OR (TI heals OR AB heals) OR (TI healed OR AB healed) OR (TI dress* OR AB dress*))) ) OR ( (((TI supine OR AB supine) OR (TI immobil* OR AB immobil*)) N3 ((TI wound* OR AB wound*) OR (TI ulcer* OR AB ulcer*) OR (TI sore* OR AB sore*) OR (TI injur* OR AB injur*) OR (TI lesion* OR AB lesion*))) ) OR ( (((TI pressure OR AB pressure) OR (TI bedbound OR AB bedbound) OR (TI bedridden OR AB bedridden) OR (TI bed-bound OR AB bed-bound) OR (TI bed-ridden OR AB bed-ridden) OR (TI "deep tissue" OR AB "deep tissue") OR (TI deep-tissue OR AB deep-tissue)) N3 ((TI heal OR AB heal) OR (TI healing OR AB healing) OR (TI heals OR AB heals) OR (TI healed OR AB healed) OR (TI dress* OR AB dress*))) ) Expanders – Apply equivalent subjects (72 286)

S3 S1 OR S2 Expanders - Apply equivalent subjects (96245)

S4 ( (PT "systematic review" OR PT meta-analysis) ) OR ( (MH meta-analysis) OR (MH "systematic review") OR (MH "systematic reviews as topic") OR (MH "meta-analysis as topic") OR (MH "meta analysis (topic)") OR (MH "systematic review (topic)") OR (MH "technology assessment, biomedical"+) OR (MH "network meta-analysis") ) OR ( (((TI systematic* OR AB systematic* OR SU systematic*) N3 ((TI review* OR AB review* OR SU review*) OR (TI overview* OR AB overview* OR SU overview*))) OR ((TI methodologic* OR AB methodologic* OR SU methodologic*) N3 ((TI review* OR AB review* OR SU review*) OR (TI overview* OR AB overview* OR SU overview*)))) ) OR ( (((TI quantitative OR AB quantitative OR SU quantitative) N3 ((TI review* OR AB review* OR SU review*) OR (TI overview* OR AB overview* OR SU overview*) OR (TI synthes* OR AB synthes* OR SU synthes*))) OR ((TI research OR AB research OR SU research) N3 ((TI integrati* OR AB integrati* OR SU integrati*) OR (TI overview* OR AB overview* OR SU overview*)))) ) OR ( (((TI integrative OR AB integrative OR SU integrative) N3 ((TI review* OR AB review* OR SU review*) OR (TI overview* OR AB overview* OR SU overview*))) OR ((TI collaborative OR AB collaborative OR SU collaborative) N3 ((TI review* OR AB review* OR SU review*) OR (TI overview* OR AB overview* OR SU overview*))) OR ((TI pool* OR AB pool* OR SU pool*) N3 (TI analy* OR AB analy* OR SU analy*))) ) OR ( ((TI "data synthes*" OR AB "data synthes*" OR SU "data synthes*") OR (TI "data extraction*" OR AB "data extraction*" OR SU "data extraction*") OR (TI "data abstraction*" OR AB "data abstraction*" OR SU "data abstraction*")) ) OR ( ((TI handsearch* OR AB handsearch* OR SU handsearch*) OR (TI "hand search*" OR AB "hand search*" OR SU "hand search*")) ) OR ( ((TI "mantel haenszel" OR AB "mantel haenszel" OR SU "mantel haenszel") OR (TI peto OR AB peto OR SU peto) OR (TI "der simonian" OR AB "der simonian" OR SU "der simonian") OR (TI dersimonian OR AB dersimonian OR SU dersimonian) OR (TI "fixed effect*" OR AB "fixed effect*" OR SU "fixed effect*") OR (TI "latin square*" OR AB "latin square*" OR SU "latin square*")) ) OR ( ((TI "met analy*" OR AB "met analy*" OR SU "met analy*") OR (TI metanaly* OR AB metanaly* OR SU metanaly*) OR (TI "technology assessment*" OR AB "technology assessment*" OR SU "technology assessment*") OR (TI HTA OR AB HTA OR SU HTA) OR (TI HTAs OR AB HTAs OR SU HTAs) OR (TI "technology overview*" OR AB "technology overview*" OR SU "technology overview*") OR (TI "technology appraisal*" OR AB "technology appraisal*" OR SU "technology appraisal*")) ) OR ( ((TI "meta regression*" OR AB "meta regression*" OR SU "meta regression*") OR (TI metaregression* OR AB metaregression* OR SU metaregression*)) ) OR ( (meta-analy* OR metaanaly* OR "systematic review*" OR "biomedical technology assessment*" OR "bio-medical technology assessment*") ,hw. ) OR ( ((TI medline OR AB medline) OR (TI cochrane OR AB cochrane) OR (TI pubmed OR AB pubmed) OR (TI medlars OR AB medlars) OR (TI embase OR AB embase) OR (TI cinahl OR AB cinahl)) ,hw. ) Expanders - Apply equivalent subjects (237782)

S5 ( (cochrane OR ( health N2 "technology assessment") OR "evidence report") .jw. ) OR ( ((TI comparative OR AB comparative OR SU comparative) N3 ((TI efficacy OR AB efficacy OR SU efficacy) OR (TI effectiveness OR AB effectiveness OR SU effectiveness))) ) OR ( ((TI "outcomes research" OR AB "outcomes research" OR SU "outcomes research") OR (TI "relative effectiveness" OR AB "relative effectiveness" OR SU "relative effectiveness")) ) OR ( (((TI indirect OR AB indirect OR SU indirect) OR (TI "indirect treatment" OR AB "indirect treatment" OR SU "indirect treatment") OR (TI mixed-treatment OR AB mixed-treatment OR SU mixed-treatment) OR (TI bayesian OR AB bayesian OR SU bayesian)) N3 (TI comparison* OR AB comparison* OR SU comparison*)) ) OR ( ((TI multi* OR AB multi* OR SU multi*) N3 (TI treatment OR AB treatment OR SU treatment) N3 (TI comparison* OR AB comparison* OR SU comparison*)) ) OR ( (TI "umbrella review*" OR AB "umbrella review*" OR SU "umbrella review*") ) OR ( ((TI multi* OR AB multi* OR SU multi*) N2 (TI paramet* OR AB paramet* OR SU paramet*) N2 (TI evidence OR AB evidence OR SU evidence) N2 (TI synthesis OR AB synthesis OR SU synthesis)) ) OR ( ((TI multiparamet* OR AB multiparamet* OR SU multiparamet*) N2 (TI evidence OR AB evidence OR SU evidence) N2 (TI synthesis OR AB synthesis OR SU synthesis)) ) OR ( ((TI multi-paramet* OR AB multi-paramet* OR SU multi-paramet*) N2 (TI evidence OR AB evidence OR SU evidence) N2 (TI synthesis OR AB synthesis OR SU synthesis)) Expanders - Apply equivalent subjects (19108)

S6 ((TI search: OR AB search:)) Expanders - Apply equivalent subjects (123419)

S7 PT review Expanders - Apply equivalent subjects (356003)

S8 S4 OR S5 OR S6 OR S7 Expanders - Apply equivalent subjects (645360)

S9 S3 AND S8 Expanders - Apply equivalent subjects (10379)

S100 (TX validat*) or (TI index or model) or (AB index or model) Expanders - Apply equivalent subjects (1286240)xpanders - Apply

S11 TI ( stratification or "ROC curve" or discriminat" or c-statistic" or "Area under the curve" or AUC or Calibration* or indices* or algorithm* or multivaria* ) OR AB ( stratification or "ROC curve" or discriminat" or c-statistic" or "Area under the curve" or AUC or Calibration* or indices* or algorithm* or multivaria* ) Expanders - Apply equivalent subjects (294871)

S12 (MH "ROC Curve") Expanders - Apply equivalent subjects (33393)

S13 TI Validat* or Predict* or Rule* or (Predict* and (Outcome* or Risk* or Model*)) or ((History or Variable* or Criteria or Scor* or Characteristic* or Finding* or Factor*) and (Predict* or Model* or Decision* or Identif* or Prognos*)) or (Decision* and ((Model* or Clinical*) or (Prognostic and (History or Variable* or Criteria or Scor* or Characteristic* or Finding* or Factor* or Model*)) Expanders - Apply equivalent subjects (144228)

S14 AB Validat* or Predict* or Rule* or (Predict* and (Outcome* or Risk* or Model*)) or ((History or Variable* or Criteria or Scor* or Characteristic* or Finding* or Factor*) and (Predict* or Model* or Decision* or Identif* or Prognos*)) or (Decision* and ((Model* or Clinical*) or (Prognostic and (History or Variable* or Criteria or Scor* or Characteristic* or Finding* or Factor* or Model*)) Expanders - Apply equivalent subjects (1482692)

S15 (MH "Models, Statistical+") Expanders - Apply equivalent subjects (40243)

S16 S10 OR S11 OR S12 OR S13 OR S14 OR S15 Expanders - Apply equivalent subjects (2119713)

S17 S9 AND S16 (3720)

**CINAHL PLUS (EBSCOhost)***SEARCH DATE 21/06/24*

S1 ( ((TI decubit* OR AB decubit*) OR (TI bedsore* OR AB bedsore*) OR (TI bed-sore* OR AB bed-sore*) OR (TI pressure-ulcer* OR AB pressure-ulcer*) OR (TI pressure-wound* OR AB pressure-wound*)) ) OR ( (((TI pressure* OR AB pressure*) OR (TI bed OR AB bed) OR (TI bedbound OR AB bedbound) OR (TI bed-bound OR AB bed-bound) OR (TI bedridden OR AB bedridden) OR (TI bed-ridden OR AB bed-ridden) OR (TI "deep tissue*" OR AB "deep tissue*") OR (TI deep-tissue OR AB deep-tissue)) N3 ((TI wound* OR AB wound*) OR (TI ulcer* OR AB ulcer*) OR (TI sore* OR AB sore*) OR (TI injur* OR AB injur*) OR (TI lesion* OR AB lesion*))) ) OR ( (MH "pressure ulcer"+) OR (MH pressure) ) Expanders - Apply equivalent subjects

S2 ( ((TI heal OR AB heal) OR (TI healing OR AB healing) OR (TI heals OR AB heals) OR (TI healed OR AB healed) OR (TI dress* OR AB dress*))) ) OR ( (((TI supine OR AB supine) OR (TI immobil* OR AB immobil*)) N3 ((TI wound* OR AB wound*) OR (TI ulcer* OR AB ulcer*) OR (TI sore* OR AB sore*) OR (TI injur* OR AB injur*) OR (TI lesion* OR AB lesion*))) ) OR ( (((TI pressure OR AB pressure) OR (TI bedbound OR AB bedbound) OR (TI bedridden OR AB bedridden) OR (TI bed-bound OR AB bed-bound) OR (TI bed-ridden OR AB bed-ridden) OR (TI "deep tissue" OR AB "deep tissue") OR (TI deep-tissue OR AB deep-tissue)) N3 ((TI heal OR AB heal) OR (TI healing OR AB healing) OR (TI heals OR AB heals) OR (TI healed OR AB healed) OR (TI dress* OR AB dress*))) ) Expanders – Apply equivalent subjects

S3 S1 OR S2 Expanders - Apply equivalent subjects

S4 ( (PT "systematic review" OR PT meta-analysis) ) OR ( (MH meta-analysis) OR (MH "systematic review") OR (MH "systematic reviews as topic") OR (MH "meta-analysis as topic") OR (MH "meta analysis (topic)") OR (MH "systematic review (topic)") OR (MH "technology assessment, biomedical"+) OR (MH "network meta-analysis") ) OR ( (((TI systematic* OR AB systematic* OR SU systematic*) N3 ((TI review* OR AB review* OR SU review*) OR (TI overview* OR AB overview* OR SU overview*))) OR ((TI methodologic* OR AB methodologic* OR SU methodologic*) N3 ((TI review* OR AB review* OR SU review*) OR (TI overview* OR AB overview* OR SU overview*)))) ) OR ( (((TI quantitative OR AB quantitative OR SU quantitative) N3 ((TI review* OR AB review* OR SU review*) OR (TI overview* OR AB overview* OR SU overview*) OR (TI synthes* OR AB synthes* OR SU synthes*))) OR ((TI research OR AB research OR SU research) N3 ((TI integrati* OR AB integrati* OR SU integrati*) OR (TI overview* OR AB overview* OR SU overview*)))) ) OR ( (((TI integrative OR AB integrative OR SU integrative) N3 ((TI review* OR AB review* OR SU review*) OR (TI overview* OR AB overview* OR SU overview*))) OR ((TI collaborative OR AB collaborative OR SU collaborative) N3 ((TI review* OR AB review* OR SU review*) OR (TI overview* OR AB overview* OR SU overview*))) OR ((TI pool* OR AB pool* OR SU pool*) N3 (TI analy* OR AB analy* OR SU analy*))) ) OR ( ((TI "data synthes*" OR AB "data synthes*" OR SU "data synthes*") OR (TI "data extraction*" OR AB "data extraction*" OR SU "data extraction*") OR (TI "data abstraction*" OR AB "data abstraction*" OR SU "data abstraction*")) ) OR ( ((TI handsearch* OR AB handsearch* OR SU handsearch*) OR (TI "hand search*" OR AB "hand search*" OR SU "hand search*")) ) OR ( ((TI "mantel haenszel" OR AB "mantel haenszel" OR SU "mantel haenszel") OR (TI peto OR AB peto OR SU peto) OR (TI "der simonian" OR AB "der simonian" OR SU "der simonian") OR (TI dersimonian OR AB dersimonian OR SU dersimonian) OR (TI "fixed effect*" OR AB "fixed effect*" OR SU "fixed effect*") OR (TI "latin square*" OR AB "latin square*" OR SU "latin square*")) ) OR ( ((TI "met analy*" OR AB "met analy*" OR SU "met analy*") OR (TI metanaly* OR AB metanaly* OR SU metanaly*) OR (TI "technology assessment*" OR AB "technology assessment*" OR SU "technology assessment*") OR (TI HTA OR AB HTA OR SU HTA) OR (TI HTAs OR AB HTAs OR SU HTAs) OR (TI "technology overview*" OR AB "technology overview*" OR SU "technology overview*") OR (TI "technology appraisal*" OR AB "technology appraisal*" OR SU "technology appraisal*")) ) OR ( ((TI "meta regression*" OR AB "meta regression*" OR SU "meta regression*") OR (TI metaregression* OR AB metaregression* OR SU metaregression*)) ) OR ( (meta-analy* OR metaanaly* OR "systematic review*" OR "biomedical technology assessment*" OR "bio-medical technology assessment*") ,hw. ) OR ( ((TI medline OR AB medline) OR (TI cochrane OR AB cochrane) OR (TI pubmed OR AB pubmed) OR (TI medlars OR AB medlars) OR (TI embase OR AB embase) OR (TI cinahl OR AB cinahl)) ,hw. )

S5 ( (cochrane OR ( health N2 "technology assessment") OR "evidence report") .jw. ) OR ( ((TI comparative OR AB comparative OR SU comparative) N3 ((TI efficacy OR AB efficacy OR SU efficacy) OR (TI effectiveness OR AB effectiveness OR SU effectiveness))) ) OR ( ((TI "outcomes research" OR AB "outcomes research" OR SU "outcomes research") OR (TI "relative effectiveness" OR AB "relative effectiveness" OR SU "relative effectiveness")) ) OR ( (((TI indirect OR AB indirect OR SU indirect) OR (TI "indirect treatment" OR AB "indirect treatment" OR SU "indirect treatment") OR (TI mixed-treatment OR AB mixed-treatment OR SU mixed-treatment) OR (TI bayesian OR AB bayesian OR SU bayesian)) N3 (TI comparison* OR AB comparison* OR SU comparison*)) ) OR ( ((TI multi* OR AB multi* OR SU multi*) N3 (TI treatment OR AB treatment OR SU treatment) N3 (TI comparison* OR AB comparison* OR SU comparison*)) ) OR ( (TI "umbrella review*" OR AB "umbrella review*" OR SU "umbrella review*") ) OR ( ((TI multi* OR AB multi* OR SU multi*) N2 (TI paramet* OR AB paramet* OR SU paramet*) N2 (TI evidence OR AB evidence OR SU evidence) N2 (TI synthesis OR AB synthesis OR SU synthesis)) ) OR ( ((TI multiparamet* OR AB multiparamet* OR SU multiparamet*) N2 (TI evidence OR AB evidence OR SU evidence) N2 (TI synthesis OR AB synthesis OR SU synthesis)) ) OR ( ((TI multi-paramet* OR AB multi-paramet* OR SU multi-paramet*) N2 (TI evidence OR AB evidence OR SU evidence) N2 (TI synthesis OR AB synthesis OR SU synthesis))

S6 ((TI search: OR AB search:))

S7 PT review

S8 S4 OR S5 OR S6 OR S7

S9 S3 AND S8

S100 (TX validat*) or (TI index or model) or (AB index or model) s - Apply

S11 TI ( stratification or "ROC curve" or discriminat" or c-statistic" or "Area under the curve" or AUC or Calibration* or indices* or algorithm* or multivaria* ) OR AB ( stratification or "ROC curve" or discriminat" or c-statistic" or "Area under the curve" or AUC or Calibration* or indices* or algorithm* or multivaria* )

S12 (MH "ROC Curve")

S13 TI Validat* or Predict* or Rule* or (Predict* and (Outcome* or Risk* or Model*)) or ((History or Variable* or Criteria or Scor* or Characteristic* or Finding* or Factor*) and (Predict* or Model* or Decision* or Identif* or Prognos*)) or (Decision* and ((Model* or Clinical*) or (Prognostic and (History or Variable* or Criteria or Scor* or Characteristic* or Finding* or Factor* or Model*))

S14 AB Validat* or Predict* or Rule* or (Predict* and (Outcome* or Risk* or Model*)) or ((History or Variable* or Criteria or Scor* or Characteristic* or Finding* or Factor*) and (Predict* or Model* or Decision* or Identif* or Prognos*)) or (Decision* and ((Model* or Clinical*) or (Prognostic and (History or Variable* or Criteria or Scor* or Characteristic* or Finding* or Factor* or Model*))

S15 (MH "Models, Statistical+")

S16 S10 OR S11 OR S12 OR S13 OR S14 OR S15

S17 S9 AND S16

S18 (EM 20230101-20241212) OR (ZD "in process" AND RD 20230101-20241212)

S19 S17 AND S18

**EPISTEMONIKOS**

**Date run: 31/01/23**

**Date update run: 21/06/24**

For update, all searches limited by date added to database: From: 01/01/2023 To: 21/06/2024

**Search 1**

(title:(decubit* OR bedsore* OR bed-sore* OR pressure-ulcer* OR pressure-wound*) OR abstract:(decubit* OR bedsore* OR bed-sore* OR pressure-ulcer* OR pressure-wound*)) AND (title:(stratification OR "ROC curve" OR "ROC curves" OR "receiver operating characteristic" OR discriminat* OR c-statistic OR "Area under the curve" OR AUC OR Calibration OR indices OR algorithm OR multivaria* OR Validat* OR Predict* OR Rule* OR Risk* OR Model* OR Criteria OR Scor* OR Characteristic* OR Finding* OR Factor* OR Decision* OR Prognos* OR Index OR model OR prevent*) OR abstract:(stratification OR "ROC curve" OR "ROC curves" OR "receiver operating characteristic" OR discriminat* OR c-statistic OR "Area under the curve" OR AUC OR Calibration OR indices OR algorithm OR multivaria* OR Validat* OR Predict* OR Rule* OR Risk* OR Model* OR Criteria OR Scor* OR Characteristic* OR Finding* OR Factor* OR Decision* OR Prognos* OR Index OR model OR prevent*))
Limit by publication type: systematic review (106) (Update: 21) or broad synthesis (3) (Update: 0)

**Search 2**

(title:("pressure ulcer" OR "pressure ulcers" OR "pressure sore" OR "pressure sores" OR "pressure lesion" OR "pressure lesions" OR "pressure injury" OR "pressure injuries") OR abstract:("pressure ulcer" OR "pressure ulcers" OR "pressure sore" OR "pressure sores" OR "pressure lesion" OR "pressure lesions" OR "pressure injury" OR "pressure injuries")) AND (title:(stratification OR "ROC curve" OR "ROC curves" OR "receiver operating characteristic" OR discriminat* OR c-statistic OR "Area under the curve" OR AUC OR Calibration OR indices OR algorithm OR multivaria* OR Validat* OR Predict* OR Rule* OR Risk* OR Model* OR Criteria OR Scor* OR Characteristic* OR Finding* OR Factor* OR Decision* OR Prognos* OR Index OR model OR prevent*) OR abstract:(stratification OR "ROC curve" OR "ROC curves" OR "receiver operating characteristic" OR discriminat* OR c-statistic OR "Area under the curve" OR AUC OR Calibration OR indices OR algorithm OR multivaria* OR Validat* OR Predict* OR Rule* OR Risk* OR Model* OR Criteria OR Scor* OR Characteristic* OR Finding* OR Factor* OR Decision* OR Prognos* OR Index OR model OR prevent*))
Limit by publication type: systematic review (709) (Update: 129) or broad synthesis (35) (Update: 25)

**Search 3**

(title:("deep-tissue wound" OR "deep-tissue wounds" OR "deep-tissue ulcer" OR "deep-tissue ulcers" OR "deep-tissue sore" OR "deep-tissue sores" OR "deep-tissue lesion" OR "deep-tissue lesions" OR "deep-tissue injury" OR "deep-tissue injuries") OR abstract:("deep-tissue wound" OR "deep-tissue wounds" OR "deep-tissue ulcer" OR "deep-tissue ulcers" OR "deep-tissue sore" OR "deep-tissue sores" OR "deep-tissue lesion" OR "deep-tissue lesions" OR "deep-tissue injury" OR "deep-tissue injuries")) AND (title:(stratification OR "ROC curve" OR "ROC curves" OR "receiver operating characteristic" OR discriminat* OR c-statistic OR "Area under the curve" OR AUC OR Calibration OR indices OR algorithm OR multivaria* OR Validat* OR Predict* OR Rule* OR Risk* OR Model* OR Criteria OR Scor* OR Characteristic* OR Finding* OR Factor* OR Decision* OR Prognos* OR Index OR model OR prevent*) OR abstract:(stratification OR "ROC curve" OR "ROC curves" OR "receiver operating characteristic" OR discriminat* OR c-statistic OR "Area under the curve" OR AUC OR Calibration OR indices OR algorithm OR multivaria* OR Validat* OR Predict* OR Rule* OR Risk* OR Model* OR Criteria OR Scor* OR Characteristic* OR Finding* OR Factor* OR Decision* OR Prognos* OR Index OR model OR prevent*))
Limit by publication type: systematic review (0) (Update: 0) or broad synthesis (0) (Update: 0)

**Search 4**

(title:("deep tissue wound" OR "deep tissue wounds" OR "deep tissue ulcer" OR "deep tissue ulcers" OR "deep tissue sore" OR "deep tissue sores" OR "deep tissue lesion" OR "deep tissue lesions" OR "deep tissue injury" OR "deep tissue injuries") OR abstract:("deep tissue wound" OR "deep tissue wounds" OR "deep tissue ulcer" OR "deep tissue ulcers" OR "deep tissue sore" OR "deep tissue sores" OR "deep tissue lesion" OR "deep tissue lesions" OR "deep tissue injury" OR "deep tissue injuries")) AND (title:(stratification OR "ROC curve" OR "ROC curves" OR "receiver operating characteristic" OR discriminat* OR c-statistic OR "Area under the curve" OR AUC OR Calibration OR indices OR algorithm OR multivaria* OR Validat* OR Predict* OR Rule* OR Risk* OR Model* OR Criteria OR Scor* OR Characteristic* OR Finding* OR Factor* OR Decision* OR Prognos* OR Index OR model OR prevent*) OR abstract:(stratification OR "ROC curve" OR "ROC curves" OR "receiver operating characteristic" OR discriminat* OR c-statistic OR "Area under the curve" OR AUC OR Calibration OR indices OR algorithm OR multivaria* OR Validat* OR Predict* OR Rule* OR Risk* OR Model* OR Criteria OR Scor* OR Characteristic* OR Finding* OR Factor* OR Decision* OR Prognos* OR Index OR model OR prevent*))
Limit by publication type: systematic review (5) (Update: 1) or broad synthesis (2) (Update: 0)

**Search 5**

(title:("bed wound" OR "bed wounds" OR "bed ulcer" OR "bed ulcers" OR "bed sore" OR "bed sores" OR "bed lesion" OR "bed lesions" OR "bed injury" OR "bed injuries") OR abstract:("bed wound" OR "bed wounds" OR "bed ulcer" OR "bed ulcers" OR "bed sore" OR "bed sores" OR "bed lesion" OR "bed lesions" OR "bed injury" OR "bed injuries")) AND (title:(stratification OR "ROC curve" OR "ROC curves" OR "receiver operating characteristic" OR discriminat* OR c-statistic OR "Area under the curve" OR AUC OR Calibration OR indices OR algorithm OR multivaria* OR Validat* OR Predict* OR Rule* OR Risk* OR Model* OR Criteria OR Scor* OR Characteristic* OR Finding* OR Factor* OR Decision* OR Prognos* OR Index OR model OR prevent*) OR abstract:(stratification OR "ROC curve" OR "ROC curves" OR "receiver operating characteristic" OR discriminat* OR c-statistic OR "Area under the curve" OR AUC OR Calibration OR indices OR algorithm OR multivaria* OR Validat* OR Predict* OR Rule* OR Risk* OR Model* OR Criteria OR Scor* OR Characteristic* OR Finding* OR Factor* OR Decision* OR Prognos* OR Index OR model OR prevent*))
Limit by publication type: systematic review (14) (Update: 0) or broad synthesis (0) (Update: 1)

**Search 6**

(title:("bed bound" OR bed-bound OR bedridden OR bed-ridden OR "bed ridden") OR abstract:("bed bound" OR bed-bound OR bedridden OR bed-ridden OR "bed ridden")) AND (title:(stratification OR "ROC curve" OR "ROC curves" OR "receiver operating characteristic" OR discriminat* OR c-statistic OR "Area under the curve" OR AUC OR Calibration OR indices OR algorithm OR multivaria* OR Validat* OR Predict* OR Rule* OR Risk* OR Model* OR Criteria OR Scor* OR Characteristic* OR Finding* OR Factor* OR Decision* OR Prognos* OR Index OR model OR prevent*) OR abstract:(stratification OR "ROC curve" OR "ROC curves" OR "receiver operating characteristic" OR discriminat* OR c-statistic OR "Area under the curve" OR AUC OR Calibration OR indices OR algorithm OR multivaria* OR Validat* OR Predict* OR Rule* OR Risk* OR Model* OR Criteria OR Scor* OR Characteristic* OR Finding* OR Factor* OR Decision* OR Prognos* OR Index OR model OR prevent*))
Limit by publication type: systematic review (30) (Update: 3) or broad synthesis (1) (Update: 1)

**Search 7**

(title:(stratification OR "ROC curve" OR "ROC curves" OR "receiver operating characteristic" OR discriminat* OR c-statistic OR "Area under the curve" OR AUC OR Calibration OR indices OR algorithm OR multivaria* OR Validat* OR Predict* OR Rule* OR Risk* OR Model* OR Criteria OR Scor* OR Characteristic* OR Finding* OR Factor* OR Decision* OR Prognos* OR Index OR model OR prevent*) OR abstract:(stratification OR "ROC curve" OR "ROC curves" OR "receiver operating characteristic" OR discriminat* OR c-statistic OR "Area under the curve" OR AUC OR Calibration OR indices OR algorithm OR multivaria* OR Validat* OR Predict* OR Rule* OR Risk* OR Model* OR Criteria OR Scor* OR Characteristic* OR Finding* OR Factor* OR Decision* OR Prognos* OR Index OR model OR prevent*)) AND (title:( wound* OR ulcer* OR sore* OR injur* OR lesion*) OR abstract:( wound* OR ulcer* OR sore* OR injur* OR lesion*)) AND (title:(supine OR immobil*) OR abstract:(supine OR immobil*))
Limit by publication type: systematic review (234) (Update: 39) or broad synthesis (13) (Update: 3)

**Search 8**

(title:(stratification OR "ROC curve" OR "ROC curves" OR "receiver operating characteristic" OR discriminat* OR c-statistic OR "Area under the curve" OR AUC OR Calibration OR indices OR algorithm OR multivaria* OR Validat* OR Predict* OR Rule* OR Risk* OR Model* OR Criteria OR Scor* OR Characteristic* OR Finding* OR Factor* OR Decision* OR Prognos* OR Index OR model OR prevent*) OR abstract:(stratification OR "ROC curve" OR "ROC curves" OR "receiver operating characteristic" OR discriminat* OR c-statistic OR "Area under the curve" OR AUC OR Calibration OR indices OR algorithm OR multivaria* OR Validat* OR Predict* OR Rule* OR Risk* OR Model* OR Criteria OR Scor* OR Characteristic* OR Finding* OR Factor* OR Decision* OR Prognos* OR Index OR model OR prevent*)) AND (title:(heal OR healing OR heals OR healed OR dress*) OR abstract:(heal OR healing OR heals OR healed OR dress*)) AND (title:(supine OR immobil*) OR abstract:(supine OR immobil*))
Limit by publication type: systematic review (39) (Update: 9) or broad synthesis (3) (Update: 0)

**GOOGLE SCHOLAR 1/02/23**

**Update run: 24/06/24**, limit to years 2023-2024

allintitle: prevent OR prevention OR risk OR predict OR prevents OR risks OR prediction OR predicts OR prognosis OR prognostic "pressure injury" -ulcer -ulcers Limit to review and years 2013-2023 (66)

(Update: 31)

allintitle: prevent OR prevention OR risk OR predict OR prevents OR risks OR prediction OR predicts OR prognosis OR prognostic "pressure injuries" -ulcer -ulcers Limit to review and years 2013-2023 (33)

(Update: 14)

allintitle: prevent OR prevention OR risk OR predict OR prevents OR risks OR prediction OR predicts OR prognosis OR prognostic "pressure ulcer” Limit to review and years 2013-2023 (120)

(Update: 14)

allintitle: prevent OR prevention OR risk OR predict OR prevents OR risks OR prediction OR predicts OR prognosis OR prognostic "pressure ulcers” Limit to review and years 2013-2023 (102)

(Update: 14)

allintitle: prevent OR prevention OR risk OR predict OR prevents OR risks OR prediction OR predicts OR prognosis OR prognostic "pressure sore” Limit to review and years 2013-2023 (3)

(Update: 0)

allintitle: prevent OR prevention OR risk OR predict OR prevents OR risks OR prediction OR predicts OR prognosis OR prognostic "pressure sores” Limit to review and years 2013-2023 (2)

(Update: 0)

allintitle: prevent OR prevention OR risk OR predict OR prevents OR risks OR prediction OR predicts OR prognosis OR prognostic "pressure wound” Limit to review and years 2013-2023 (25)

(Update: 4)

allintitle: prevent OR prevention OR risk OR predict OR prevents OR risks OR prediction OR predicts OR prognosis OR prognostic "pressure wounds” Limit to review and years 2013-2023 (0)

(Update: 0)

allintitle: prevent OR prevention OR risk OR predict OR prevents OR risks OR prediction OR predicts OR prognosis OR prognostic "bedsore” Limit to review and years 2013-2023 (1)

(Update: 0)

allintitle: prevent OR prevention OR risk OR predict OR prevents OR risks OR prediction OR predicts OR prognosis OR prognostic "bedsores” Limit to review and years 2013-2023 (1)

(Update: 1)

allintitle: prevent OR prevention OR risk OR predict OR prevents OR risks OR prediction OR predicts OR prognosis OR prognostic "bed sore” Limit to review and years 2013-2023 (0)

(Update: 0)

allintitle: prevent OR prevention OR risk OR predict OR prevents OR risks OR prediction OR predicts OR prognosis OR prognostic "bed sores” Limit to review and years 2013-2023 (0)

(Update: 0)

allintitle: prevent OR prevention OR risk OR predict OR prevents OR risks OR prediction OR predicts OR prognosis OR prognostic "decubitus” Limit to review and years 2013-2023 (4)

(Update: 2)

## Appendix 3: Data extraction form

| **Data Extraction Items** | | | |
| --- | --- | --- | --- |
|  | Extractor | | |
| **Publication information:** | Review Title;  First Author;  Publication Year;  Umbrella review eligibility (D/V, ACC, CE);  Comments;  Primary studies fundings reported?^A^;  Conflicts of Interest reported?^A^ | | |
| **Eligibility Criteria:** | Population;  Setting;  Prediction models/tools;  Model outcome (and classification if specified);  Interventions^B^;  Comparators^B^;  Outcomes of interest^B^;  Inclusion criteria incorporated PICO, PIRT or POII?^A^;  Source of data (prospective/retrospective);  Phase of development of models;  Study design;  Did they explain reasons for study design inclusions?^A^;  Exclusion criteria | | |
| **Review methods:** | Review protocol;  Protocol and justifications for deviations from?^A^;  Databases searched;  Adequate search strategy?^A^;  Search cut-off date;  Publication restrictions;  Quality assessment tool;  Suitable quality assessment tool?;  Study selection method;  Study selection in duplicate?^A^;  Quality assessment method;  Data extraction method;  Data extraction in duplicate?^A^;  Synthesis method;  Appropriate method of statistical synthesis, if applicable?^A^ | | |
| **Review results:** | PRISMA diagram provided?;  Excluded studies list (with justifications)?^A^;  N models per review;  N studies per review;  N participants in review;  How were the results presented? (e.g. outcomes reported);  Description of included studies provided? (summary table, tabulated per study, narrative only);  Description of included studies adequate?^A^;  Study quality described? (summary table, tabulated per study, narrative only);  Assessment of RoB satisfactory?^A^;  Assessment of impact of RoB on synthesised results?^A^;  Assessment of impact of RoB on review results?^A^;  Discussion/investigation of heterogeneity?^A^;  Models included;  Brief description of included studies;  Brief description of study quality | | |
| **D/V Reviews**  **(Re: prognostic studies)** | | **Accuracy Reviews**  **(Re: accuracy studies)** | **Clinical Effectiveness Reviews**  **(Re: effectiveness studies)** |
|  | | 2x2 tables presented for each study? |  |
|  | | Cut-off points specified for each study? |  |
|  | | List Author, year of primary studies included in review |  |
| Summary estimates:  Overall model performance (e.g. R-squared, Brier score)  Model calibration (e.g. calibration plot, slope, intercept, O/E ratio)  Model discrimination (e.g. c-statistic, AUC)  (results from statistical synthesis) | | Summary estimates:  Sensitivity (incl. n), specificity (incl. N), likelihood ratios, DOR, AUROC, predictive values  Summary Sensitivity (incl. n)  (results from statistical synthesis) | Summary of statistical synthesis of results  (e.g. effect on incidence of PI, treatment outcome, or other patient-relevant outcomes) |
| Summary of narrative synthesis of results | | Summary of narrative synthesis of results | Summary of narrative synthesis of results |
| AUC – area under the curve; AUROC – area under the receiver operating characteristic curve; AMSTAR – A MeaSurement Tool to Assess systematic Reviews; CE – clinical effectiveness; DOR – diagnostic odds ratio; ACC – test accuracy; D/V – development/validation; O/E – observed/expected; PI – pressure injury; PICO – population, intervention, comparator, outcome; PIRT – population, index test, reference standard, target condition; POII – population, outcome, intended use, intended timing; PRISMA – Preferred Reporting Items for Systematic Reviews and Meta-Analyses; RoB – risk of bias.  ^A^ AMSTAR-2 Items.  ^B^ applicable to clinical effectiveness reviews only. | | | |

## Appendix 4: AMSTAR-2 Methodology Quality Appraisal. Adapted for application to reviews of prognostic model and accuracy studies.

|  | AMSTAR-2 Adapted | |
| --- | --- | --- |
|  | Questions | Guidance |
| Item 1. | 1. Did the research questions and inclusion criteria for the review include the components one of the following: PICO, PIRT, or POII? **Y/N** | For intervention reviews: Population, Intervention, Comparator, Outcome  For prognostic accuracy reviews: Population, Index test, Reference standard, Target condition (PIRT) Population, Outcome to be predicted, Intended use of model, Intended moment in time (POII) |
| **Item 2*** | 2. Did the report of the review contain an explicit statement that the review methods were established prior to the conduct of the review and did the report justify any significant deviations from the protocol? **Y/PY/N** | For Partial Yes (PY): The authors state that they had a written protocol or guide that included ALL the following:   - review question(s), - a search strategy, - inclusion/exclusion criteria, - a risk of bias assessment.   For Yes: As for partial yes, plus the protocol should be registered and should also have specified:   - a meta-analysis/synthesis plan, if appropriate, - and a plan for investigating causes of heterogeneity, - justification for any deviations from the protocol. |
| Item 3. | 3. Did the review authors explain their selection of the study designs for inclusion in the review? **Y/N** | For Yes, the review should give an explanation for including types of studies included in the review, for example:  For the development/validation review: development studies, validation studies or both.  For the accuracy/effectiveness review: single group (prospective/retrospective), two/multi group (i.e. diagnostic case-control), RCTs, NSRs |
| **Item 4*** | 4. Did the review authors use a comprehensive literature search strategy? **Y/PY/N** | For Partial Yes (all the following):   - searched at least 2 databases (relevant to research question), - provided key word and/or search strategy, - justified publication restrictions (e.g. language).   For Yes, should also have (all the following):   - searched the reference lists / bibliographies of included studies, - searched trial/study registries, - included/consulted content experts in the field where relevant, - searched for grey literature, - conducted search within 24 months of completion of the review. |
| Item 5. | 5. Did the review authors perform study selection in duplicate? **Y/N** | For Yes, either ONE of the following: at least two reviewers independently agreed on selection of eligible studies and achieved consensus on which studies to include, OR two reviewers selected a sample of eligible studies and achieved good agreement (at least 80 percent), with the remainder selected by one reviewer. |
| Item 6. | 6. Did the review authors perform data extraction in duplicate? **Y/N** | For Yes, either ONE of the following: at least two reviewers achieved consensus on which data to extract from included studies, OR two reviewers extracted data from a sample of eligible studies and achieved good agreement (at least 80 percent), with the remainder extracted by one reviewer. |
| **Item 7*** | 7. Did the review authors provide a list of excluded studies and justify the exclusions? **Y/PY/N** | For Partial Yes: provided a list of all potentially relevant studies that were read in full-text form but excluded from the review  For Yes, must also have: Justified the exclusion from the review of each potentially relevant study |
| Item 8. | 8. Did the review authors describe the included studies in adequate detail? **Y/PY/N** | For Partial Yes (ALL the following per included study):   - described PICO/PIRT/POII (whichever applicable), - and described research designs   For Yes, should also have ALL the following per included study:   - described PICO/PIRT/POII (whichever applicable) in detail, - described study’s setting - and timeframe for follow-up |
| **Item 9*** | 9. Did the review authors use a satisfactory technique for assessing the risk of bias (RoB) in  individual studies that were included in the review? **Y/PY/N** | **RCTs** For Partial Yes, must have reported summary findings and assessed RoB from:   - unconcealed allocation, - and lack of blinding of patients and assessors when assessing outcomes (unnecessary for objective outcomes such as all-cause mortality)   For Yes, must also have given itemisation of quality judgements per study, and assessed RoB from:   - allocation sequence that was not truly random, - and selection of the reported result from among multiple measurements or analyses of a specified outcome |
|  |  | **NRS** For Partial Yes, must have reported summary findings and assessed RoB from:   - confounding, - and from selection bias   For Yes, must also have given itemisation of quality judgements per study, and assessed RoB from:   - methods used to ascertain exposures and outcomes, - and selection of the reported result from among multiple measurements or analyses of a specified outcome |
|  |  | **Accuracy studies** For Partial Yes, must have assessed RoB with a recognised tool (e.g. QUADAS-2) and given summary of result across domains  For Yes, must also have also given itemisation of quality judgements per study. |
|  |  | **Prognostic studies** For Partial Yes, must have assessed RoB with a recognised tool (e.g. PROBAST, QUIPS) and given summary of result across domains  For Yes, must also have also given itemisation of quality judgements per study. |
| Item 10. | 10. Did the review authors report on the sources of funding for the studies included in the review? **Y/N** | For Yes: Must have reported on the sources of funding for individual studies included in the review. Note: Reporting that the reviewers looked for this information, but it was not reported by study authors also qualifies |
| **Item 11*** | 11. If meta-analysis was performed did the review authors use appropriate methods for statistical combination of results? **Y/N/ 'No MA conducted'** | For Yes: The authors justified combining the data in a meta-analysis AND they used an appropriate weighted technique to combine study results and adjusted for heterogeneity if present. AND investigated the causes of any heterogeneity |
| Item 12. | 12. If meta-analysis was performed, did the review authors assess the potential impact of RoB in individual studies on the results of the meta-analysis or other evidence synthesis? **Y/N/ 'No MA conducted'** | For Yes: included only low risk of bias studies OR, if the pooled estimate was based on studies at variable RoB, the authors performed sensitivity analyses to investigate possible impact of RoB on summary estimates |
| **Item 13*** | 13. Did the review authors account for RoB in individual studies when interpreting/ discussing the results of the review? **Y/N** | For Yes: included only low risk of bias RCTs OR, if RCTs with moderate or high RoB, or NRSs were included the review provided a discussion of the likely impact of RoB on the results |
| Item 14. | 14. Did the review authors provide a satisfactory explanation for, and discussion of, any heterogeneity observed in the results of the review? **Y/N** | For Yes: There was no significant heterogeneity, OR if heterogeneity was present, the authors performed an investigation of main sources of heterogeneity in the results, if applicable, and particularly any between-study heterogeneity and discussed the impact of this |
| Item 15. | 15. Did the review authors report any potential sources of conflict of interest, including any funding they received for conducting the review? **Y/N** | For Yes: The authors reported no competing interests, OR the authors described their funding sources and how they managed potential conflicts of interest |
|  | * Critical domains identified by AMSTAR-2 developers.^1^  DEV/VAL – development/validation; MA – Meta-Analysis; N – No; NRS – non-randomised study; PICO – population, intervention, comparator, outcome; PIRT – population, index test, reference standard, target condition; POII – population, outcome, intended use, intended time; PROBAST – Prediction model Risk Of Bias ASsessment Tool; PY – Partial Yes; QUADAS – Quality Assessment of Diagnostic Accuracy Studies; QUIPS – Quality In Prognosis Studies; RCT – randomised controlled trial; RoB – risk of bias; Y – Yes. | |

## Appendix 5: Detailed results tables

### Table S1. Full-text articles excluded, with reasons

|  | **Author, year** | **Title** | **Major reason for exclusion** |
| --- | --- | --- | --- |
| 1 | Alves, 2014 ^2^ | *Assessment of risk for pressure ulcers in intensive care units: an integrative review* | Not a systematic review |
| 2 | Anthony, 2008 ^3^ | *Norton, Waterlow and Braden scores: a review of the literature and a comparison between the scores and clinical judgement* | Not a systematic review |
| 3 | Barradas Cavalcante, 2016 ^4^ | *Updating pf the assistance protocol for pressure prevention: evidence based practice* | Not a systematic review |
| 4 | Charalambous, 2018 ^5^ | *Evaluation of the Validity and Reliability of the Waterlow Pressure Ulcer Risk Assessment Scale* | Not a systematic review |
| 5 | de Laat, 2006 ^6^ | *Epidemiology, risk and prevention of pressure ulcers in critically ill patients: a literature review* | Not a systematic review |
| 6 | do Egito Cavalcanti de Farias, 2022 ^7^ | *Risk factors for the development of pressure injury in the elderly: integrative review* | Not a systematic review |
| 7 | Feuchtinger, 2005 ^8^ | *Pressure ulcer risk factors in cardiac surgery: A review of the research literature* | Not a systematic review |
| 8 | Garcia-Fernandez, 2014 ^9^ | *A new theoretical model for the development of pressure ulcers and other dependence-related lesions* | Not a systematic review |
| 9 | Garrubba, 2017 ^10^ | *Effectiveness of the Braden risk screening tool for pressure injuries: systematic review* | Not a systematic review |
| 10 | Kelechi, 2013 ^11^ | *Review of pressure ulcer risk assessment scales* | Not a systematic review |
| 11 | Keller, 2002 ^12^ | *Pressure ulcers in intensive care patients: A review of risks and prevention* | Not a systematic review |
| 12 | Ladd, 2018 ^13^ | *A systematic review of pressure ulcers in burn patients: Risk factors, demographics, and treatment modalities* | Not a systematic review |
| 13 | Lepisto, 2006 ^14^ | *Developing a Pressure Ulcer Risk Assessment Scale for Patients in Long-Term Care* | Not a systematic review |
| 14 | Mendes Coqueiro, 2013 ^15^ | *Multiple risk factors and preventive strategies of pressure ulcers: systematic review* | Not a systematic review |
| 15 | Michel, 2012 ^16^ | *As of 2012, what are the key predictive risk factors for pressure ulcers? Developing French guidelines for clinical practice* | Not a systematic review |
| 16 | Ming, 2012 ^17^ | *Systematic review of pressure ulcer risk assessment scales for using in ICU patients* | Not a systematic review |
| 17 | Mordiffi, 2010 ^18^ | *Evaluating the effects of using the mobility assessment sub-scale within the Braden Scale on pressure ulcer incidence and preventive interventions in adult acute care settings: A systematic review* | Not a systematic review |
| 18 | Mortenson, 2008 ^19^ | *A review of scales for assessing the risk of developing a pressure ulcer in individuals with SCI* | Not a systematic review |
| 19 | Nadeem, 2021 ^20^ | *Utility of the Waterlow scale in acute care settings: A literature review* | Not a systematic review |
| 20 | O’Tuathail, 2011 ^21^ | *Evaluation of three commonly used pressure ulcer risk assessment scales* | Not a systematic review |
| 21 | Rodriguez Torres, 2007 ^22^ | *Clinical judgement or assessment scales to identify patients at risk of developing pressure ulcers?* | Not a systematic review |
| 22 | Sales de Almeida, 2020 ^23^ | *Pressure injury prevention scales in intensive care units: an integrative review* | Not a systematic review |
| 23 | Santos, 2015 ^24^ | *Development of the nursing diagnosis risk for pressure ulcer* | Not a systematic review |
| 24 | Satekova, 2014 ^25^ | *Validity of pressure ulcer risk assesment scales: Review* | Not a systematic review |
| 25 | Shahin, 2007 ^26^ | *Predictive validity of pressure ulcer risk assessment tools in intensive care patients* | Not a systematic review |
| 26 | Smet, 2019 ^27^ | *The Belgian pressure ulcer risk assessment project: Is assessing mobility and skin status a more accurate, reliable, and feasible approach to assess pressure ulcer risk in hospitalised patients?* | Not a systematic review |
| 27 | Solati, 2016 ^28^ | *Predictive values of Braden and Waterlow scales to assess the risk of pressure ulcer* | Not a systematic review |
| 28 | Taylor, 1988 ^29^ | *Assessment tools for the identification of patients at risk for the development of pressure sores: a review* | Not a systematic review |
| 29 | Tran, 2016 ^30^ | *Prevention of Pressure Ulcers in the Acute Care Setting: New Innovations and Technologies* | Not a systematic review |
| 30 | Tschannen, 2020 ^31^ | *The pressure injury predictive model: A framework for hospital-acquired pressure injuries* | Not a systematic review |
| 31 | Walsh, 2011 ^32^ | *Investigating the reliability and validity of the Waterlow risk assessment scale: A literature review* | Not a systematic review |
| 32 | Xu, 2018 ^33^ | *Risk assessment tools for pressure injury in intensive care patients: a review* | Not a systematic review |
| 33 | Alderden, 2017 ^34^ | *Risk factors for pressure injuries among critical care patients: A systematic review* | No risk prediction models |
| 34 | Barbosa da Silva, 2020 ^35^ | *Pressure ulcers in individuals with spinal cord injury: risk factors in neurological rehabilitation* | No risk prediction models |
| 35 | Di Prinzio, 2019 ^36^ | *Risk factors for the development and recurrence of pressure ulcers in patients with spinal cord injury: A systematic review* | No risk prediction models |
| 36 | Haisley, 2020 ^37^ | *Postoperative pressure injuries in adults having surgery under general anaesthesia: systematic review of perioperative risk factors* | No risk prediction models |
| 37 | Ham, 2014 ^38^ | *Pressure ulcers from spinal immobilization in trauma patients: A systematic review* | No risk prediction models |
| 38 | Lima, 2021 ^39^ | *Risk factors and preventive interventions for pressure injuries in cancer patients* | No risk prediction models |
| 39 | Lima Serrano, 2017 ^40^ | *Risk factors for pressure ulcer development in Intensive Care Units: Systematic review* | No risk prediction models |
| 40 | Marin, 2013 ^41^ | *A systematic review of risk factors for the development and recurrence of pressure ulcers in people with spinal cord injuries* | No risk prediction models |
| 41 | Rao, 2016 ^42^ | *Risk Factors Associated With Pressure Ulcer Formation in Critically Ill Cardiac Surgery Patients: A Systematic Review* | No risk prediction models |
| 42 | Reenalda, 2009 ^43^ | *Clinical use of interface pressure to predict pressure ulcer development: a systematic review* | No risk prediction models |
| 43 | Shi, 2018 ^44^ | *Skin status for predicting pressure ulcer development: A systematic review and meta-analyses* | No risk prediction models |
| 44 | Siping, 2022 ^45^ | *Risk factors of intraoperative acquired pressure injury: A systematic review and meta-analysis* | No risk prediction models |
| 45 | Wynn, 2022 ^46^ | *Risk factors for the development and evolution of deep tissue injuries: A systematic review* | No risk prediction models |
| 46 | Zhang, 2022 ^47^ | *Prevalence and Risk Factors of Postoperative Pressure Ulcers: A Systematic Review and Meta-analysis of Diagnostic Test* | No risk prediction models |
| 47 | Bulfone, 2018 ^48^ | *Perioperative Pressure Injuries: A Systematic Literature Review* | Wrong research question |
| 48 | Chung, 2022 ^49^ | *Risk Factors for Pressure Injuries in Adult Patients: A Narrative Synthesis* | Wrong research question |
| 49 | Chung, 2022 ^50^ | *Risk factors for pressure ulcers in adult patients: A meta-analysis on sociodemographic factors and the Braden scale* | Wrong research question |
| 50 | Coleman, 2013 ^51^ | *Patient risk factors for pressure ulcer development: Systematic review* | Wrong research question |
| 51 | Dube, 2022 ^52^ | *Risk factors associated with heel pressure ulcer development in adult population: A systematic literature review* | Wrong research question |
| 52 | Ferris, 2019 ^53^ | *Pressure ulcers in patients receiving palliative care: A systematic review* | Wrong research question |
| 53 | Floyd, 2018 ^54^ | *Effectiveness of pressure ulcer protocols with the Braden Scale for elderly patients in the intensive care unit: A Systematic Review* | Wrong research question |
| 54 | Gelis, 2009 ^55^ | *Pressure ulcer risk factors in persons with SCI: Part I: Acute and rehabilitation stages* | Wrong research question |
| 55 | Gelis, 2009 ^56^ | *Pressure ulcer risk factors in persons with spinal cord injury part 2: the chronic stage* | Wrong research question |
| 56 | Liu, 2024 ^57^ | *Effects of predictive nursing interventions on pressure*  *ulcer in older bedridden patients: A meta-analysis* | Wrong research question |
| 57 | Moore, 2023 ^58^ | *A systematic review of movement monitoring devices to aid the prediction of pressure ulcers in at-risk adults* | Wrong research question |
| 58 | Mordiffi, 2011 ^59^ | *Use of mobility subscale for risk assessment of pressure ulcer incidence and preventive interventions: A systematic review* | Wrong research question |
| 59 | Nixon, 2015 ^60^ | *Pressure UlceR Programme Of reSEarch (PURPOSE): using mixed methods (systematic reviews, prospective cohort, case study, consensus and psychometrics) to identify patient and organisational risk, develop a risk assessment tool and patient-reported outcome Quality of Life and Health Utility measures* | Wrong research question |
| 60 | Richardson, 2015 ^61^ | *Part 1: Pressure ulcer assessment - the development of Critical Care Pressure Ulcer Assessment Tool made Easy (CALCULATE)* | Wrong research question |
| 61 | Teixeira, 2022 ^62^ | *Risk factors for pressure injury in critically ill polytraumatized patients: A systematic review* | Wrong research question |
| 62 | Ting, 2021 ^63^ | *E-Health Decision Support Technologies in the Prevention and Management of Pressure Ulcers: A Systematic Review* | Wrong research question |
| 63 | Toffaha, 2023 ^64^ | *Leveraging artificial intelligence and decision support systems in hospital-acquired pressure injuries prediction: A comprehensive review* | Wrong research question |
| 64 | Fuentelsaz Gallego, 2005 ^65^ | *Review of literature on pressure ulcers in people aged 65 or over* | No English language translation |
| 65 | Garcia-Fernandez, 2013 ^66^ | *Risk assessment scales for pressure ulcer in intensive care units: A systematic review with metaanalysis* | No English language translation |
| 66 | Kottner, 2008 ^67^ | *Interrater reliability of the Braden scale* | No English language translation |
| 67 | Nunes de Sousa, 2023 ^68^ | *SCALES USED TO MEASURE PRESSURE INJURY RISK IN HOSPITALIZED PATIENTS: A REVIEW* | No English language translation |
| 68 | Pancorbo-Hidalgo, 2008 ^69^ | *Pressure ulcers risk assessment: clinical practice in Spain and a meta-analysis of scales effectiveness* | No English language translation |
| 69 | Park, 2014 ^70^ | *Predictive validity of the Braden Scale for pressure ulcer risk: a meta-analysis* | No English language translation |
| 70 | Yang, 2019 ^71^ | *Predictive validity of the Munro Scale for pressure injuries in surgical patients: A meta-analysis* | No English language translation |
| 71 | De Queiroz, 2022 ^7^ | *Risk factors for the development of pressure injury in the elderly: integrative review/Fatores de risco o para desenvolvimento de lesão por pressão em idosos: revisão integrativa* | Duplicate |
| 72 | Garcia-Fernandez, 2013 ^66^ | *Risk assessment scales for pressure ulcers in intensive care units: A systematic review with meta-analysis* | Duplicate |
| 73 | Nixon, 2015 ^60^ | *Pressure UlceR Programme Of reSEarch (PURPOSE): using mixed methods (systematic reviews, prospective cohort, case study, consensus and psychometrics) to identify patient and organisational risk, develop a risk assessment tool and patient-reported outcome Quality of Life and Health Utility measures* | Duplicate |
| 74 | Nayar, 2021 ^72^ | *Waterlow score for risk assessment in surgical patients: a systematic review* | Wrong outcome |
| 75 | Zahia, 2020 ^73^ | *Pressure injury image analysis with machine learning techniques: A systematic review on previous and possible future methods* | Wrong outcome |
| 76 | Moore, 2008 ^74^ | *Risk assessment tools for the prevention of pressure ulcers* | Updated version included |
| 77 | Moore, 2014 ^75^ | *Risk assessment tools for the prevention of pressure ulcers* | Updated version included |
| 78 | Liao, 2018 ^76^ | *Predictive accuracy of the Braden Q Scale in risk assessment for paediatric pressure ulcer: A meta-analysis* | Wrong population |
| 79 | Ribeiro, 2013 ^77^ | *How effective is the development of skin care in critically ill patients using the Braden Scale scores aiming to prevent the incidence of pressure ulcers? Sistematic Literature Review* | No results |

### Table S2. Systematic review characteristics

| **Review author**  (publication year)  **Review question** | **Eligibility criteria** | | | **Review methods** | | | | | **Volume of evidence** | |
| --- | --- | --- | --- | --- | --- | --- | --- | --- | --- | --- |
|  | **Population; setting** | **Prediction tools; PI classification system** | **Study design** | **Databases searched** | **Publication restrictions**  Year; language; publication type | **Quality assessment tool** | **Meta-analysis included; method of meta-analysis** | **N relevant studies in review**  (n participants) | | **N tools included** |
| Barghouthi^78^ (2023)  Model development | “Adult” inpatients (age ≥14y); hospital | ML; NS | NS | CINAHL; PubMed; Science Direct; IEEE; Cochrane; Google Scholar | 2017-2023; English; NS | JBI appraisal checklist for cohort studies | No | 23 (706393) | | 23 |
| Baris^79^ (2015)  Effectiveness | Turkish populations only; NS | Braden; NS | NS | Turkish MEDLINE; PubMed; ScienceDirect; Google Scholar; YOK Thesis Search; Reference Directory of Turkey; Medicine Directory of Turkish Clinics; ULAKBIM National Database; National Library Bibliography of Turkish Articles | 1998-2012; English, Turkish; NS | None | No | 16 (2273^a^) | | 2 |
| Chen^80^ (2023)  Accuracy | Patients with a critical illness; ICU | Cubbin & Jackson; NS | Diagnostic studies (presenting TP, FP, TN and FN results) with any research design | EBSCO; PubMed; Ovid; Web of Science; Cochrane databases; Wangfang Data; China National  Knowledge Infrastructure | Database inception - 2021; NS; reviews and expert opinions excluded | QUADAS-II | Yes; meta-analysis method unclear, SROC analysis | 9 (7684) | | 1 |
| Chen^81^ (2016)  Accuracy | NS; long-term care | Braden; NS | NS | PubMed; Web of Science | Inception-2015; English; NS | QUADAS | Yes; DerSimonian and Laird random-effects model, SROC analysis | 8 (41489) | | 1 |
| Chou^82^ (2013)  Accuracy  Effectiveness | Adults (age ≥18y); acute care hospital, long-term and rehabilitation facilities, operative and postoperative, community (home care and wheelchair users) | PI risk assessment tools; NS | KQ1^b^: controlled or comparative randomised and nonrandomised trials, controlled or comparative observational studies KQ2^b^: prospective studies of predictive validity (case-control excluded) | MEDLINE; CINAHL; Cochrane Library; grant databases; clinical trial registries | 1946-2021 (MEDLINE), 1988-2012 (CINAHL), inception- 2012 (Cochrane library); English; conference abstracts excluded | Criteria consistent with AHRQ Methods Guide for Effectiveness and Comparative Effectiveness Reviews | No; presented median accuracy results | KQ1^b^: 3 KQ2^b^: 47 | | KQ1^b^: 4 KQ2^b^: 20 |
| Dweekat^83^ (2023)  Model development | NS; NS | ML; NS | NS | PubMed; Web of Science; Scopus; Science Direct | 2007-2022; English; article papers, review papers, conference proceedings | None | No | 35 (664719) | | 35 |
| Garcia-Fernandez^84^ (2014)  Accuracy | No PIs at baseline, no age restriction; NS | PI risk assessment tools; NS | Controlled clinical trials, prospective cohort | Cochrane Library; Center for Reviews and Dissemination University of York; LILACS; CUIDEN Plus; Spanish Medical Index | 1962-2010; no restriction; peer-reviewed journal article | CASP for RCT/cohort studies | Yes; random-effects model | 70 (30327) | | 28 |
| Gaspar^85^ (2019)  Effectiveness | Adult inpatients; hospital wards or any acute unit | PI prevention strategies; NS | Prospective or retrospective; cross-sectional, comparative, pre-test and post-test, quasi-experimental, experimental, RCT, mixed-method | MEDLINE; CINAHL; PubMed; Web of Science; EBSCO Nursing & Allied Health; Cochrane Central Register of Controlled Trials; Library, Information Science & Technology Abstracts; MedicLatina | 2009-2018; English, French, Portuguese, Spanish; peer-reviewed | Evidence-Based Librarianship Critical Appraisal checklist | No | 1 (1231) | | 2 |
| He^86^ (2012)  Accuracy | NS; surgical | Braden; NS | Studies assessing predictive validity | PubMed; Web of Science | Not stated-2011; NS; NS | QUADAS | Yes; DerSimonian and Laird random-effects model, SROC analysis | 3 (609) | | 1 |
| Health Quality Ontario^87^ (2009)  Effectiveness | Any population at risk of developing PIs; NS | PI risk assessment tools; NS | Systematic reviews, RCTs, non-randomised controlled clinical trials | MEDLINE; MEDLINE In-Process; CINAHL; EMBASE; Cochrane Library; other non-indexed citations | 1997-2008; English; NS | Criteria name not given | No | 3 (528) | | 3 |
| Huang^88^ (2021)  Accuracy | Inpatients aged ≥18y, no PIs at admission; NS | Braden; accepted standards (NPUAP, EPUAP, AHCPR, ICD-9, Bergstrom, others) | Cross-sectional, cohort | PubMed; CINAHL; EMBASE; Web of Science; Cochrane Library; bibliographies | Inception-2020; NS; NS | QUADAS-II | Yes; bivariate model, SROC analysis | 60 (49326) | | 1 |
| Jiang^89^ (2021)  Development | Any population; NS | ML; NS | NS | CINAHL; PubMed; EMBASE; Web of Science; Cochrane Library; China National Knowledge Infrastructure; Wanfang database; VIP database; China Biomedical Literature Database | NS-2020; English, Chinese; review papers, opinion papers, editorials, discussion papers, dissertations, conference abstracts excluded | PROBAST | No | 9 (1278148) | | 9 |
| Kottner^90^ (2009)  Effectiveness (reliability) | NS; NS | Waterlow; NS | Inter- and intrarater reliability and agreement | MEDLINE; EMBASE; CINAHL | 1985-2008; English, German; original research | Own criteria | No | 8 | | 2 |
| Lovegrove^91^ (2021)  Effectiveness | Adults (age ≥18y); acute hospital care | PI risk assessment tools; NS | Primary research | MEDLINE; EMBASE; EBSCO CINAHL; EBSCO; Scopus; Web of Science | 2010-2020; English; conference abstracts, posters excluded | JBI tools  or analytical cross-sectional study appraisal checklist | No | 5 (1910) | | 5 |
| Lovegrove^92^ (2018)  Effectiveness | Adults; hospital or acute care | PI risk assessment tools; NS | Primary research | MEDLINE; CINAHL; Scopus; Web of Science | 2007-2017; English; non-research publications excluded | JBI tools | No | 20 | | 5^b^ |
| Mehicic^93^ (2024)  Accuracy  Effectiveness (reliability, measurement error and convergent validity) | Adults (age ≥18y); ICU  For reliability assessment: sample of nurse-raters required | Braden; NS | Primary quantitative or mixed-methods research studies | CINAHL; EMBASE; MEDLINE; Scopus; Web of Science | Database inception - 2023; English language; Peer-reviewed | COSMIN RoB checklist | No | 34 (59325) | | 1 |
| Moore^94^ (2019)  Effectiveness | People without PIs, any age; any healthcare setting | PI risk assessment tools; validated PI staging system | RCTs or cluster-RCTs | MEDLINE; EMBASE; CINAHL; Cochrane Wounds Specialised Register; Cochrane Central Register of Controlled Trials | Start date between 1937-1974, until 2018; no restrictions; no restrictions | Cochrane RoB tool | No | 2 (1487) | | 3 |
| Pancorbo-Hidalgo^95^ (2006)  Accuracy  Effectiveness | No PIs at baseline; NS | PI risk assessment tools; NS | Controlled clinical trials, prospective cohort | MEDLINE; CINAHL; EBSCO; ScienceDirect; Current contents; DARE; Indice medico espanol; LILACS; CUIDEN; Cochrane Library; Springer; InterSciencia; ProQuest; Pascal | 1966-2003; Spanish, English, French, Portuguese; no restrictions | CASP Guide for clincial trials; critical assessment guide for PI assessment and prevention for cohort studies | Yes; weighted average values using inverse of variance for weights, DerSimonian and Laird random-effects model | 33 | | 13 |
| Park^96^ (2016a)  Accuracy | NS; NS | Modified Braden, Waterlow, Norton, Cubbin & Jackson; NPUAP, EPUAP, AHCPR, Torrence Developmental Classification of Pressure Sore | NS | MEDLINE; EMBASE; CINAHL; Cochrane Library; KoreaMed; NDSL; KERIS | NS-2013; NS; NS | QUADAS-II | Yes; random-effects model, SROC analysis | 17 (6143) | | 5 |
| Park^97^ (2016b)  Accuracy | Elderly (age ≥60y); NS | Braden, Waterlow, Norton; NS | NS | MEDLINE; EMBASE; CINAHL; Cochrane database; KoreaMed | 1966-2013; NS; NS | QUADAS-II | Yes; random-effects model, SROC analysis | 29 (11729) | | 3 |
| Park^98^ (2015)  Accuracy | Adults (age ≥18y) with no PIs at baseline; hospitalised | Braden; NPUAP, AHCPR, others | Prospective | MEDLINE; EMBASE; CINAHL; KoreaMed;  Cochrane Library; National Digital Science Library; Korea Education and Research Information Service | NS-2013; NS; NS | QUADAS-II | Yes; random-effects model, SROC analysis | 21 (6070) | | 1 |
| Pei^99^ (2023)  Model development & validation  Accuracy | Adult; hospital | ML (if >1 model per study, only the *‘best’* was included); NS | NS | PubMed; Embase; Cochrane Library; Web of Science; CINAHL; Grey literature; and “other databases” | Database inception - 2022; English and Chinese; peer-reviewed articles or full-length conference proceedings | PROBAST | Yes; random-effects model, SROC analysis | 18 (408504) | | 18 |
| Qu^100^ (2022)  Accuracy | Adults with no PIs at baseline; hospital inpatients | ML; Munoz and Posthauer (2021) PI stage or as defined by the study authors | Diagnostic trials, crossover trials, cluster-controlled trials | MEDLINE; EMBASE; EBSCO; Web of Science | Start date between 1985-2010, until 2021; English; NS | QUADAS-II; PROBAST | Yes; fixed-effects or random-effects model dependent on heterogeneity assessment, ANOVA model for Bayesian network meta-analysis for diagnostic test accuracy | 24 (221541) | | 24 |
| Ribeiro^101^ (2021)  Model development | Bedridden patients (at risk of PI); NS | ML; NS | NS | Scopus; Web of Science | 2010-2021; English; review papers, opinion paper, extended abstracts excluded | None | No | 3 (6674) | | 3 |
| Shi^102^ (2019)  Model development & validation | Any; NS | Empirically derived multivariable models, including ML models; NS | Objectives 1 & 2: prospective or retrospective longitudinal  Objective 3: RCTs, non-randomised trials, prospective or retrospective 'before-and-after' | MEDLINE; CINAHL; ProQuest | 1946-2017 (MEDLINE), 1937-2017 (CINAHL), inception-2017 (ProQuest); no restrictions; no restrictions | PROBAST | Yes; fixed-effects or random-effects model dependent on heterogeneity assessment | 23 (72326) | | 21 |
| Tayyib^103^ (2013)  Accuracy  Effectiveness | Adults; ICU | NS; NPUAP/EPUAP | Quantitative | MEDLINE; PubMed; CINHAL; EBSCOHost; Cochrane Library; ProQuest; Google Scholar | 2000-2012; English; journals, books, handbooks, abstracts | None | No | 11 (2119) | | 9 |
| Wang^104^ (2022)  Accuracy | Any age; any healthcare setting | NS; NS | Primary research and sample size, except case reports or case series | PubMed; EMBASE; CINAHL; Cochrane Library | Inception-2021; English; NS | JBI tools; NOS | Yes; fixed-effects or random-effects model dependent on heterogeneity assessment | 2 (992) | | 2 |
| Wei^105^ (2020)  Accuracy | Adults (age >18y); ICU | Braden; NS | NS | PubMed; Web of Science; Cochrane Library; SinoMed; CNKI; Wanfang | NS-2019; no restrictions; NS | QUADAS-II | Yes; DerSimonian and Laird random-efects model | 11 (10044) | | 1 |
| Wilchesky^106^ (2015)  Accuracy | NS; long-term care | Braden; NS | NS | MEDLINE; PubMed; EMBASE; PsychINFO | 1985-2013; English; journal articles (reviews and opinion papers excluded) | None | Yes; DerSimonian and Laird random-effects model | 9 (40361) | | 1 |
| Zhang^107^ (2021)  Accuracy | Inpatient aged >18y; ICU (stay >24h) | PI risk assessment tools; standard for judging the occurrence of PI had to be described | Cohort, case-control | PubMed/MEDLINE; EMBASE; CINAHL; Web of Science; Cochrane Library; China Biomedical Literature Service System; VIP Database; CNKI | Inception-2019; no restrictions; NS | QUADAS-II | Yes; hierarchal SROC model | 23 (15199) | | 15 |
| Zhou^108^ (2022)  Model development | Any inpatient; hospital | ML; NS | NS | PubMed; EMBASE; CINHAL; Web of Science; Scopus | 2010-2021; English; NS | PROBAST | No | 22 (234105) | | 22 |
| Zimmerman^109^ (2018)  Accuracy | Adult inpatients; ICU | Any scale or index; NS | NS | MEDLINE; CINAHL COCHRANE; El Banco de Datos de Enfermería; nursing database; LILACS | 1962-2016; English, Portuguese, Spanish; NS | None | No | 13 | | 11 |

AHRQ – Agency for Healthcare Research and Quality; AHCPR – Agency for Health Care Policy and Research; CASP – Critical Appraisal Skills Programme; CNKI – China National Knowledge Infrastructure; CUIDEN – Bibliographic Database Index Foundation including scientific production on Health Care in Latin American; DARE – Database of Abstracts of Reviews of Effects; DEV – model development study; EPUAP – European Pressure Ulcer Advisory Panel; HCW – health care worker; ICU – intensive care unit; ICD-9 – International Classification of Diseases Ninth Edition; IEEE – Institute of Electrical and Electronics Engineers; JBI – Joanna Briggs Institute; LILACS – Latin America and Caribbean Health Sciences Literature; ML – machine learning; NOS – Newcastle Ottowa Scale; NPUAP – National Pressure Ulcer Advisory Panel; NS – not stated; PI – pressure injury; PROBAST – Prediction model Risk of Bias Assessment; QUADAS – Quality Assessment of Diagnostic Accuracy Studies; SROC – summary receiver operating curve; ULAKBIM – Turkish Academic Network and Information Center; VAL – model validation study.

^a^Patients and HCWs

^b^KQ1 – key question 1 looks at effectiveness of risk assessment tools; KQ2 – key question 2 looks at diagnostic accuracy/validity of risk assessment tools

^b^Version of modified Norton scale cannot be determine.

### Table S3. AMSTAR-2 assessment results per review

| **Review author**  (pub. year) | **ITEM 1** | **ITEM 2** | **ITEM 3** | **ITEM 4** | **ITEM 5** | **ITEM 6** | **ITEM 7** | **ITEM 8** | **ITEM 9** | **ITEM 10** | **ITEM 11** | **ITEM 12** | **ITEM 13** | **ITEM 14** | **ITEM 15** | **Overall confidence** |
| --- | --- | --- | --- | --- | --- | --- | --- | --- | --- | --- | --- | --- | --- | --- | --- | --- |
| **Model development and validation reviews** | | | | | | | | | | | | | | | | |
| Barghouthi^78^  (2023) | N | N | N | N | Y | N | N | N | N | N | N/A | N/A | N | N | Y | **Critically Low** Y=2/13  PY=0/13  N=11/13 |
| Dweekat^83^ (2023) | N | N | N | PY | Y | N | N | N | N | N | N/A | N/A | N | N | Y | **Critically Low***  Y=2/13  PY=1/13  N=10/13 |
| Jiang^89^  (2021) | N | N | N | PY | Y | N | N | N | PY | N | N/A | N/A | N | N | Y | **Critically Low** Y=2/13  PY=2/13  N=9/13 |
| Pei^99^  (2023) | N | Y | N | Y | N | Y | N | N | Y | N | N | N | Y | Y | Y | **Critically Low**  Y=7/15  PY=0/15  N=8/15 |
| Ribeiro^101^ (2021) | N | N | N | PY | N | Y | N | N | N | N | N/A | N/A | N | N | Y | **Critically Low** Y=2/13  PY=1/13  N=10/13 |
| Shi^102^  (2019) | Y | Y | Y | PY | Y | N | N | Y | Y | N | N | Y | Y | Y | Y | **Low**  Y=10/15  PY=1/15  N=4/15 |
| Zhou^108^  (2022) | N | N | N | PY | Y | Y | N | N | Y | N | N/A | N/A | N | N | Y | **Critically Low** Y=4/13  PY=2/13  N=8/13 |
| **Summary** | **1/7 Yes** | **2/7 Yes** | **1/7 Yes** | **1/7 Yes**  **6/7 PY** | **5/7 Yes** | **3/7 Yes** | **0/7 Yes** | **1/7 Yes** | **3/7 Yes**  **1/7 PY** | **0/7 Yes** | **0/2 Yes** | **1/2 Yes** | **2/7 Yes** | **2/7 Yes** | **7/7 Yes** |  |
|  |  |  |  |  |  |  |  |  |  |  |  |  |  |  |  |  |
|  | | | | | | | | | | | | | | | | |
| **Prognostic accuracy reviews** | | | | | | | | | | | | | | | | |
| Chen^80^  (2023) | N | N | N | PY | Y | Y | N | PY | Y | N | N | N | N | N | Y | **Critically Low**  Y=3/15  PY=2/15  N=10/15 |
| Chen^81^  (2016) | N | N | N | PY | Y | N | N | PY | PY | N | N | N | N | Y | Y | **Critically Low**  Y=3/15  PY=3/15  N=9/15 |
| Chou^82^ (2013) | Y | Y | N | Y | Y | N | Y | Y | PY | Y | N | Y | Y | N | Y | **Low**  Y=10/15  PY=1/15  N=4/15 |
| Garcia-Fernandez^84^ (2014) | N | N | Y | PY | N | N | N | N | N | N | Y | Y | Y | N | N | **Critically Low**  Y=4/15  PY=1/15  N=10/15 |
| He^86^  (2012) | N | N | N | N | Y | N | N | N | Y | N | N | N | N | Y | Y | **Critically Low**  Y=4/15  PY=0/15  N=11/15 |
| Huang^88^ (2021) | N | Y | N | PY | Y | Y | N | N | Y | N | Y | N | N | Y | Y | **Critically Low** Y=7/15  PY=1/15  N=7/15 |
| Mehicic^93^  (2024) | N | Y | N | N | Y | Y | N | N | N | N | NA | NA | N | Y | Y | **Critically Low**  Y=5/13  PY=0/13  N=8/13 |
| Pancorbo-Hidalgo^110^ (2006) | N | N | Y | PY | N | Y | N | PY | N | N | N | Y | Y | N | N | **Critically Low**  Y=4/15  PY=2/15  N=9/15 |
| Park^96^ (2016a) | N | N | N | PY | N | N | N | PY | N | N | N | N | N | Y | Y | **Critically Low**  Y=2/15  PY=2/15  N=11/15 |
| Park^97^ (2016b) | N | N | N | PY | N | Y | N | PY | N | N | N | N | N | Y | Y | **Critically Low**  Y=3/15  PY=2/15  N=10/15 |
| Park^98^  (2015) | N | N | N | PY | Y | Y | N | PY | N | N | N | Y | Y | Y | Y | **Critically Low**  Y=6/15  PY=2/15  N=7/15 |
| Pei^99^  (2023) | N | Y | N | Y | N | Y | N | N | Y | N | N | N | Y | N | Y | **Critically Low**  Y=6/15  PY=0/15  N=9/15 |
| Qu^100^  (2022) | N | Y | N | N | Y | Y | N | N | Y | N | N | N | Y | N | Y | **Critically Low**  Y=6/15  PY=0/15  N=9/15 |
| Tayyib^103^ (2013) | N | N | N | PY | N | N | N | N | N | N | NA | NA | N | N | N | **Critically Low**  Y=0/13  PY=1/13  N=12/13 |
| Wang^104^ (2022) | N | N | N | PY | Y | Y | N | N | N | N | Y | N | Y | N | Y | **Critically Low**  Y=5/15  PY=1/15  N=9/15 |
| Wei^105^  (2020) | N | N | N | PY | Y | Y | N | N | PY | N | N | N | Y | Y | N | **Critically Low**  Y=4/15  PY=2/15  N=9/15 |
| Wilchesky^106^ (2015) | N | N | N | PY | N | N | N | N | N | N | N | N | N | Y | Y | **Critically Low**  Y=2/15  PY=1/15  N=12/15 |
| Zhang^107^ (2021) | N | Y | N | PY | Y | Y | N | PY | Y | N | Y | N | Y | Y | Y | **Low**  Y=8/15  PY=2/15  N=5/15 |
| Zimmerman ^109^ (2018) | N | N | N | N | Y | N | N | N | N | N | NA | NA | N | N | N | **Critically Low** Y=1/13  PY=0/13  N=12/13 |
| **Summary** | **1/19 Yes** | **6/19 Yes** | **2/19 Yes** | **2/19 Yes**  **13/19 PY** | **12/19 Yes** | **11/19 Yes** | **1/19 Yes** | **1/19 Yes**  **7/19 PY** | **6/19 Yes**  **3/19 PY** | **1/19 Yes** | **4/16 Yes** | **4/16 Yes** | **9/19 Yes** | **10/19 Yes** | **14/19 Yes** |  |
|  |  |  |  |  |  |  |  |  |  |  |  |  |  |  |  |  |
| **Clinical effectiveness reviews** | | | | | | | | | | | | | | | | |
| Baris^79^  (2015) | N | N | N | PY | N | Y | N | N | N | N | NA | NA | N | N | N | **Critically Low** Y=1/13  PY=1/13  N=11/13 |
| Chou^82^ (2013) | Y | Y | N | Y | Y | N | Y | Y | Y | Y | NA | NA | Y | N | Y | **Moderate**  Y=10/13  PY=0/13  N=3/13 |
| Gaspar^85^ (2019) | Y | N | N | PY | Y | N | N | Y | N | N | NA | NA | N | Y | Y | **Critically Low**  Y=5/13  PY=1/13  N=7/13 |
| Health Quality Ontario^87^ (2009) | N | N | N | N | N | N | N | Y | N | N | NA | NA | Y | N | Y | **Critically Low**  Y=3/13  PY=0/13  N=10/13 |
| Kottner^90^ (2009) | N | N | N | PY | Y | Y | N | Y | PY | N | NA | NA | Y | Y | Y | **Critically Low**  Y=6/13  PY=2/13  N=5/13 |
| Lovegrove^91^ (2021) | Y | PY | N | PY | Y | Y | N | Y | PY | N | NA | NA | Y | Y | Y | **Low**  Y=7/13  PY=3/13  N=2/13 |
| Lovegrove^92^ (2018) | N | Y | N | PY | Y | Y | N | Y | PY | N | NA | NA | Y | Y | Y | **Low**  Y=7/13  PY=2/13  N=4/13 |
| Mehicic^93^  (2024) | Y | Y | N | N | Y | Y | N | N | PY | N | NA | NA | Y | N | Y | **Critically Low**  Y=6/13  PY=1/13  N=6/13 |
| Moore^94^ (2019) | Y | Y | N | Y | Y | N | Y | Y | Y | Y | NA | NA | Y | Y | Y | **High**  Y=11/13  PY=0/13  N=2/13 |
| Pancorbo-Hidalgo^110^ (2006) | N | N | Y | PY | N | Y | N | Y | PY | N | NA | NA | Y | N | N | **Critically Low** Y=4/13  PY=3/13  N=6/13 |
| Tayyib^103^ (2013) | N | N | N | PY | N | N | N | PY | N | N | NA | NA | N | N | N | **Critically Low** Y=0/13  PY=2/13  N=11/13 |
| **Summary** | **4/11 Yes** | **4/11 Yes**  **1/11 PY** | **1/11 Yes** | **2/11 Yes**  **7/11 PY** | **7/11 Yes** | **6/11 Yes** | **2/11 Yes** | **8/11 Yes**  **1/11 PY** | **2/11 Yes**  **5/11 PY** | **2/11 Yes** | **11/11 NA** | **11/11 NA** | **8/11 Yes** | **5/11 Yes** | **8/11 Yes** |  |
|  |  |  |  |  |  |  |  |  |  |  |  |  |  |  |  |  |
| Item 1 – Adequate research question/ inclusion criteria?; Item 2 – Protocol and justifications for deviations?; Item 3 – Reasons for study design inclusions?; Item 4 – Comprehensive search strategy?; Item 5 – Study selection in duplicate?; Item 6 – Data extraction in duplicate?; Item 7 – Excluded studies list (with justifications)?; Item 8 – Included studies description adequate?; Item 9 – Assessment of RoB/quality satisfactory?; Item 10 – Studies’ sources of funding reported?; Item 11 – Appropriate statistical synthesis method?; Item 12 – Assessment of impact of RoB on synthesised results?; Item 13 – Assessment of impact of RoB on review results?; Item 14 – Discussion/investigation of heterogeneity?; Item 15 – Conflicts of interest reported?  * Note that many items were not as applicable here, as the Dweekat 2023^83^ review is a methodological review; N – No; N/A – Not Applicable; PY – Partial Yes; RoB – Risk of Bias; Y – Yes. Further details on AMSTAR items are given in Appendix 4. | | | | | | | | | | | | | | | | |

### Table S4. Risk prediction tool characteristics, ascertained at review level

| **Name of tool (publication year)** | **Considered in included systematic reviews** | **Country; setting; patients; data source** | **Type of model; model development algorithm** | **Prediction horizon** | **Interval validation method** | **Predictors/domains in final model** | **N patients (n events)** | **Patient characteristics** | **Performance metrics** |
| --- | --- | --- | --- | --- | --- | --- | --- | --- | --- |
| Abruzzese (1985)^111^ | No | NS | Statistical; NS | NS | NS | General health; mental status; activity; mobility; continence; nutrition; oral nutrition intake; oral fluid intake; pre-disposing diseases (vascular disease, neuropathies, diabetes, anemias etc.) | NS | NS | NS |
| Admission PU-FIM model (2014)^112^ | Yes ^102^ | NS; rehabilitation units for SCI; NS; prospective | ML; LR, recursive partitioning analysis | Mean follow-up 36.5 (SD 31.4) days | NS | NS | 159 (21) | Mean (SD) age: 46.9 (19.1) Female: 22.0% | C-statistic: 0.77 (95% CI 0.65–0.86) |
| Andersen (1982)^113^ | Yes ^82 84 110^ | Denmark; acute hospital inpatient; adult; prospective | Statistical; NS | 10 days in-hospital observation; 3-months total observation | NS | NS | 3398 (40) | NS | NS |
| Arnell (1983)^114^ | Yes ^84^ | NS; NS; adult; NS | Statistical; NS | NS | NS | NS | NS | NS | NS |
| Berlowitz – 11-item  (1996)^115^ | Yes ^102^ | USA; long-term care; adult veterans; retrospective | Statistical; LR | 6 months | NS | NS | 31150 (1350) | Mean (SD) age: 70 (11.6) Female: 3% | O/E ratio: 1.0 (95% CI 0.95-1.05)  C-statistic: 0.75 (95% CI 0.74–0.76) |
| Berlowitz MDS risk-adjustment model (2001)^116^ | Yes ^102^ | USA; long-term care; adult nursing home residents; retrospective | Statistical; LR | 3 months | NS | NS | 14607 (905) | Mean (SD) age: 82.5 (10.9) Female: 75.4% | O/E ratio: 0.97 (95% CI 0.91-1.04)  C-statistic: 0.73 (95% CI 0.71–0.75) |
| Braden (1987)^117^ | Yes ^59 79 81 82 84 86 88 91-94 97 98 103-107 109 110^ | USA; hospital; elderly; NS | Statistical; NS | 12 weeks | NS | Sensory perception; moisture; activity; mobility; nutrition; friction and shear | 102 (28) | NS | NS |
| Braden – Baldwin 2-item (1998)^118^ | Yes ^102^ | NS; trauma and burn centres; NS; prospective | Statistical; LR | 26.5 days | NS | NS | 36 (11) | Mean (SD) age: 31.8 (10.9) Female: 27.8% | NS |
| Braden – Bergquist 2-item (2001)^119^ | Yes ^102^ | USA; long-term care; adult ($>$60y); retrospective | Statistical; Cox regression | Mean follow-up 60.6 (SD 94.3) days | NS | NS | 1684 (107) | Mean (SD) age: 76.4 (8.6) Female: 62.4% | NS |
| Braden – Bergquist 3-item (2001)^119^ | Yes ^102^ | USA; long-term care; adult ($>$60y); retrospective | Statistical; Cox regression | Mean 60.6 follow-up (SD 94.3) days | NS | NS | 1684 (107) | Mean (SD) age: 76.4 (8.6) Female: 62.4% | NS |
| Braden modified by Choi & Song (1991)^120 121^ | Yes ^82 84 96 103 107 109^ | Korea; ICU (neurological problems); NS; prospective | Statistical; NS | NS | NS | Body temperature; amount of medication (analgesics, sedation, anticoagulants); sensory perception; activity and mobility; moisture; nutrition; friction and shear | 146 (17) | Female: 39% | NS |
| Braden modified by Halfens/4-factor model (2000)^122^ | Yes ^84 103 107 109^ | Netherlands; hospital inpatients; NS; prospective | Statistical; stepwise LR | NS | NS | Sensory perception; moisture; friction and shear; age | 320 (47) | Mean age: 61 Female: 48%  Ethnicity: white 100% | NS |
| Braden modified by Kwong (2005)^123^ | Yes ^82 84^ | China; acute hospital inpatient; any; prospective | Statistical; NS | Mean of 11 days (range 5-21 days) | NS | NS | 429 (9) | Mean (SD) age: 54 (17) Female: 41% | NS |
| Braden modified by Pang & Wong (1998)^124^ | Yes ^96^ | NS; rehabilitation hospital (medical and orthopaedic); NS; NS | Statistical; NS | 2 weeks | NS | NS | 138 (NS, 20%) | NS | NS |
| Braden modified by Schue (1998)^125^ | Yes ^102^ | NS; rehabilitation units; adult males; retrospective | Statistical; LR | NS | NS | NS | 170 (9) | Mean (SD) age: 69.2 (10.9) All male | NS |
| COMHON (2011)^126^ | Yes ^107^ | Spain; ICU ($>$72h); NS; NS | Statistical; NS | NS | NS | NS | NS | NS | NS |
| Compton ICU model (2008)^127^ | Yes ^84 102^ | Germany; ICU; adult; retrospective | Statistical; LR | Median follow-up 6 (IQR 3-14) days | NS | NS | 698 (121) | Median (IQR) age: 66 (56-75.3) Female: 43.8% | C-statistic: 0.82 (95% CI 0.78–0.85) |
| Cubbin & Jackson (1991)^128^ | Yes ^80 84 96 107 109 110^ | UK; ICU; adult; NS | Statistical; NS | NS | NS | Age; weight; general skin condition; mental status; mobility; hemodynamics; nutrition; respiration; incontinence; hygiene | NS | NS | NS |
| Cubbin & Jackson revised (Jackson & Cubbin) (1999)^129^ | Yes ^82 84 103^ | UK; ICU; adult; NS | Statistical; NS | NS | NS | Age; weight; past medical history; general skin condition; mental condition; mobility; haemodynamics; respiration; oxygen requirement; nutrition; incontinence; hygiene | NS | NS | NS |
| Delmore scale (2015)^130^ | No | USA; hospital; any ($\geq$8y); NS | Statistical; NS | NS | NS | NS | NS | NS | NS |
| Douglas (1986)^131^ | Yes ^82 84 103 107 109 110^ | NS; ICU; NS; NS | Statistical; NS | NS | NS | Pain; activity; physical condition; incontinence; steroid therapy; diabetes; cytotoxic therapy; dyspnea | NS | NS | NS |
| DUPA (1995)^132^ | Yes ^84^ | USA; ICU; adult; NS | Statistical; NS | NS | NS | NS | 85 | NS | NS |
| Dutch CBO Score (1992)^133^ | Yes ^82^ | Netherlands; hospital; NS; retrospective | Statistical; NS | NS | NS | NS | NS | NS | NS |
| EMINA (2001)^134^ | Yes ^84 107 110^ | Spain; ICU (long-stay); adult; NS | Statistical; NS | 7 days | NS | NS | 673 (47) | NS | NS |
| EVARUCI scale (2001)^135^ | Yes ^84 107 109^ | Spain; ICU; NS; NS | Statistical; NS | NS | NS | NS | NS | NS | NS |
| Extended Braden (2000)^122^ | Yes ^82 103 107^ | Netherlands; hospital inpatient (medical and surgical); NS; prospective | Statistical; stepwise LR | NS | NS | NS | 320 (47) | Mean age years: 60.9 Female: 48% Ethnicity: white 100% | NS |
| Finnish risk assessment scale (2006)^14^ | No | Finland; long-term care; NS; prospective | Statistical; expert consensus and investigating agreement percentages | NS | NS | Urinary incontinence; activity; mental status; nutrition; mobility; sensory perception; skin condition; appetite; devices section; care methods section | Phase 1^a^: 43 raters, 6 patients Phase 2^b^: 64 (50 analysed) experts | NS | NS |
| Fragmment scale (2002)^136^ | Yes ^82 84 110^ ^102^ | Switzerland; hospital inpatient (acute, medical surgical); adult ($>$16y); prospective | Statistical; LR, Cox regression | Follow-up duration of 3 weeks, with mean follow-up 9 days | CV | NS | 1190 (182) | Mean (range) age: 61 (16-96) Female: 45.4% | C-statistic: 0.80 (95% CI 0.77–0.84) |
| Gosnell (1973)^137^ | Yes ^82 84 107^ | USA; ICU; NS; NS | Statistical; NS | NS | NS | Mental status; continence; movement control; ability to ambulate; process of food intake (evaluation includes recording of vital signs, skin condition and medications, but these are not scored) | NS | NS | NS |
| Hatanaka (2008)^138^ | Yes ^82 102^ | Japan; acute hospital inpatient; elderly; prospective | Statistical; LR, Cox regression | Mean follow-up 33 (range 5-79) days | NS | Haemoglobin; CRP; albumin; age; gender | 149 (38) | Mean (SD) age: 72 (11) Female: 30% | C-statistic: 0.79 (95% CI 0.66–0.88) |
| HPUR (2003)^139^ | Yes ^84^ | Sweden; palliative care; adult; NS | Statistical; NS | NS | NS | NS | 54 | NS | NS |
| Knoll Decubitus Ulcer Potential Scale (1988)^140^ | Yes ^82 84 110^ | USA; long-term care; adult ($>$65y); prospective | Statistical; NS | 28 days | NS | General health; mental health; activity; mobility; incontinence; oral nutrition intake; oral fluid intake; predisposing diseases | 60 (28) | Mean (range) age: 81 (65-97) Female: 80% Ethnicity: white 72%; black 15%; Asian 2%; unknown 11% | NS |
| Maelor score (2000)^141^ | Yes ^91 92^ | UK; NS; NS; NS | Statistical; NS | NS | NS | NS | NS | NS | NS |
| Mainland China | No | NS | NS; NS | NS | NS | NS | NS | NS | NS |
| Medley score (1987) | No | UK; acute medical ward, long-term care; NS; NS | Statistical; NS | NS | NS | NS | NS | NS | NS |
| ML Ahmad (2021)^142^ | Yes ^83^ | USA; inpatient, out-patient, nursing home; NS; prospective | ML; LR, other | NS | 10-fold CV | NS | 713 (NS, 52.3%) | NS | NS |
| ML Alderden [1] (2018)^143^ | Yes ^78 83 89 99 101 108^ | USA; surgical ICU, surgical cardiovascular ICU; adult; retrospective | ML; RF | NS | Split sample; 67% training 33% testing | Hypotension; Glasgow Coma Scale; oxygenation; BMI at admission; laboratory value (albumin, creatinine, glucose, haemoglobin, lactate, prealbumin); surgical time; age | 6376 (1. 516^c^; 2. 257^d^) | Mean (SD) age: 54 (19) Female: 37.7% Ethnicity: white 100% | Accuracy^e^: 0.790  AUC (SD):  1. 0.79  2. 0.79 |
| ML Alderden [2] (2021)^144^ | Yes ^83 108^ ^78^ | USA; surgical ICU; adult (≥18y); retrospective | ML; RF, MLP (ANN), other (AdaBoost, Gradient Boosting, LR) | NS | Split sample; 80% training 20% testing | Minimum albumin; minimum arterial PaO_2_; surgery duration; vasopressin infusion; length of ICU stay prior to HAPI; skin assessment; Braden scale scores | 5101 (NS, 6.5%) | NS | F1 score: 0.34  AUC (SD): 0.8 (0.02) |
| ML Anderson (2021)^145^ | Yes ^83^ ^78 99^ | USA; surgical ICU; adult (≥18y); retrospective | ML; LR, RF, NN, DL | NS | Split sample; 70% testing vs. 30% testing | NS | 23000 (738) | NS | Accuracy^e^: 0.86-0.99  Sensitivity: 0.67-1  Specificity: 0.91-0.99  PPV: 0.82-0.98  NPV: 0.88-1  AUC: 0.71-0.72 |
| ML Borlawsky (2007)^146^ | Yes ^83 102^ | USA; acute hospital inpatient; NS; retrospective | ML; DT | NS | 4-fold CV; tree pruning of the DT tool | NS | 3300 (206) | NS | NS |
| ML Cai (2021)^147^ | Yes ^83 100 101 108^ ^78^ | China; cardiovascular surgery; any; prospective | ML; DT, XGBoost | NS | NS | Age; gender; disease category; weight; duration of surgery; duration of cardiopulmonary bypass procedure | 149 (37) | NS | AUC: 0.81  Sensitivity: 0.08  Specificity: 1  PPV: 1  NPV: 0.77 |
| ML Charon (2022)^148^ | Yes ^83^ | France; nursing home; NS; NS | ML; RF, BN | NS | NS | NS | 3000 | NS | NS |
| ML Chen [1] (2018)^149^ | Yes ^83 89 100 101 108^ | China; cardiovascular surgery; NS; retrospective | ML; ANN | NS | Split sample; 70% training 30% testing | Length of surgery; disease category; age; perioperative corticosteroid administration | 149 (37) | Mean (SD) age: 49.8 (17.7)  Female 46.9% | F1 score: 0.19  Accuracy^e^: 0.815  Accuracy^e^: 0.28  Sensitivity: 0.11  Specificity: 0.79  PPV: 0.67  NPV: 0.21 |
| ML Chen [2] (2019)^150^ | Yes ^100^ | NS; CVD patients; adult; NS; NS | ML; LR | NS | NS | Preoperative haemoglobin value; blood sodium value; preoperative albumin; intraoperative mean body temperature; lowest mean arterial pressure; serum potassium value; smoking frequency; history of hypertension; age | 1163 (67) | NS | NS |
| ML Chen YC (2008)^151^ | Yes ^83^ | Taiwan; post-surgery; NS; retrospective | ML; LR, DT, SVM, other | NS | 3-fold CV | NS | 168 (NS, 4.8%) | NS | NS |
| ML Cheng [1] (2020)^152^ | Yes ^78^ | NS; hospital; NS; NS | ML; LR | NS | Split sample; 83% training 17% testing | Age; movement; sensory perception; response; moisture; perfusion; use of medical devices; compulsive position; hypoalbuminemia; HAPI; surgery | 2341 | NS | AUC: 0.87-0.94 |
| ML Cheng [2] (2021)^153^ | Yes ^83^ | China; hospital inpatient; elderly (>65y); retrospective | ML; RF, SVM, MLP, other | NS | 5-fold CV | NS | 245 (NS, 80%) | NS | NS |
| ML Cho (2011)^154^ | Yes ^83 100^ | Korea; ICU; adult (≥18y); NS | ML; LR, NN | NS | NS | Gender; BMI; transfer from ER; medical diagnosis; systolic blood pressure; diastolic blood pressure; number of urinations; number of defecations; any stompy; number of drain tubes; any catheterization; number of position changes; peripheral sensory; skin condition; albumin; number of sedatives administered; any strain; age; length of stay; any inotropic support; total parenteral nutrition; any sedation; number of analgesics administered | 21,114 for NN model; 21,069 for LR model (3348) | NS | NS |
| ML Choi (2020)^155^ | Yes ^108^ ^78^ | Korea; intubated ICU; adult (≥18y); prospective | ML; LR, BN (Gaussian NB) | NS | CV; split sample; 80% training 20% testing | Oral mucosal; bite-block or airway use; endotracheal tube; holder use; steroid use; vasopressor use; haematocrit; albumin | 27 (NS, 55.6%) | NS | AUC: 0.68-0.82  Sensitivity: 0.60-0.85  Specificity: 0.76-0.89  PPV: 0.23-0.37  NPV: 0.97-0.98 |
| ML Cichosz (2019)^156^ | Yes ^83 108^ | Denmark; any hospital (ICU, medical, surgical); adult (>20y); retrospective | ML; LR | NS | 10-fold CV; split sample; 65% training 35% testing | Gender; up and self-reliant; limitation in activity performance; mobility and willingness; consciousness | 383 (NS, 28.1% in training cohort, 18% in testing cohort) | NS | AUC: 0.82  Sensitivity: 0.43  Specificity: 0.94  PPV: 0.72  NPV: 0.92 |
| ML Cramer (2019)^157^ | Yes ^78 83 99 100 108^ | USA; ICU; adult (≥18y); retrospective | ML; LR, RF, SVM, MLP (ANN), other (GB, EN, NN) | NS | 5-fold CV; split sample 80% training 20% testing | Stage 1 PI within the first 24h; Glasgow Coma Scale; blood urea nitrogen; PaO_2;_ cardiac surgery recovery unit; albumin; medical ICU; pressure reduction device; mechanical ventilation; mean arterial pressure (top 10 predictors in two highest performing models) | 50851 (1690) | NS | Sensitivity: 0.49  PPV: 0.09  NPV: 0.71 |
| ML Delparte (2021)^158^ | Yes ^108^ | Canada; SCI rehabilitation centre; NS; retrospective | ML; LR, DT | NS | NS | PI history; ambulation; FIM toileting scores; FIM bed transfer scores | 807 (NS, 22%) | Mean age: 54 | AUC: 0.78  Sensitivity: 0.93  Specificity: 0.63  PPV: 0.4  NPV: 0.97 |
| ML Deng [1] (2016)^159^ | Yes ^89 99 100^ | China; ICU; adult; retrospective | ML; DT | NS | NS | Age; faecal incontinence; Braden total score; diastolic blood pressure | 468 (94) | Mean (SD) age: 57.81 (16.72) Female: 25.4% | AUC: 0.83 (95% 0.78-0.88)  Sensitivity: 0.81  Specificity: 0.70 |
| ML Deng [2] (2017)^160^ | Yes ^83 100 108^ | China; ICU, medical ICU; adult; retrospective | ML; LR, DT | NS | 10-fold CV | Age; ICU length of stay; diastolic blood pressure; albumin level; mechanical ventilation; total Braden score; faecal incontinence | 468 (94) | Mean (SD) age: 58 (17) Female: 25.4% | AUC: 0.93 (95% CI 0.88-0.97)  Sensitivity: 0.86  Specificity: 0.82  PPV: 0.76  NPV: 0.98 |
| ML Deschepper (2022) ^161^ | Yes ^78^ | NS; ICU; NS; prospective | ML; RF | NS | Split sample; 90% training 10% testing | Age; gender; diagnosis; Braden score; BMI; heart rate; mean arterial pressure; temperature; laboratory results; immunocompromised status | 13254 | NS | Accuracy^e^: 0.830  AUC: 0.785-0.792 |
| ML Do (2022)^162^ | Yes ^78 83 99^ | USA; hospital; adult; retrospective | ML; LR, RF, DT, KNN, NB | NS | 5-fold CV; split sample; 70% training 30% testing | Bed positions; laboratory tests (creatinine, lactate, pre-albumin, and albumin); clinical features; admission weight; BMI; activity; Braden assessment | 6742 (NS, 31.9%) | Average age: 61.5 Female: 47.3% | Accuracy^e^: 0.90-0.97  Sensitivity: 0.86-0.98  Specificity: 0.87-0.97  PPV: 0.81-0.96  NPV: 0.92-0.99  AUC: 0.90-0.97 |
| ML Eshetie (2023)^163^ | Yes ^78^ | NS; residential aged care; elderly; retrospective | ML; Fine-Gray Model | NS | Split sample; 80% training 20% testing | History of PIs; lower care needs mainly mobility; toileting; complex health care; medication assistance | 206540 | NS | AUC: 0.72-0.75 |
| ML Gao (2018)^164^ | Yes ^83 100^ | China; surgical; any; retrospective | ML; LR, DT | NS | NS | Application of external force during operation; lean body mass; time of operation $\geq$6h; prone position operation; cardiopulmonary bypass during operation; intraoperative blood loss | 1963 (48) | NS | NS |
| ML Goodwin (2020)^165^ | Yes ^108^ | USA; ICU; adult (≥16y); retrospective | ML; LR, SVM, CANTRIP, LSTM | NS | NS | NS | 35218 (NS, 39.8%) | NS | F1 score: 0.53  Accuracy^e^: 0.84  AUC: 0.87  Sensitivity: 0.72  Specificity: 0.85  PPV: 0.42 |
| ML Hou (2010)^166^ | Yes ^100^ | NS; hospital; NS; NS | ML; LR | NS | NS | Wet skin conditions; activity conditions; movement conditions; nutrition; mental awareness; bowel control; blood sugar; age; haemoglobin | 303 (46) | NS | NS |
| ML Hu (2020)^167^ | Yes ^78 83 99 100 108^ | China; hospital; adult; retrospective | ML; LR, DT, RF | NS | 10-fold CV; split sample; 50% training 50% testing | Skin integrity; systolic pressure; expression ability; capillary refill time; level of consciousness; eye-opening; level of mobility; emotional responses; diastolic pressure; skin properties; colour in the peripheral limbs; pulse rate | 11838 (161) | NS | F1 score: 0.08  AUC: 0.88-1  Sensitivity: 0.69-1  Specificity: 0.72-0.99  PPV: 0.79-0.99  NPV: 0.82-1 |
| ML Hyun (2019)^168^ | Yes ^83 100^ ^78^ | USA; ICU; adult; retrospective | ML; LR | NS | Split sample; 67% training 33% testing | Age; gender; weight; diabetes; vasopressor; isolation; endotracheal tube; ventilator episode; Braden score; ventilator days | 12654 (753) | NS | Accuracy^e^: 0.81  Sensitivity: 0.65  Specificity: 0.69  PPV: 0.211  NPV: 0.956  AUC: 0.737 |
| ML interRAI PURS (2010)^169^ | Yes ^102^ | Canada; long-term care; elderly; retrospective | ML; LR, DT | Follow-up 91 days | NS | NS | 14083 (503) | Mean (SD) age: 82.2 (10.2) Female: 69.2% | C-statistic: 0.71 (95% CI 0.68–0.73) |
| ML James (2021)^170^ | Yes ^78^ | USA; hospital; NS; retrospective | ML; EBM, DT, LR | NS | NS | Patient history (age and gender); vital signs (heart rate and blood pressure); lab tests (hemoglobin and creatinine levels); length of stay; procedures; medications | 100355 | NS | AUC: 0.60-0.79 |
| ML Jin (2017)^171^ | Yes ^83^ | Korea; hospital; adult; retrospective | ML; LR | NS | Split sample; 80% training 20% testing | NS | 11191 (NS, 20%) | NS | NS |
| ML Kaewprag [1] (2015)^172^ | Yes ^108^ | USA; ICU; adult (≥18y); retrospective | ML; LR, DT, SVM, NB, RF, KNN | NS | 10-fold CV | Braden subscale; medication (18 medication categories); diagnosis (61 comorbid conditions) | 7717 (NS, 7.6%) | NS | g-means: 0.618  AUC: 0.83  Sensitivity: 0.16  Specificity: 0.99  PPV: 0.56  NPV: 0.93 |
| ML Kaewprag [2] (2017)^173^ | Yes ^83 89 99 100 108^ | USA; ICU; adult; retrospective | ML; BN (chosen method), LR, DT, NN, RF, SVM | NS | Split sample; 67% training 33% testing | Braden subscale; medication (18 medication categories); diagnosis (61 comorbid conditions) | 7717 (590) | Mean (SD) age: 57.7 (15.9) Female: 42.6% | Accuracy^e^: 0.819  AUC (SD): 0.83 (0.01)  Sensitivity: 0.478  Specificity: 0.895  Sensitivity (SD): 0.46 (0.03)  Specificity: 0.91 (0.01)  PPV (SD): 0.29 (0.18)  NPV: 0.95 |
| ML Kim [1] (2006)^174^ | Yes ^100^ | USA; hospital (>4d stay); adult (≥18y); NS | ML; DT | NS | NS | Age; race; gender; visit type; diagnoses; procedures; tests/results; drugs | 826 (52) | NS | NS |
| ML Kim [2] (2006)^174^ | Yes ^100^ | USA; hospital (>4d stay); adult (≥18y); NS | ML; LOS | NS | NS | Presence of edema (cardiovascular system); presence of an indwelling foley catheter; presence of nutrition consult triggered for potential or actual nutritional imbalance; use of a wheelchair as an assistive device during the hospital stay; presence of a need for extra nursing care | 2347 (84) | NS | NS |
| ML Ladios-Martin (2020)^175^ | Yes ^78 83 99 100 108^ | Spain; ICU; adult (≥16y); retrospective | ML; LR, DT, RF, SVM, MLP (ANN), BN, other | NS | Split sample; 64% testing 36% testing | Medical service; days of oral antidiabetic agent or insulin therapy; ability to eat; number of red blood cell units transfused; haemoglobin range; PI present on admission; illness severity (total APACHE II score); gender; age; place of birth | 6694 (208) | Mostly aged 65-84 (61.0%)  Female: 34.2% | Accuracy^e^: 0.87  AUC: 0.88  Sensitivity: 0.75  Specificity: 0.88  PPV: 0.22  NPV: 0.99 |
| ML Lee (2021)^176^ | Yes ^83^ | Korea; nursing home; NS; NS | ML; RF, LR, SVM | NS | NS | NS | 60 | NS | NS |
| ML Levy (2022)^177^ | Yes ^83 99^ | USA; hospital inpatient (≥2d stay); adult (≥18y); retrospective | ML; LR, DT, RF, NB, XGBoost | NS | 5-fold CV; 80% training 20% testing | NS | 57227 (241) | Mean (SD) age: 65.5 (15.0) for PI present,  60.1 (18.4) for PI absent  Female: 48.2% | AUC: 0.91 |
| ML Li [1] (2019)^178^ | Yes ^83 89 99 100 108^ | China; hospice/end of life; adult; retrospective | ML; LR, DT, SVM, MLP, ANN | NS | K-fold CV (K not specified) | History of PIs, without cancer, excretion, activity/mobility, and skin condition/circulation | 2062 (1026) | Mean (SD) age: 75.5 (14.4) Female: 45.4% | g-means: 0.770 (DT); 0.779 (NN); 0.798 (SVM)  Accuracy^e^: 0.772 (DT); 0.781 (NN); 0.793 (SVM)  Sensitivity: 0.796 (DT); 0.814 (NN); 0.810 (SVM)  Specificity: 0.748 (DT); 0.749 (NN); 0.788 (SVM) |
| ML Li [2] (2020)^179^ | Yes ^100^ | NS; hospital; NS; NS | ML; SVM, NN | NS | NS | Department category; BMI; skin type; incontinence; poor eating/lack of appetite; feeling restricted; total assessment score; activity score | 554 (345) | NS | NS |
| ML Nakagami (2021)^180^ | Yes ^78 83 99 100 108^ | Japan; any inpatient; adult (≥20y); retrospective | ML; LR, RF, SVM, DT, XGBoost | NS | 5-fold CV; split sample; 70% training 30% testing | Paralysis (difficulty in moving around, difficulty in going up and down stairs, difficulty in transfer, difficulty in standing up, difficulty in keeping standing position); anorexia; age; gender; diagnoses/comorbidities; diet; pain; level of consciousness; skin condition; severity of illness; department type | 75353 (395) | Mean (SD) age: 66.5 (15.9) for PI present,  58.0 (18.5) for PI absent  Female: 47.2% | AUC (SD): 0.80 (0.02)  Sensitivity (SD): 0.78 (0.03)  Specificity (SD): 0.74 (0.04)  PPV: 0.01-0.02  NPV: 0.99 |
| ML Ossai (2021)^181^ | Yes ^83^ | Australia; acute care; any; retrospective | ML; DT, RF, MLP, KNN, LDA | NS | 10-fold CV | NS | 1014 | NS | NS |
| ML Park (2019)^182^ | Yes ^100^ | Korea; acute surgical care; NS; NS | ML; LR | NS | NS | Need for assistance with hygiene; decreased consciousness; Foley catheter; cardiac stimulant; oxygen therapy; low serum albumin; sensory perception impairment; impaired skin integrity; decreased mobility; surgery; nutrition consultation | 400 (80) | NS | NS |
| ML Setoguchi (2016)^183^ | Yes ^83 89 100 108^ | Japan; any hospital; any; retrospective | ML; DT | NS | 10-fold CV | Transfer activity; operation time; BMI | 8286 (NS, 0.62%) | Female: 44.4% | Accuracy^e^: 0.721  Sensitivity: 0.793  Specificity: 0.721 |
| ML Shui (2021)^184^ | Yes ^78^ | NS; hospital, ICU; adult; retrospective | ML; Fine-Gray Model | NS | Split sample; 70% training 30% testing | Age; BMI; lactate serum; Braden score; vasopressor use; antifungal medications | 18019 | NS | AUC: 0.56-0.92 |
| ML Šín (2022)^185^ | Yes ^78 83 99^ | USA; hospital, ICU; NS; retrospective | ML; LR, RF, SVM, MLP, KNN, BN, NB | NS | Split sample; 80% training 20% testing | Age; gender; ethnicity; total intake; total output; length of hospital stays; arterial oxygen saturation; systolic arterial blood pressure; height; daily weight; glucose (whole blood) | 9304 (4652) | NS | Accuracy^e^: 0.84-0.96  PPV: 0.75-0.94  NPV: 0.59-0.91  AUC: 0.77-0.94  F1 score: 0.93 |
| ML Song [1] (2021)^186^ | Yes ^83 99 100 108^ | China; hospital; adult (≥18y); retrospective | ML; DT, RF, SVM, MLP (ANN), KNN, LDA | NS | 10-fold CV; split sample; 50% training 50% testing | Gender; age; height; weight; total intake (mL); total output (mL); body temperature; systolic blood pressure (mmHg); blood glucose; length of stay (days); whether to stay in bed; whether to use restraint bands; surgery; diarrhoea; diabetes; fracture; Norton PI assessment; nutritional assessment; acceptance of passive turning over | 5814 (1673) | Mean (SD) age: 64.3 (18.3) for PI present,  51.9 (17.6) for PI absent  Female: 40.7% | F1 score: 0.83-1  Accuracy^e^: 0.79-1  AUC: 0.95-1  AUPRC: 0.96  Sensitivity: 0.99  Specificity: 0.99  PPV: 0.91-1  NPV: 0.87-1 |
| ML Song [2] (2021)^187^ | Yes ^78 83 99 100 108^ | USA; ICU or ≥24h in acute care; adult; retrospective | ML; LR, RF, SVM, MLP (ANN) | NS | 5-fold CV; split sample; 80% training 20% testing | PI; race; gender; age; Glasgow Coma Scale; level of consciousness; gait/ transferring; activity; pain score; diabetes; peripheral vascular disease; spinal cord injury; stroke; anaemia; albumin; blood urea nitrogen; chloride; potassium; sodium; creatinine; haemoglobin; white blood cell count; platelet blood count | 188512 (6165) | Mean (SD) age: 69.1 (15.5) for cases,  70.3 (7.3) for control  Female: 40.4% for cases, 49.0% for control | F1 score (SD): non-HAPI 0.81 (0.01); HAPI 0.86 (0.02)  Accuracy^e^ (SD): non-HAPI 0.85 (0.02); HAPI 0.88 (0.02)  AUC (SD): non-HAPI 0.92 (0.03); HAPI 0.94 (0.02)  Sensitivity (SD): non-HAPI 0.84 (0.02); HAPI 0.87 (0.03)  Specificity: non-HAPI 0.85 (0.02); HAPI 0.88 (0.02) |
| ML Sotoodeh (2020)^188^ | Yes ^108^ | USA; ICU; adult (≥16y); retrospective | ML; LR, RF, ANN | NS | NS | NS | 24457 | NS | F1 score (SD): 0.79 (0.02)  AUC (SD): 0.95 (0.01) |
| ML Sprigle (2020)^189^ | Yes ^89^ | USA; mobility related disabilities; NS; NS | ML; gradient boosting | NS | NS | Alzheimer’s disease; cerebral palsy; hemiplegia; multiple sclerosis; paraplegia/quadriplegia | 1252313 (NS, 6.9%) | Female: 54.1% | Sensitivity: 0.70  Specificity: 0.92 |
| ML SPURS (2019)^190^ | Yes ^78^ | NS; hospital, surgery; NS; retrospective | ML; LR | NS | Split sample; 70% training 30% testing | Age; female; ASA grade; BMI; Braden score; anemia; respiratory disease; hypertension | 269 | NS | Sensitivity: 0.40  Specificity: 0.70-0.95  PPV: 0.73  NPV: 0.08 |
| ML Su (2012)^191^ | Yes ^83 89 99 100 108^ | China; surgery; adult; prospective | ML; MTS (chosen method), LR, DT, SVM | NS | 4-fold CV; MTS model uses split sample 75% training 25% testing | Sex; age; weight; surgery type; body position during the operation; difference in temperature during surgery; surgical time (mins); air conditioning in operating room; number of electronic knives used in surgery | 168 (8) | Mean (SD) age: 65.4 (7.46) Female: 65.4% | F1 score: 0.38  F-score:  0.38 (MTS); 0.61 (SVM); 0.53 (DT); 0.67 (LR)  g-means: 0.82 (MTS); 0.81 (SVM); 0.7 (DT);  0.79 (LR)  Sensitivity: 0.76 (MTS); 0.67 (SVM); 0.50 (DT); 0.63 (LR)  Specificity: 0.89 (MTS); 0.95 (SVM); 0.98 (DT); 0.99 (LR) |
| ML Sun (2020)^192^ | Yes ^78^ | China; ICU; cancer patients; retrospective | ML; LR | NS | NS | Age; gender; diagnosis; cancer; anti-cancer therapy; Waterlow score; laboratory results; medications; length of stay; mechanical ventilation; APACHE score; blood purification | 486 | NS | Accuracy^e^: 0.83  Sensitivity: 0.66-0.81  Specificity: 0.78-0.96  AUC: 0.82-0.95 |
| ML Tang (2021)^193^ | Yes ^78^ | NS; surgery; NS; prospective | ML; LR | NS | NS | Braden score; preoperative fasting blood glucose level; emergency surgery; types of vasoactive drugs | 648 | NS | Sensitivity: 0.63  Specificity: 0.86  AUC: 0.74 |
| ML Vyas (2020)^194^ | Yes ^83 108^ | USA; ICU; adult (≥16y); retrospective | ML; XGBoost | NS | Split sample; 80% testing 20% testing | Mobility; activity; sensory perception; skin moisture; nutritional state; friction and shear | 13282 (NS, 16.8%) | NS | F1 score: 0.27  Accuracy^e^: 0.95  Sensitivity: 0.84  Specificity: 0.97  PPV: 0.87  NPV: 0.03 |
| ML Walther (2022)^195^ | Yes ^78 83 99^ | Germany; hospital; adult (≥19y); retrospective | ML; LR, BART, LASSO, RF | NS | 10-fold CV; split sample; 80% training 20% testing | Length of anesthesia; wards involved in care; admission reasons; ICU (with/without ventilation); age; sex; comorbidities | 149006 (4663) | Median age: 64  Female: 48.5% | Accuracy^e^: 0.52-0.55 Sensitivity: 0.04-0.10  Specificity: 1 PPV: 0.39-0.58  NPV: 0.98-0.99  AUC: 0.89-0.90 |
| ML Wang (2021)^196^ | Yes ^83^ | China; ICU; NS; retrospective | ML; RF, SVM, other | NS | Split sample; 67% testing 33% testing | NS | 246 (NS, 50%) | NS | NS |
| ML Waterlow 5-item (2000)^197^ | Yes ^102^ | UK; acute hospital; adult (>18y); retrospective | ML; LR, ANN | Follow-up 14 days | Split sample | NS | 422 (69) | Mean (SD) age: 64.8 (17.9) Female: 52.6% | NS |
| ML Xu (2022)^198^ | Yes ^78 83 99^ | China; ICU; adult; retrospective | ML; LR, DT, RF | NS | 5-fold CV; split sample; 70% training 30% testing | Reason for admission; clinical laboratory results; patients’ demographics; medical history; Braden scale | 618 (204) | NS | Accuracy^e^: 0.62-0.78 Sensitivity: 0.38-0.61 Specificity: 0.80-0.89  PPV: 0.54-0.65  NPV: 0.75-0.82  AUC: 0.72-0.88  F1 score: 0.49-0.62 |
| ML Yang (2019)^199^ | Yes ^89 99 100^ | China; tumour hospital; adult; prospective | ML; DT | NS | NS | Braden; inability to turn over; existing/potential damage to the skin; special circumstances | 611 (46) | Mean (SD) age: 61.8 (13.6) Female: 35.5% | Sensitivity: 0.848  Specificity: 0.774  AUC: 0.84 (95% CI 0.81- 0.87) |
| Norton (1962)^200^ | Yes ^79 82 84 87 91 96 97 103 107 109 110^ | UK; hospital; elderly; NS | Statistical; clinical expertise | NS | NS | Physical condition; mental status; activity; mobility; continence | 250 (60) | NS | NS |
| Norton modified by Bale (1995)^201^ | Yes ^82 84 87 110^ | UK; hospice/palliative care; adult; prospective | Statistical; NS | Group A mean (SD) days: 12 (6) Group B mean (SD) days: 13 (5) | NS | General physical condition; mobility; nutritional status; pain continence; special risk factors | 240 (38) | Mean age: 67 Women group A: 45% (group A), 59% (group B) | NS |
| Norton modified by Bienstein (1991)^202^ | Yes ^82 84 103^ | Germany; NS; adult; NS | Statistical; NS | NS | NS | Skin condition; cooperation/motivation; physical condition; additional diseases; mental state; incontinence; activity; mobility; age | NS | NS | NS |
| Norton modified by Ek 87 (1987)^203^ | Yes ^84^ | Sweden; long-term medical ward; adult; NS | Statistical; NS | NS | NS | NS | 367 (55) | Age range: 21-101 | NS |
| Norton modified by Ek 89 (1989)^204^ | No | Sweden; long-term medical ward; NS; NS | Statistical; NS | NS | NS | NS | NS | Mean age: 81 (women), 78 (men) | NS |
| Norton modified by Ek 91 (1991)^205^ | No | Sweden; long-term medical ward; NS; NS | Statistical; NS | NS | NS | NS | NS | Mean age: 80 | NS |
| Norton modified by Ek 97 (1997)^206^ | Yes ^87 110^ | Sweden; NS; NS; NS | NS; NS | NS | NS | NS | NS | NS | NS |
| Norton modified by Stotts (1988)^207^ | Yes ^82^ | USA; surgical (cardiovascular surgery and neurosurgery); adult (>18y); prospective | Statistical; NS | Follow-up to 3 weeks/to discharge | NS | Same items as the standard Norton scale with clarification regarding specific operational definitions | 387 (67) | Mean (range) age: 53 (17-86) Female: 47% | NS |
| Norton scale simplified (1998)^208^ | Yes ^102^ | Switzerland; acute hospital inpatient; adult (>16y); retrospective | Statistical; Cox regression | Follow-up duration/ length of stay: 9 days | NS | NS | 2373 (245) | Mean (SD) age: 63 (19) Female: 49% | NS |
| NOVA-4 | Yes ^84^ | NS; NS; adult; NS | Statistical; NS | NS | NS | NS | 187 | NS | NS |
| NPRU (1991)^209^ | No | NS | Statistical; NS | NS | NS | NS | NS | NS | NS |
| PARA (1994)^210^ | No | UK; ICU; NS; NS | Statistical; NS | NS | NS | NS | NS | NS | NS |
| prePURSE study tool (2006)^211^ | Yes ^102^ | Netherlands; acute hospital inpatient; adult (>18y); prospective | Statistical; LR | Follow-up 12 weeks | Resampling | NS | 1229 (121) | Mean (SD) age: 60.1 (16.7) Female: 54.8% | O/E ratio: 1.0 (95% CI 0.84–1.19)  C-statistic: 0.71 (95% CI 0.66–0.75) |
| Pressure Sore Predictor Scale (1987)^212^ | Yes ^84 110^ | UK; orthopaedic surgery and trauma; NS; NS | Statistical; NS | 3 weeks | NS | NS | 712 (in Lowthian87); 1244 (in '89) | NS | NS |
| Ramstadius (2000)^213^ | Yes ^82 85 91 92 94^ | Australia; hospital; NS; NS | Statistical; NS | NS | NS | Mobility; ability to independently reposition themselves; age; medication; skin integrity; temperature; decreased blood volume; dyspnoea and presence of an existing PI | NS | NS | NS |
| Risk Assessment Pressure Sore (RAPS) (2002)^214^ | Yes ^82 84 107 109 110^ | Sweden; hospital inpatient (medical, surgical, infection, orthopaedic, rehabilitation or geriatric ward); adult (>16y); prospective | Statistical; NS | Maximum follow-up 12 weeks; 50% of patients had ≤8 days follow-up | NS | NS | 488 (62) | Mean (SD) age: 70 (14) Female: 50% | NS |
| Rose/Cohen ICU model (2006)^215 216^ | Yes ^102^ | Canada; ICU; NS; prospective | Statistical; NS | Follow-up 8 days | NS | NS | 111 | NS | NS |
| S.S. (Suriada-Sanada) scale (2008)^217^ | Yes ^84 102 107 109^ | Indonesia; ICU; adult (≥18y); prospective | Statistical; LR, discriminant analysis | Mean follow-up 5.9 (SD 3.49) days | NS | Interface pressure; body temperature; cigarette smoking | 105 (35) | Mean (SD) age: 48.6 (17.5) Female: 31.4% | C-statistic: 0.89 (95% CI 0.83–0.93) |
| SCIPUS (1996)^218^ | No | USA; long-term care for SCI; adult; NS | Statistical; NS | NS | NS | Level of activity; levels of mobility; severity of SCI; complete SCI; autonomic dysreflexia or severe spasticity; urine incontinence or constantly moist; pre-existing conditions (age, tobacco use/smoking, pulmonary disease, cardiac disease or glucose >110mg dl^-1^, renal disease, impaired cognitive function); residence in a nursing home or hospital; nutrition (albumin <3.4 or total protein <6.4, anaemia haematocrit $\leq$36%) | 176 | NS | NS |
| SCIPUS-A (1999)^219^ | No | USA; SCI inpatient; adult; NS | Statistical; NS | NS | NS | Extent of paralysis; incontinence (moisture, continence); mobility; level of activity; nutrition (serum creatinine, albumin); pre-existing conditions (pulmonary disease) | 226 | NS | NS |
| Shannon (1982)^220^ | No | NS | Statistical; NS | NS | NS | NS | NS | NS | NS |
| Stratheden (1996)^221^ | No | UK; NS; elderly; NS | Statistical; NS | NS | NS | NS | NS | NS | NS |
| Sunderland scale (1995)^222^ | Yes ^84 107 109^ | UK; ICU; any; NS | Statistical; NS | NS | NS | NS | 15 | NS | NS |
| Surgical ICU risk assessment scale (2010)^223^ | Yes ^102^ | USA; surgical ICU; NS; prospective | Statistical; LR | NS | NS | NS | 369 (88) | Mean (SD) age years: 58.3 (19.3) Female: 43.6% | NS |
| The Northern Hospital Pressure Ulcer Prevention Plan (TNH-PUPP) model (2011)^224^ | Yes ^82 102^ | Australia; acute hospital inpatient; NS; retrospective | Statistical; LR | Mean follow-up 15.42 (SD 22.29) days | NS | NS | 342 (67) | Mean (SD) age years: 63 (19.8) Female: 54.3% | C-statistic: 0.86 (95% CI 0.81–0.90) |
| Vascular surgery PI risk score (2014)^225^ | Yes ^102^ | USA; cardiovascular surgery; adult; retrospective | Statistical; LR | Mean follow-up 7.08 (SD 7.44) days | Resampling | NS | 849 (101) | Mean (SD) age, years: 68.7 (13.0) | C-statistic: 0.85 (95% CI 0.81–0.89) |
| Waterlow (1985)^226^ | Yes ^82 84 85 90-92 94 96 97 103 104 107 109 110^ | UK; orthopaedic/generic; NS; NS | Statistical; NS | NS | NS | BMI; assessment of the skin; gender; age; malnutrition; incontinence; mobility; tissue malnutrition; neurological deficits; major surgery or trauma; medication | NS | NS | NS |
| Waterlow 12-item (1999)^227^ | Yes ^90^ | UK; hospital (acute and rehabilitation); elderly; NS | Statistical; NS | NS | NS | Skin type; mobility; poor nutrition; build; continence; appetite; medication; age; neurological deficit (including severe rheumatoid arthritis); major surgery; sex | NS | NS | NS |
| Waterlow scale simplified (2002)^228^ | Yes ^102^ | UK; acute hospital inpatient; adult (>65y); prospective | Statistical; LR | Follow-up 14 days | NS | NS | 213 (47) | Mean (SD) age years: 76.7 (8.1) | NS |
| Watkinson (1997)^229^ | Yes ^84^ | UK; NS; adult; NS | Statistical; NS | NS | NS | NS | 185 | NS | NS |

ANN – artificial neural network; ASA – American Society of Anaesthesiologists; AUC – area under curve; AUPRC – area under the precision-recall curve; BN – Bayesian network; BMI – body mass index; CANTRIP – reCurrent Additive Network for Temporal RIsk Prediction; CV – cross-validation; CVD – cardiovascular disease; DT – decision tree; EBM – explainable boosting machine; EN – elastic net; FIM – functional independence measure; GB – gradient boosting; ICU – intensive care unit; KNN – k-nearest neighbours; LDA – linear discriminant analysis; LOS – abbreviation not given by review authors; LR – logistic regression; LSTM – long short-term memory; ML – machine learning; MLP – multilayer perception; MTS – Mahalanobis-Taguchi system; NB – naïve Bayes; NN – neural network; NS – not stated; O/E – observed vs expected; OMPI – oral mucosal pressure injury; RF – random forest; RF – random forest; SCI – spinal cord injury; SD – standard deviation; SVM – support vector machine.

^a^Interrater agreement study.

^b^Expert opinion on clinical utility, reliability, usability, etc.

^c^HAPIs classified as stage 1 to 4, deep-tissue injury, or unstageable.

^d^HAPIs classified as stage 2 to 4, deep-tissue injury, or unstageable.

^e^Measure of accuracy not specified.

### Table S5. Prognostic model external validation study characteristics

| **Name of model/tool (publication year)** | **Model validation publication author** | **Model validation considered in reviews** | **Country; setting; patients; data source (validation population)** | **N patients (n events)** | **Recalibration of original model** | **Patient characteristics** | **Performance metrics** |
| --- | --- | --- | --- | --- | --- | --- | --- |
| Berlowitz 11-item (1996)^115^ | Berlowitz (1996)^a,115^ | ^102^ | NS; long-term care; NS; retrospective | 17946 (556) | NS | Mean (SD) age: 71.0 (11.4) Female: 3% | O/E ratio: 0.97 (95% CI 0.90–1.06) |
| Berlowitz MDS risk adjustment model (2001)^116^ | Berlowitz (2001) ^230^ | ^102^ | NS; long-term care; NS; retrospective | 13457 (608) | No | NS | C-statistic: 0.73 (95% CI 0.71–0.75)  O/E ratio: 0.91 (95% CI 0.84–0.98) |
| Compton ICU model (2008)^127^ | Compton (2008)^a,127^ | ^102^ | NS; specific acute care; NS; retrospective | 329 (56) | NS | Median age: 67  Female: 45% | C-statistic: 0.80 (95% CI 0.73–0.86) |
| ML interRAI PURS (2010)^169^ | Poss (2010)^a,169^ | ^102^ | NS; long-term care; NS; retrospective | 13062 (1267) | NS | NS | C-statistic: 0.61 (95% CI 0.59–0.62) |
| ML interRAI PURS (2010)^169^ | Poss (2010)^a,169^ | ^102^ | NS; long-term care; NS; retrospective | 73183 (1903) | NS | NS | C-statistic: 0.63 (95% CI 0.62–0.64) |
| ML Ladios-Martin (2020)^231^ | Ladios-Martin (2020)^a,231^ | ^99^ | NS; ICU; NS; NS | NS | NS | NS | NS |
| prePURSE study tool (2006)^211^ | Schoonhoven (2005)^b^ | ^102^ | NS; acute care hospital; NS; prospective | 1440 | NS | NS | NS |
| S.S. (Suriada-Sanada) scale (2008)^217^ | Suriadi 2008^a,217^ | ^102^ | NS; acute care hospital; NS; prospective | 253 (7) | NS | Mean (SD) age: 51.3 (19.4) Female: 37.5% | NS |
| The Northern Hospital Pressure Ulcer Prevention Plan (TNH-PUPP) model (2011)^224^ | Page 2011^a,224^ | ^102^ | NS; acute care hospital; NS; prospective | 165 (7) | NS | Mean (SD) age: 68 (18.4) Female: 47% | C-statistic: 0.90 (95% CI 0.66–0.98) |

ICU – intensive care unit; ML – machine learning; NS – not stated; O/E – observed versus expected; SD – standard deviation. ^a^ Model appears to be externally validated in the same publication as it was developed; ^b^ Model validation citation unclear; year for model development given as 2006 and year for model validation given as 2005.

### Table S6. Table of Predictors, by tool (predictors were reported for 66 tools), ascertained at review level except in the case of discrepancies between reviews

| **Tool name** | **General Health** | **Mental Status** | **Activity** | **Mobility** | **Ability to ambulate** | **Body position** | **Continence** | **Nutrition** | **Pre-disposing conditions** | **Age** | **Gender** | **Ethnicity or place of birth** | **Body** | **Skin** | **Isolation** | **Braden score** | **Receiving medical Tx/Rx** | **Laboratory values** | **Surgery duration** | **Length of stay** | **Pressure injury** | **Medical unit, ward, visit** | **Friction, shear, pressure** | **Pain** | **Hygiene** | **Smoking** | **Norton or Waterlow score** | **'Special'** (not explained) |
| --- | --- | --- | --- | --- | --- | --- | --- | --- | --- | --- | --- | --- | --- | --- | --- | --- | --- | --- | --- | --- | --- | --- | --- | --- | --- | --- | --- | --- |
| **No. of tools predictor appears in** | 18 | 21 | 21 | 28 | 6 | 3 | 23 | 22 | 32 | 33 | 21 | 5 | 22 | 21 | 2 | 14 | 30 | 27 | 7 | 8 | 8 | 6 | 5 | 3 | 3 | 2 | 2 | 2 |
| Abruzzese^111^ | Y | Y | Y | Y |  |  | Y | **Y x3** | Y |  |  |  |  |  |  |  |  |  |  |  |  |  |  |  |  |  |  |  |
| Braden^117^ |  | Y | Y | Y |  |  |  | Y |  |  |  |  |  | Y |  |  |  |  |  |  |  |  | Y |  |  |  |  |  |
| Braden - modified by Choi & Song^120 121^ | Y | Y | Y | Y |  |  |  | Y |  |  |  |  |  | Y |  |  | **Y x3** |  |  |  |  |  | Y |  |  |  |  |  |
| Braden - modified by Halfens / 4-factor model^122^ |  | Y |  |  |  |  |  |  |  | Y |  |  |  | Y |  |  |  |  |  |  |  |  | Y |  |  |  |  |  |
| Cubbin & Jackson^128^ | **Y x2** | Y |  | Y |  |  | Y | Y |  | Y |  |  | Y | Y |  |  |  |  |  |  |  |  |  |  | Y |  |  |  |
| Cubbin & Jackson (revised) "Jackson-Cubbin"^129^ | **Y x3** | Y |  | Y |  |  | Y | Y | Y | Y |  |  | Y | Y |  |  |  |  |  |  |  |  |  |  | Y |  |  |  |
| Douglas - based on Norton^131^ | Y |  | Y |  |  |  | Y |  | **Y x2** |  |  |  |  |  |  |  | **Y x2** |  |  |  |  |  |  | Y |  |  |  |  |
| Finnish risk assessment scale^14^ |  | **Y x2** | Y | Y |  |  | Y | **Y x2** |  |  |  |  |  | Y |  |  | **Y x2** |  |  |  |  |  |  |  |  |  |  |  |
| Gosnell^137^ |  | Y |  | Y | Y |  | Y | Y |  |  |  |  |  |  |  |  |  |  |  |  |  |  |  |  |  |  |  |  |
| Hatanaka^138^ |  |  |  |  |  |  |  |  |  | Y | Y |  |  |  |  |  |  | **Y x3** |  |  |  |  |  |  |  |  |  |  |
| Hyun^168^ |  |  |  |  |  |  |  |  | Y | Y | Y |  | Y |  | Y | Y | **Y x4** |  |  |  |  |  |  |  |  |  |  |  |
| Knoll Decubitus Ulcer Potential Scale^140^ | Y | Y | Y | Y |  |  | Y | **Y x2** | Y |  |  |  |  |  |  |  |  |  |  |  |  |  |  |  |  |  |  |  |
| ML Alderden [1]^143^ | Y |  |  |  |  |  |  |  | Y | Y |  |  | **Y x2** |  |  |  |  | **Y x6** | Y |  |  |  |  |  |  |  |  |  |
| ML Alderden [2]^144^ |  |  |  |  |  |  |  |  |  |  |  |  |  | Y |  | Y | Y | **Y x2** | Y | Y |  |  |  |  |  |  |  |  |
| ML Cai^147^ |  |  |  |  |  |  |  |  | Y | Y | Y |  | Y |  |  |  |  |  | **Y x2** |  |  |  |  |  |  |  |  |  |
| ML Chen [1]^149^ |  |  |  |  |  |  |  |  | Y | Y |  |  |  |  |  |  | Y |  | Y |  |  |  |  |  |  |  |  |  |
| ML Chen [2]^150^ |  |  |  |  |  |  |  |  | Y | Y |  |  | Y |  |  |  |  | **Y x5** |  |  |  |  |  |  |  | Y |  |  |
| ML Cheng [1]^152^ |  | **Y x2** | Y |  |  | Y |  |  |  | Y |  |  |  | Y |  |  | **Yx3** | Y |  |  | Y |  |  |  |  |  |  |  |
| ML Cho^154^ |  |  |  |  |  |  | **Y x3** | Y | **Y x5** | Y | Y |  | Y | Y |  |  | **Y x6** | **Y x3** |  | Y |  | Y |  |  |  |  |  |  |
| ML Choi^155^ |  |  |  |  |  |  |  |  |  |  |  |  | Y |  |  |  | **Y x4** | **Y x2** |  |  |  |  |  |  |  |  |  |  |
| ML Cichosz^156^ |  | Y | Y | Y | Y |  |  |  |  |  | Y |  |  |  |  |  |  |  |  |  |  |  |  |  |  |  |  |  |
| ML Cramer^157^ |  | Y |  |  |  |  |  |  |  |  |  |  |  |  |  |  | **Y x2** | **Y x3** |  |  | Y | **Y x3** |  |  |  |  |  |  |
| ML Delparte^158^ |  |  |  | Y | Y |  | Y |  |  |  |  |  |  |  |  |  |  |  |  |  | Y |  |  |  |  |  |  |  |
| ML Deng [1]^159^ |  |  |  |  |  |  | Y |  |  | Y |  |  |  |  |  | Y |  | Y |  |  |  |  |  |  |  |  |  |  |
| ML Deng [2]^160^ |  |  |  |  |  |  | Y |  |  | Y |  |  |  |  |  | Y | Y | **Y x2** |  | Y |  |  |  |  |  |  |  |  |
| ML Deschepper^161^ |  |  |  |  |  |  |  |  | **Y x2** | Y | Y |  | **Y x4** |  |  | Y |  | Y |  |  |  |  |  |  |  |  |  |  |
| ML Do^162^ | Y |  | Y |  |  | Y |  |  |  |  |  |  | **Y x2** |  |  | Y |  | **Y x4** |  |  |  |  |  |  |  |  |  |  |
| ML Eshetie^163^ |  |  |  | Y |  |  | Y |  |  |  |  |  |  |  |  |  | **Y x2** |  |  |  | Y |  |  |  |  |  |  |  |
| ML Gao^164^ |  |  |  |  |  |  |  |  |  |  |  |  | Y |  |  |  | **Y x4** |  | Y |  |  |  |  |  |  |  |  |  |
| ML Hou^166^ |  | Y | Y | Y |  |  | Y | Y |  | Y |  |  |  | Y |  |  |  | **Y x2** |  |  |  |  |  |  |  |  |  |  |
| ML Hu^167^ |  | **Y x4** |  | Y |  |  |  |  |  |  |  |  |  | **Y x3** |  |  |  | **Y x4** |  |  |  |  |  |  |  |  |  |  |
| ML Hyun^168^ |  |  |  |  |  |  |  |  | Y | Y | Y |  | Y |  | Y | Y | **Y x3** |  |  | Y |  |  |  |  |  |  |  |  |
| ML James^170^ |  |  |  |  |  |  |  |  |  | Y | Y |  | **Y x2** |  |  |  | **Y x2** | **Y x2** |  | Y |  |  |  |  |  |  |  |  |
| ML Kaewprag [1]^172^ |  |  |  |  |  |  |  |  | **Y x 61** |  |  |  |  |  |  | Y | **Y x 18** |  |  |  |  |  |  |  |  |  |  |  |
| ML Kaewprag [2]^173^ |  |  |  |  |  |  |  |  | **Y x 61** |  |  |  |  |  |  | Y | **Y x 18** |  |  |  |  |  |  |  |  |  |  |  |
| ML Kim [1]^174^ |  |  |  |  |  |  |  |  | Y | Y | Y | Y |  |  |  |  | **Y x2** | Y |  |  |  | Y |  |  |  |  |  |  |
| ML Kim [2]^232^ |  |  |  |  | Y |  |  | Y | Y |  |  |  |  |  |  |  | **Y x2** |  |  |  |  |  |  |  |  |  |  |  |
| ML Ladios-Martin^175^ | Y |  |  |  |  |  |  | Y | Y | Y | Y | Y |  |  |  |  | **Y x3** | Y |  |  | Y |  |  |  |  |  |  |  |
| ML Li [1]^178^ |  |  | Y | Y |  |  | Y |  | Y |  |  |  |  | Y |  |  |  |  |  |  | Y |  |  |  |  |  |  |  |
| ML Li [2]^179^ |  |  | Y | Y |  |  | Y | Y |  |  |  |  | Y | Y |  |  |  |  |  |  |  | Y |  |  |  |  |  |  |
| ML Nakagami^180^ |  | Y |  |  | **Y x5** |  |  | Y | **Y x3** | Y | Y |  |  | Y |  |  |  |  |  |  |  | Y |  | Y |  |  |  |  |
| ML Park^182^ |  | **Y x2** |  | Y |  |  |  | Y |  |  |  |  |  | Y |  |  | **Y x3** | Y |  |  |  |  |  |  | Y |  |  |  |
| ML Setoguchi^183^ |  |  | Y |  |  |  |  |  |  |  |  |  | Y |  |  |  |  |  | Y |  |  |  |  |  |  |  |  |  |
| ML Shui^184^ |  |  |  |  |  |  |  |  |  | Y |  |  | Y |  |  | Y | **Y x2** | Y |  |  |  |  |  |  |  |  |  |  |
| ML Šín^185^ |  |  |  |  |  |  |  | **Y x2** |  | Y | Y | Y | **Y x2** |  |  |  |  | **Y x3** |  | Y |  |  |  |  |  |  |  |  |
| ML Song [1]^186^ | Y |  | Y | **Y x2** |  |  | Y | **Y x3** | **Y x2** | Y | Y |  | **Y x2** |  |  |  | Y | **Y x2** |  | Y |  |  |  |  |  |  | Y |  |
| ML Song [2]^187^ |  | Y | Y |  | Y |  |  |  | **Y x5** | Y | Y | Y |  |  |  |  |  | **Y x9** |  |  | Y |  |  | Y |  |  |  |  |
| ML Sprigle^189^ |  |  |  |  |  |  |  |  | **Y x5** |  |  |  |  |  |  |  |  |  |  |  |  |  |  |  |  |  |  |  |
| ML SPURS^190^ | Y |  |  |  |  |  |  |  | **Y x3** | Y | Y |  | Y |  |  | Y |  |  |  |  |  |  |  |  |  |  |  |  |
| ML Su^191^ |  |  |  |  |  | Y |  |  |  | Y | Y |  | **Y x2** |  |  |  | **Y x3** |  | Y |  |  |  |  |  |  |  |  |  |
| ML Sun^192^ | Y |  |  |  |  |  |  |  | **Y x2** | Y | Y |  |  |  |  |  | **Y x4** | Y |  | Y |  |  |  |  |  |  | Y |  |
| ML Tang^193^ |  |  |  |  |  |  |  |  |  |  |  |  |  |  |  | Y | **Y x2** | Y |  |  |  |  |  |  |  |  |  |  |
| ML Vyas^194^ |  | Y | Y | Y |  |  |  | Y |  |  |  |  |  | Y |  |  |  |  |  |  |  |  | Y |  |  |  |  |  |
| ML Walther^195^ |  |  |  |  |  |  |  |  | Y | Y | Y |  |  |  |  |  | **Y x2** |  |  |  |  | Y |  |  |  |  |  |  |
| ML Xu^198^ |  |  |  |  |  |  |  |  | Y | Y | Y | Y |  |  |  | Y |  | Y |  |  |  |  |  |  |  |  |  |  |
| ML Yang^199^ |  |  |  | Y |  |  |  |  |  |  |  |  |  | Y |  | Y |  |  |  |  |  |  |  |  |  |  |  | Y |
| Norton^200^ | Y | Y | Y | Y |  |  | Y |  |  |  |  |  |  |  |  |  |  |  |  |  |  |  |  |  |  |  |  |  |
| Norton modified by Bale^201^ | Y |  |  | Y |  |  | Y | Y |  |  |  |  |  |  |  |  |  |  |  |  |  |  |  |  |  |  |  | Y |
| Norton modified by Bienstein^202^ | Y | **Y x2** | Y | Y |  |  | Y |  | Y | Y |  |  |  | Y |  |  |  |  |  |  |  |  |  |  |  |  |  |  |
| Norton modified by Stotts^207^ | Y | Y | Y | Y |  |  | Y |  |  |  |  |  |  |  |  |  |  |  |  |  |  |  |  |  |  |  |  |  |
| Ramstadius PI risk assessment^213^ | Y |  |  | **Y x2** |  |  |  |  | Y | Y |  |  |  | Y |  |  | Y | Y |  |  | Y |  |  |  |  |  |  |  |
| S.S. (Suriada-Sanada) scale^217^ | Y |  |  |  |  |  |  |  |  |  |  |  |  |  |  |  |  |  |  |  |  |  | Y |  |  | Y |  |  |
| SCIPUS^218^ |  |  | Y | Y |  |  |  |  | **Y x3** |  |  |  |  |  |  |  |  |  |  |  |  |  |  |  |  |  |  |  |
| SCIPUS-A^219^ |  |  | Y | Y |  |  | **Y x2** | Y | **Y x2** |  |  |  |  |  |  |  |  | **Y x2** |  |  |  |  |  |  |  |  |  |  |
| Waterlow^226^ |  |  |  | Y |  |  | Y | **Y x2** | Y | Y | Y |  | Y | Y |  |  | **Y x2** |  |  |  |  |  |  |  |  |  |  |  |
| Waterlow 12-item^227^ |  |  |  | Y |  |  | Y | **Y x2** | Y | Y | Y |  | Y | Y |  |  | **Y x2** |  |  |  |  |  |  |  |  |  |  |  |

**References**

1. Shea BJ, Reeves BC, Wells G, et al. AMSTAR 2: a critical appraisal tool for systematic reviews that include randomised or non-randomised studies of healthcare interventions, or both. *BMJ* 2017;358:j4008. doi: 10.1136/bmj.j4008

2. Alves AGP, Borges JWP, Brito MdA. Assessment of risk for pressure ulcers in intensive care units: an integrative review. *Revista de Pesquisa Cuidado é Fundamental Online* 2014;6(2):793-804. doi: 10.9789/2175-5361.2014.v6i2.793-804

3. Anthony D, Parboteeah S, Saleh M, et al. Norton, Waterlow and Braden scores: a review of the literature and a comparison between the scores and clinical judgement. *J Clin Nurs* 2008;17(5):646-53. doi: 10.1111/j.1365-2702.2007.02029.x

4. Cavalcante T, Queiroz A, Moura E, et al. UPDATING PF THE ASSISTANCE PROTOCOL FOR PRESSURE ULCER PREVENTION: EVIDENCE BASED PRACTICE. *Journal of Nursing UFPE online* 2016;10:1498. doi: 10.5205/reuol.7057-60979-3-SM-1.1003sup201618

5. Charalambous C, Koulori A, Vasilopoulos A, et al. Evaluation of the Validity and Reliability of the Waterlow Pressure Ulcer Risk Assessment Scale. *Med Arch* 2018;72(2):141-44. doi: 10.5455/medarh.2018.72.141-144

6. de Laat EH, Schoonhoven L, Pickkers P, et al. Epidemiology, risk and prevention of pressure ulcers in critically ill patients: a literature review. *Journal of wound care* 2006;15(6):269-75. doi: 10.12968/jowc.2006.15.6.26920

7. do Egito Cavalcanti de Farias A, Bezerra de Queiroz R. RISK FACTORS FOR THE DEVELOPMENT OF PRESSURE INJURY IN THE ELDERLY: INTEGRATIVE REVIEW. *Revista de Pesquisa: Cuidado e Fundamental* 2022;14(1) doi: 10.9789/2175-5361.rpcfo.v14.11423

8. Feuchtinger J, Halfens RJG, Dassen T. Pressure ulcer risk factors in cardiac surgery: A review of the research literature. *Heart and Lung: Journal of Acute and Critical Care* 2005;34:375-85. doi: 10.1016/j.hrtlng.2005.04.004

9. García-Fernández FP, Agreda JJ, Verdú J, et al. A new theoretical model for the development of pressure ulcers and other dependence-related lesions. *J Nurs Scholarsh* 2014;46(1):28-38. doi: 10.1111/jnu.12051 [published Online First: 2013/10/11]

10. Garrubba M, Melder A. Effectiveness of the Braden risk screening tool for pressure injuries: systematic review. *Centre for Clinical Effectiveness, Monash Innovation and Quality, Monash Health, Melbourne, Australia* 2017

11. Kelechi TJ, Arndt JV, Dove A. Review of pressure ulcer risk assessment scales. *J Wound Ostomy Continence Nurs* 2013;40(3):232-6. doi: 10.1097/WON.0b013e31828f2049

12. Keller BP, Wille J, van Ramshorst B, et al. Pressure ulcers in intensive care patients: a review of risks and prevention. *Intensive Care Med* 2002;28(10):1379-88. doi: 10.1007/s00134-002-1487-z [published Online First: 2002/09/07]

13. Ladd S, Ekanem U, Caffrey J. 540 A Systematic Review of Pressure Ulcers in Burn Patients: Risk Factors, Demographics, and Treatment Modalities. *Journal of Burn Care & Research* 2018;39:S233-S34. doi: 10.1093/jbcr/iry006.443

14. Lepisto M, Eriksson E, Hietanen H, et al. Developing a pressure ulcer risk assessment scale for patients in long-term care. *Ostomy/wound management* 2006;52(2):34-46.

15. Mendes Coqueiro J, Silva Brito R. MULTIPLE RISK FACTORS AND PREVENTIVE STRATEGIES OF PRESSURE ULCERS: SYSTEMATIC REVIEW. *Journal of Nursing UFPE / Revista de Enfermagem UFPE* 2013;7(10):6215-22. doi: 10.5205/reuol.4397-36888-6-ED.0710esp201321

16. Michel JM, Willebois S, Ribinik P, et al. As of 2012, what are the key predictive risk factors for pressure ulcers? Developing French guidelines for clinical practice. *Annals of Physical and Rehabilitation Medicine* 2012;55:454-65. doi: 10.1016/j.rehab.2012.08.003

17. Ming L, Qin G, Haobin Y, et al. Systematic review of pressure ulcer risk assessment scales for using in ICU patients. *Chinese Nursing Research* 2012;26(1):1-4.

18. Mordiffi SZ, Tho PC, Kent B, et al. Evaluating the effects of using the mobility assessment sub-scale within the Braden Scale on pressure ulcer incidence and preventive interventions in adult acute care settings: A systematic review. *JBI Libr Syst Rev* 2010;8(16 Suppl):1-13. doi: 10.11124/01938924-201008161-00006

19. Mortenson WB, Miller WC. A review of scales for assessing the risk of developing a pressure ulcer in individuals with SCI. *Spinal Cord* 2008;46:168-75. doi: 10.1038/sj.sc.3102129

20. Nadeem A, Healee D. UTILITY OF THE WATERLOW SCALE IN ACUTE CARE SETTINGS: A LITERATURE REVIEW. *Kai Tiaki Nursing Research* 2021;12(1):44-48.

21. O'Tuathail C, Taqi R. Evaluation of three commonly used pressure ulcer risk assessment scales. *Br J Nurs* 2011;20(6):S27-8, S30, S32 Passim. doi: 10.12968/bjon.2011.20.Sup2.S27

22. Rodriguez Torres MC, Soldevilla Agreda JJ. Clinical judgement or assessment scales to identify patients at risk of developing pressure ulcers? *Gerokomos* 2007;18:48-51.

23. Almeida ÍL, Garces T, Oliveira G, et al. Pressure injury prevention scales in intensive care units: an integrative review. 2020;21:e42053. doi: 10.15253/2175-6783.20202142053

24. Santos CT, Almeida Mde A, Oliveira MC, et al. [Development of the nursing diagnosis risk for pressure ulcer]. *Revista Gaucha de Enfermagem* 2015;36(2):113-21. doi: 10.1590/1983-1447.2015.02.49102

25. Šáteková L, Žiaková K. VALIDITY OF PRESSURE ULCER RISK ASSESMENT SCALES: REVIEW. *Central European Journal of Nursing & Midwifery* 2014;5(2):85-92.

26. Shahin E, Dassen T, Halfens R. Predictive validity of pressure ulcer risk assessment tools in intensive care patients. *Connect: The World of Critical Care Nursing* 2007;5:75-79. doi: 10.1891/1748-6254.5.3.75

27. Smet S, de Graaf A, Bernaerts K, et al. The Belgian pressure ulcer risk assessment project: Is assessing mobility and skin status a more accurate, reliable, and feasible approach to assess pressure ulcer risk in hospitalised patients? *Int Wound J* 2019;16(6):1577-78. doi: 10.1111/iwj.13240 [published Online First: 2019/10/13]

28. Solati S, Ahmadinezhad M, Alizadeh S. Predictive values of Braden and waterlow scales to assess the risk of pressure ulcer : Review article. *Int Electron J Med* 2016;5(2):12-17.

29. Taylor KJ, Bryant R, Boarini J. Assessment tools for the identification of patients at risk for the development of pressure sores: a review. *J Enterostomal Ther* 1988;15(5):201-5. doi: 10.1097/00152192-198809000-00030

30. Tran JP, McLaughlin JM, Li RT, et al. Prevention of Pressure Ulcers in the Acute Care Setting: New Innovations and Technologies. *Plast Reconstr Surg* 2016;138(3 Suppl):232s-40s. doi: 10.1097/prs.0000000000002644

31. Tschannen D, Anderson C. The pressure injury predictive model: A framework for hospital-acquired pressure injuries. *Journal of clinical nursing* 2020;29:1398-421. doi: 10.1111/jocn.15171

32. Walsh B, Dempsey L. Investigating the reliability and validity of the waterlow risk assessment scale: a literature review. *Clin Nurs Res* 2011;20(2):197-208. doi: 10.1177/1054773810389809 [published Online First: 2010/11/15]

33. Xu FR, Shi ZY, Yang FR. Risk assessment tools for pressure injury in intensive care patients: a review. *Connect: The World of Critical Care Nursing* 2018;12(1):16-19.

34. Alderden J, Rondinelli J, Pepper G, et al. Risk factors for pressure injuries among critical care patients: A systematic review. *International journal of nursing studies* 2017;71:97-114. doi: 10.1016/j.ijnurstu.2017.03.012

35. Barbosa da Silva J, Soares Rodrigues MC. Pressure ulcers in individuals with spinal cord injury: risk factors in neurological rehabilitation. *Rev Rene* 2020;21(1):1-9. doi: 10.15253/2175-6783.20202144155

36. Di Prinzio MF, Argento FJ, Barbalaco L, et al. [Risk factors for the development and recurrence of pressure ulcers in patients with spinal cord injury: A systematic review.]. *Revista de la Facultad de Ciencias Medicas de Cordoba* 2019;76(4):242-56. doi: 10.31053/1853.0605.v76.n4.24906

37. Haisley M, Sorensen JA, Sollie M. Postoperative pressure injuries in adults having surgery under general anaesthesia: systematic review of perioperative risk factors. *The British journal of surgery* 2020;21 doi: 10.1002/bjs.11448

38. Ham W, Schoonhoven L, Schuurmans MJ, et al. Pressure ulcers from spinal immobilization in trauma patients: A systematic review. *Journal of Trauma and Acute Care Surgery* 2014;76:1131-41. doi: 10.1097/TA.0000000000000153

39. Lima AR, Ribeiro Palmer C, Nogueira PC. RISK FACTORS AND PREVENTIVE INTERVENTIONS FOR PRESSURE INJURIES IN CANCER PATIENTS. *Revista Estima* 2021;19:1-13. doi: 10.30886/estima.v19.1005_IN

40. Lima Serrano M, González Méndez MI, Carrasco Cebollero FM, et al. Risk factors for pressure ulcer development in Intensive Care Units: Systematic review. *Medicina intensiva* 2017;41(6):339-46. doi: 10.1016/j.medin.2016.09.003

41. Marin J, Nixon J, Gorecki C. A systematic review of risk factors for the development and recurrence of pressure ulcers in people with spinal cord injuries. *Spinal Cord* 2013;51:522-27. doi: 10.1038/sc.2013.29

42. Rao AD, Preston AM, Strauss R, et al. Risk Factors Associated With Pressure Ulcer Formation in Critically Ill Cardiac Surgery Patients: A Systematic Review. *Journal of wound, ostomy, and continence nursing : official publication of The Wound, Ostomy and Continence Nurses Society / WOCN* 2016;43(3):242-7. doi: 10.1097/WON.0000000000000224

43. Reenalda J, Jannink M, Nederhand M, et al. Clinical use of interface pressure to predict pressure ulcer development: a systematic review. *Assistive technology : the official journal of RESNA* 2009;21:76-85. doi: 10.1080/10400430903050437

44. Shi C, Dumville JC, Cullum N. Skin status for predicting pressure ulcer development: A systematic review and meta-analyses. *International journal of nursing studies* 2018;87:14-25. doi: 10.1016/j.ijnurstu.2018.07.003

45. Siping S, Qixia J, Xiaoqing L. Risk factors of intraoperative acquired pressure injury: A systematic review and meta-analysis. *Clinical Focus* 2022;37(3):211.

46. Wynn M, Stephens M, Pradeep S, et al. Risk factors for the development and evolution of deep tissue injuries: A systematic review. *Journal of tissue viability* 2022 doi: 10.1016/j.jtv.2022.03.002

47. Zhang X, Haiju L, Yan Z, et al. Prevalence and Risk Factors of Postoperative Pressure Ulcers: A Systematic Review and Meta-analysis of Diagnostic Test. *Medicinal Plant* 2022;13(6)

48. Bulfone G, Bressan V, Morandini A, et al. Perioperative Pressure Injuries: A Systematic Literature Review. *Advances in skin & wound care* 2018;31:556-64. doi: 10.1097/01.ASW.0000544613.10878.ed

49. Chung ML, Widdel M, Kirchhoff J, et al. Risk Factors for Pressure Injuries in Adult Patients: A Narrative Synthesis. *International Journal of Environmental Research and Public Health* 2022;19 doi: 10.3390/ijerph19020761

50. Chung ML, Widdel M, Kirchhoff J, et al. Risk factors for pressure ulcers in adult patients: A meta-analysis on sociodemographic factors and the Braden scale. *Journal of clinical nursing* 2022;21 doi: 10.1111/jocn.16260

51. Coleman S, Gorecki C, Nelson EA, et al. Patient risk factors for pressure ulcer development: Systematic review. *International Journal of Nursing Studies* 2013;50:974-1003. doi: 10.1016/j.ijnurstu.2012.11.019

52. Dube A, Sidambe V, Verdon A, et al. Risk factors associated with heel pressure ulcer development in adult population: A systematic literature review. *Journal of Tissue Viability* 2022;31(1):84-103. doi: 10.1016/j.jtv.2021.10.007

53. Ferris A, Price A, Harding K. Pressure ulcers in patients receiving palliative care: A systematic review. *Palliative Medicine* 2019;33:770-82. doi: 10.1177/0269216319846023

54. Floyd NA. Effectiveness of Pressure Ulcer Protocols with the Braden Scale for Elderly Patients in the Intensive Care Unit: A Systematic Review. Walden University, 2018.

55. Gélis A, Dupeyron A, Legros P, et al. Pressure ulcer risk factors in persons with SCI: Part I: Acute and rehabilitation stages. *Spinal Cord* 2009;47(2):99-107. doi: 10.1038/sc.2008.107 [published Online First: 2008/09/02]

56. Gelis A, Dupeyron A, Legros P, et al. Pressure ulcer risk factors in persons with spinal cord injury part 2: the chronic stage. *Spinal Cord* 2009;47(9):651-61. doi: 10.1038/sc.2009.32

57. Liu ZF, Meng J, Jing N, et al. Effects of predictive nursing interventions on pressure ulcer in older bedridden patients: A meta‐analysis. *International Wound Journal* 2024;21(3):1-8. doi: 10.1111/iwj.14676

58. Moore Z, Avsar P, O'Connor T, et al. A systematic review of movement monitoring devices to aid the prediction of pressure ulcers in at-risk adults. *International Wound Journal* 2023;20(2):579-608. doi: 10.1111/iwj.13902

59. Mordiffi SZ, Kent B, Phillips N, et al. Use of mobility subscale for risk assessment of pressure ulcer incidence and preventive interventions: A systematic review. *JBI Library of Systematic Reviewis* 2011;9(56):2417-81.

60. Nixon J, Nelson EA, Rutherford C, et al. Pressure UlceR Programme Of reSEarch (PURPOSE): using mixed methods (systematic reviews, prospective cohort, case study, consensus and psychometrics) to identify patient and organisational risk, develop a risk assessment tool and patient-reported outcome Quality of Life and Health Utility measures. *Programme Grants for applied research* 2015;3(6)

61. Richardson A, Barrow I. Part 1: Pressure ulcer assessment - the development of Critical Care Pressure Ulcer Assessment Tool made Easy (CALCULATE). *Nursing in critical care* 2015;20:308-14. doi: 10.1111/nicc.12173

62. Teixeira RGF, Guedes lB, Lima NS, et al. Risk factors for pressure injury in critically ill polytraumatized patients: A systematic review. *Saude Coletiva* 2022;12(82):11774-81. doi: 10.36489/saudecoletiua.2022v12i82p11766-11781

63. Ting JJ, Garnett A. E-Health Decision Support Technologies in the Prevention and Management of Pressure Ulcers: A Systematic Review. *Computers, informatics, nursing: CIN* 2021;39:955-73. doi: 10.1097/CIN.0000000000000780

64. Toffaha KM, Simsekler MCE, Omar MA. Leveraging artificial intelligence and decision support systems in hospital-acquired pressure injuries prediction: A comprehensive review. *Artificial intelligence in medicine* 2023;141:102560. doi: 10.1016/j.artmed.2023.102560

65. Fuentelsaz Gallego C, Hernandez Faba E, Bermejo Caja C, et al. Review of literature on pressure ulcers in people aged 65 or over. [Spanish]. *Gerokomos* 2005;16:166-73.

66. Garcia-Fern, ez FP, Pancorbo-Hidalgo PL, et al. Risk assessment scales for pressure ulcer in intensive care units: A systematic review with metaanalysis. [Spanish]. *Gerokomos* 2013;24:82-89. doi: 10.4321/s1134-928x2013000200007

67. Kottner J, Tannen A, Dassen T. Interrater reliability of the Braden scale. *Pflege* 2008;21(2):85-94. doi: 10.1024/1012-5302.21.2.85

68. Nunes de Sousa JE, Alves Alencar Pereira LM, Silva Sousa BC, et al. ESCALAS UTILIZADAS PARA MENSURAR O RISCO DE LESÃO POR PRESSÃO EM PACIENTES HOSPITALIZADOS: UMA REVISÃO. *Enfermagem Atual in Derme* 2023;97(2):1-15. doi: 10.31011/reaid-2023-v.97-n.2-art.1573

69. Pancorbo-Hidalgo PL, García-Fernández FP, Soldevilla-Agreda JJ, et al. Pressure ulcers risk assessment: clinical practice in Spain and a meta-analysis of scales effectiveness. *Gerokomos* 2008;19(2):40-54.

70. Park SH, Park YS. Predictive validity of the Braden Scale for pressure ulcer risk: a meta-analysis. [Korean]. *Journal of Korean Academy of Nursing* 2014;44:595-607. doi: 10.4040/jkan.2014.44.6.595

71. 杨昭霞, 吴春梅, 戴靖华, et al. [Predictive validity of the Munro Scale for pressure injuries in surgical patients: A meta-analysis] Munro量表对手术患者压力性损伤预测效果的Meta分析. *Nursing of Integrated Traditional Chinese & Western Medicine* 2019;5(11):14-18. doi: 10.11997/nitcwm.201901104

72. Nayar SK, Li D, Ijaiya B, et al. Waterlow score for risk assessment in surgical patients: a systematic review. *Ann R Coll Surg Engl* 2021;103(5):312-17. doi: 10.1308/rcsann.2020.7136 [published Online First: 2021/04/14]

73. Zahia S, Garcia Zapirain MB, Sevillano X, et al. Pressure injury image analysis with machine learning techniques: A systematic review on previous and possible future methods. *Artificial Intelligence in Medicine* 2020;102:101742. doi: 10.1016/j.artmed.2019.101742

74. Moore ZE, Cowman S. Risk assessment tools for the prevention of pressure ulcers. *Cochrane Database Syst Rev* 2008(3):Cd006471. doi: 10.1002/14651858.CD006471.pub2 [published Online First: 2008/07/16]

75. Moore ZEH, Cowman S. Risk assessment tools for the prevention of pressure ulcers. *Cochrane Database of Systematic Reviews* 2014(2) doi: 10.1002/14651858.CD006471.pub3

76. Liao Y, Gao G, Mo L. Predictive accuracy of the Braden Q Scale in risk assessment for paediatric pressure ulcer: A meta-analysis. *Int J Nurs Sci* 2018;5(4):419-26. doi: 10.1016/j.ijnss.2018.08.003 [published Online First: 2018/10/10]

77. Ribeiro AP, Cruz I. How effective is the development of skin care in critically ill patients using the Braden Scale scores aiming to prevent the incidence of pressure ulcers? Sistematic Literature Review. *Journal of Specialized Nursing Care* 2013;6(1)

78. Barghouthi EaD, Owda AY, Asia M, et al. Systematic Review for Risks of Pressure Injury and Prediction Models Using Machine Learning Algorithms. *Diagnostics (Basel, Switzerland)* 2023;13(17) doi: 10.3390/diagnostics13172739

79. Baris N, Karabacak BG, Alpar SE. The Use of the Braden Scale in Assessing Pressure Ulcers in Turkey: A Systematic Review. *Advances in skin & wound care* 2015;28:349-57. doi: 10.1097/01.ASW.0000465299.99194.e6

80. Chen X, Diao D, Ye L. Predictive validity of the Jackson–Cubbin scale for pressure ulcers in intensive care unit patients: A meta‐analysis. *Nursing in Critical Care* 2023;28(3):370-78. doi: 10.1111/nicc.12818

81. Chen HL, Shen WQ, Liu P. A Meta-analysis to Evaluate the Predictive Validity of the Braden Scale for Pressure Ulcer Risk Assessment in Long-term Care. *Ostomy/wound management* 2016;62(9):20-8.

82. Chou R, Dana T, Bougatsos C, et al. Pressure ulcer risk assessment and prevention: a systematic comparative effectiveness review. *Annals of internal medicine* 2013;159(1):28-38.

83. Dweekat OY, Lam SS, McGrath L. Machine Learning Techniques, Applications, and Potential Future Opportunities in Pressure Injuries (Bedsores) Management: A Systematic Review. *International journal of environmental research and public health* 2023;20(1) doi: 10.3390/ijerph20010796

84. García-Fernández FP, Pancorbo-Hidalgo PL, Agreda JJS. Predictive Capacity of Risk Assessment Scales and Clinical Judgment for Pressure Ulcers: A Meta-analysis. *Journal of Wound Ostomy & Continence Nursing* 2014;41(1):24-34. doi: 10.1097/01.WON.0000438014.90734.a2

85. Gaspar S, Peralta M, Marques A, et al. Effectiveness on hospital-acquired pressure ulcers prevention: a systematic review. *International Wound Journal* 2019;16(5):1087-102. doi: 10.1111/iwj.13147

86. He W, Liu P, Chen HL. The Braden Scale cannot be used alone for assessing pressure ulcer risk in surgical patients: a meta-analysis. *Ostomy/wound management* 2012;58:34-40.

87. Ontario HQ. Pressure ulcer prevention: an evidence-based analysis. *Ontario health technology assessment series* 2009;9(2):1-104.

88. Huang C, Ma Y, Wang C, et al. Predictive validity of the braden scale for pressure injury risk assessment in adults: A systematic review and meta-analysis. *Nursing open* 2021;8:2194-207. doi: 10.1002/nop2.792

89. Jiang M, Ma Y, Guo S, et al. Using Machine Learning Technologies in Pressure Injury Management: Systematic Review. *JMIR Medical Informatics* 2021;9(3):e25704. doi: 10.2196/25704

90. Kottner J, Dassen T, Tannen A. Inter- and intrarater reliability of the Waterlow pressure sore risk scale: A systematic review. *International Journal of Nursing Studies* 2009;46:369-79. doi: 10.1016/j.ijnurstu.2008.09.010

91. Lovegrove J, Ven S, Miles SJ, et al. Comparison of pressure injury risk assessment outcomes using a structured assessment tool versus clinical judgement: A systematic review. *Journal of Clinical Nursing* 2021 doi: 10.1111/jocn.16154 [published Online First: 2021/12/01]

92. Lovegrove J, Miles S, Fulbrook P. The relationship between pressure ulcer risk assessment and preventative interventions: a systematic review. *Journal of wound care* 2018;27(12):862-75.

93. Mehicic A, Burston A, Fulbrook P. Psychometric properties of the Braden scale to assess pressure injury risk in intensive care: A systematic review. *Intensive & critical care nursing* 2024;83:103686. doi: 10.1016/j.iccn.2024.103686

94. Moore ZEH, Patton D. Risk assessment tools for the prevention of pressure ulcers. *Cochrane Database of Systematic Reviews* 2019 doi: 10.1002/14651858.CD006471.pub4

95. Pancorbo-Hidalgo PL, Garcia-Fernandez FP, Lopez-Medina IM, et al. Risk assessment scales for pressure ulcer prevention: a systematic review. *J Adv Nurs* 2006;54(1):94-110. doi: 10.1111/j.1365-2648.2006.03794.x

96. Park SH, Lee HS. Assessing Predictive Validity of Pressure Ulcer Risk Scales- A Systematic Review and Meta-Analysis. *Iranian journal of public health* 2016;45(2):122-33.

97. Park SH, Lee YS, Kwon YM. Predictive Validity of Pressure Ulcer Risk Assessment Tools for Elderly: A Meta-Analysis. *Western journal of nursing research* 2016;38:459-83. doi: 10.1177/0193945915602259

98. Park SH, Choi YK, Kang CB. Predictive validity of the Braden Scale for pressure ulcer risk in hospitalized patients. *Journal of Tissue Viability* 2015;24:102-13. doi: 10.1016/j.jtv.2015.05.001

99. Pei J, Guo X, Tao H, et al. Machine learning-based prediction models for pressure injury: A systematic review and meta-analysis. *Int Wound J* 2023 doi: 10.1111/iwj.14280 [published Online First: 2023/06/20]

100. Qu C, Luo W, Zeng Z, et al. The predictive effect of different machine learning algorithms for pressure injuries in hospitalized patients: A network meta-analyses. *Heliyon* 2022;8(11):e11361. doi: 10.1016/j.heliyon.2022.e11361

101. Ribeiro F, Fidalgo F, Silva A, et al. Literature review of machine-learning algorithms for pressure ulcer prevention: Challenges and opportunities: MDPI 2021.

102. Shi C, Dumville JC, Cullum N. Evaluating the development and validation of empirically-derived prognostic models for pressure ulcer risk assessment: A systematic review. *International journal of nursing studies* 2019;89:88-103. doi: 10.1016/j.ijnurstu.2018.08.005

103. Tayyib NAH, Coyer F, Lewis P. Pressure ulcers in the adult intensive care unit: a literature review of patient risk factors and risk assessment scales. *Journal of Nursing Education and Practice* 2013;3(11):28-42.

104. Wang N, Lv L, Yan F, et al. Biomarkers for the early detection of pressure injury: A systematic review and meta-analysis. *Journal of Tissue Viability* 2022;31:259-67. doi: 10.1016/j.jtv.2022.02.005

105. Wei M, Wu L, Chen Y, et al. Predictive Validity of the Braden Scale for Pressure Ulcer Risk in Critical Care: A Meta-Analysis. *Nursing in critical care* 2020;25:165-70. doi: 10.1111/nicc.12500

106. Wilchesky M, Lungu O. Predictive and concurrent validity of the Braden scale in long-term care: A meta-analysis. *Wound Repair and Regeneration* 2015;23:44-56. doi: 10.1111/wrr.12261

107. Zhang Y, Zhuang Y, Shen J, et al. Value of pressure injury assessment scales for patients in the intensive care unit: Systematic review and diagnostic test accuracy meta-analysis. *Intensive & critical care nursing* 2021;64:103009. doi: 10.1016/j.iccn.2020.103009

108. Zhou Y, Yang X, Ma S, et al. A systematic review of predictive models for hospital-acquired pressure injury using machine learning. *Nursing open* 2022;30 doi: 10.1002/nop2.1429

109. Zimmermann GS, Cremasco MF, Zanei SSV, et al. Pressure injury risk prediction in critical care patients: an integrative review. *Texto & Contexto-Enfermagem* 2018;27(3)

110. Pancorbo-Hidalgo PL, Garcia-Fern, ez FP, et al. Risk assessment scales for pressure ulcer prevention: a systematic review. *Journal of advanced nursing* 2006;54(1):94-110. doi: 10.1111/j.1365-2648.2006.03794.x

111. Abruzzese RS. Early assessment and prevention of pressure sores. *Chronic ulcers of the skin New york: McGraw-Hill* 1985:1-19.

112. DeJong G, Hsieh CHJ, Brown P, et al. Factors Associated with Pressure Ulcer Risk in Spinal Cord Injury Rehabilitation. *American Journal of Physical Medicine & Rehabilitation* 2014;93(11):971-86. doi: 10.1097/phm.0000000000000117

113. Andersen KE, Jensen O, Kvorning SA, et al. Prevention of pressure sores by identifying patients at risk. *Br Med J (Clin Res Ed)* 1982;284(6326):1370-1. doi: 10.1136/bmj.284.6326.1370

114. Arnell I. Treating decubitus ulcers: two methods that work. *Nursing* 1983;13(6):50-5. doi: 10.1097/00152193-198306000-00022

115. Berlowitz DR, Ash AS, Brandeis GH, et al. Rating long-term care facilities on pressure ulcer development: Importance of case-mix adjustment. *Annals of Internal Medicine* 1996;124(6):557-63. doi: 10.7326/0003-4819-124-6-199603150-00003

116. Berlowitz DR, Brandeis GH, Morris JN, et al. Deriving a risk-adjustment model for pressure ulcer development using the Minimum Data Set. *Journal of the American Geriatrics Society* 2001;49(7):866-71. doi: 10.1046/j.1532-5415.2001.49175.x

117. Braden B, Bergstrom N. A Conceptual Schema for the Study of the Etiology of Pressure Sores. *Rehabilitation Nursing* 1987;12(1):8-16. doi: 10.1002/j.2048-7940.1987.tb00541.x

118. Baldwin KM, Ziegler SM. Pressure ulcer risk following critical traumatic injury. *Advances in wound care : the journal for prevention and healing* 1998;11(4):168-73.

119. Bergquist S. Subscales, subscores, or summative score: evaluating the contribution of Braden Scale items for predicting pressure ulcer risk in older adults receiving home health care. *J Wound Ostomy Continence Nurs* 2001;28(6):279-89. doi: 10.1067/mjw.2001.119012

120. Choi KS, Song MS. Test of predictive validity for the new pressure risk assessment scale. *Journal of Korean Academy of Adult Nursing* 1991;3(1):19-28.

121. Song M, Choi KS. Factors predicting development of decubitus ulcers among patients admitted for neurological problems. *The Journal of Nurses Academic Society* 1991;21(1):16-26.

122. Halfens R, Van Achterberg T, Bal R. Validity and reliability of the Braden scale and the influence of other risk factors: a multi-centre prospective study. *International Journal of Nursing Studies* 2000;37(4):313-19.

123. Kwong E, Pang S, Wong T, et al. Predicting pressure ulcer risk with the modified Braden, Braden, and Norton scales in acute care hospitals in Mainland China. *Appl Nurs Res* 2005;18(2):122-8. doi: 10.1016/j.apnr.2005.01.001

124. Pang SM, Wong TK. Predicting pressure sore risk with the Norton, Braden, and Waterlow scales in a Hong Kong rehabilitation hospital. *Nursing Research* 1998;47(3):147-53.

125. Schue RM, Langemo DK. Pressure ulcer prevalence and incidence and a modification of the Braden Scale for a rehabilitation unit. *Journal of wound, ostomy, and continence nursing : official publication of The Wound, Ostomy and Continence Nurses Society* 1998;25(1):36-43. doi: 10.1016/s1071-5754(98)90011-0

126. Cobos Vargas A, Garofano Jerez J, Guardia Mesa M. Design and validation of a new rating scale to estimate the risk of pressure ulcer in patients attended in critical care units. *Connect: The World of Critical Care Nursing* 2011;8(2):41.

127. Compton F, Hoffmann F, Hortig T, et al. Pressure ulcer predictors in ICU patients: nursing skin assessment versus objective parameters. *J Wound Care* 2008;17(10):417-20, 22-4. doi: 10.12968/jowc.2008.17.10.31304

128. Cubbin B, Jackson C. Trial of a pressure area risk calculator for intensive therapy patients. *Intensive Care Nursing* 1991;7(1):40-44.

129. Jackson C. The revised Jackson/Cubbin Pressure Area Risk Calculator. *Intensive Crit Care Nurs* 1999;15(3):169-75. doi: 10.1016/s0964-3397(99)80048-2

130. Delmore B, Lebovits S, Suggs B, et al. Risk Factors Associated With Heel Pressure Ulcers in Hospitalized Patients. *Journal of Wound, Ostomy and Continence Nursing* 2015;42(3):242-48. doi: 10.1097/WON.0000000000000134

131. Prichard V. Calculating the risk. *Nursing times* 1986;2:59-61.

132. Jiricka MK, Ryan P, Carvalho MA, et al. Pressure ulcer risk factors in an ICU population. *Am J Crit Care* 1995;4(5):361-7.

133. Centraal Begeleidingsorgaan voor de Intercollegiale Toetsing (CBO). Development and validation of a pressure ulcer risk assessment tool for acute hospital patients. Utrecht: CBO, 1992.

134. Fuentelsaz C. Validation of the EMINA scale: tool for the evaluation of risk of developing pressure ulcers in hospitalized patients. *Enferm Clin [Internet]* 2001;11(3):97-103.

135. González-Ruiz J, Carrero AG, Blázquez MH, et al. Factores de riesgo de las úlceras por presión en pacientes críticos. *Enfermería Clinica* 2001;11(5):184-90.

136. Perneger TV, Raë AC, Gaspoz JM, et al. Screening for pressure ulcer risk in an acute care hospital: development of a brief bedside scale. *J Clin Epidemiol* 2002;55(5):498-504. doi: 10.1016/s0895-4356(01)00514-5

137. Gosnell DJ. An assessment tool to identify pressure sores. *Nursing research* 1973;22(1):55-58.

138. Hatanaka N, Yamamoto Y, Ichihara K, et al. A new predictive indicator for development of pressure ulcers in bedridden patients based on common laboratory tests results. *Journal of Clinical Pathology* 2008;61(4):514-18. doi: 10.1136/jcp.2007.050195

139. Henoch I, Gustafsson M. Pressure ulcers in palliative care: development of a hospice pressure ulcer risk assessment scale. *Int J Palliat Nurs* 2003;9(11):474-84. doi: 10.12968/ijpn.2003.9.11.11872

140. Towey AP, Erland SM. Validity and reliability of an assessment tool for pressure ulcer risk. *Decubitus* 1988;1(2):40-8.

141. Moore Z, Pitman S. Towards establishing a pressure sore prevention and management policy in an acute hospital setting. *The All Ireland Journal of Nursing and Midwifery* 2000;1(1):7-11.

142. Machine learning approaches for pressure injury prediction. 2021 IEEE 9th International Conference on Healthcare Informatics (ICHI); 2021. IEEE.

143. Alderden J, Pepper GA, Wilson A, et al. Predicting Pressure Injury in Critical Care Patients: A Machine-Learning Model. *Am J Crit Care* 2018;27(6):461-68. doi: 10.4037/ajcc2018525

144. Alderden J, Drake KP, Wilson A, et al. Hospital acquired pressure injury prediction in surgical critical care patients. *BMC Med Inform Decis Mak* 2021;21(1):12. doi: 10.1186/s12911-020-01371-z [published Online First: 20210106]

145. Anderson C, Bekele Z, Qiu Y, et al. Modeling and prediction of pressure injury in hospitalized patients using artificial intelligence. *BMC Med Inform Decis Mak* 2021;21(1):253. doi: 10.1186/s12911-021-01608-5 [published Online First: 20210830]

146. Borlawsky T, Hripcsak G. Evaluation of an automated pressure ulcer risk assessment model. *Home Health Care Management & Practice* 2007;19(4):272-84.

147. Cai JY, Zha ML, Song YP, et al. Predicting the Development of Surgery-Related Pressure Injury Using a Machine Learning Algorithm Model. *Journal of Nursing Research* 2021;29(1) doi: 10.1097/jnr.0000000000000411

148. Charon C, Wuillemin PH, Belmin J. Learning Bayesian Networks for the Prediction of Unfavorable Health Events in Nursing Homes. *Stud Health Technol Inform* 2022;294:147-48. doi: 10.3233/SHTI220423

149. Chen HL, Yu SJ, Xu Y, et al. Artificial Neural Network: A Method for Prediction of Surgery-Related Pressure Injury in Cardiovascular Surgical Patients. *J Wound Ostomy Continence Nurs* 2018;45(1):26-30. doi: 10.1097/won.0000000000000388

150. Chen Y, Wu B, Qian Q, et al. Development and validation of an intraoperatively acquired pressure ulcer risk prediction model for adults receiving cardiovascular surgery. *J Nurs Sci* 2019;34

151. Pressure Ulcers Prediction Using Support Vector Machines. 2008 4th International Conference on Wireless Communications, Networking and Mobile Computing; 2008. IEEE.

152. Cheng H, Sun X, Ji X, et al. Risk factors and the potential of nomogram for predicting hospital-acquired pressure injuries. *International Wound Journal* 2020;17(4):974-86. doi: 10.1111/iwj.13362

153. Cheng F-M, Jin Y-J, Chien C-W, et al. The application of braden scale and rough set theory for pressure injury risk in elderly male population. *Journal of Men's Health* 2021;17(4):156-65.

154. Cho IS, Chung E. Predictive Bayesian Network Model Using Electronic Patient Records for Prevention of Hospital-Acquired Pressure Ulcers. *Journal of Korean Academy of Nursing* 2011;41(3):423-31. doi: 10.4040/jkan.2011.41.3.423

155. Choi BK, Kim MS, Kim SH. Risk prediction models for the development of oral-mucosal pressure injuries in intubated patients in intensive care units: A prospective observational study. *Journal of Tissue Viability* 2020;29(4):252-57. doi: <https://doi.org/10.1016/j.jtv.2020.06.002>

156. Cichosz SL, Voelsang AB, Tarnow L, et al. Prediction of In-Hospital Pressure Ulcer Development. *Adv Wound Care (New Rochelle)* 2019;8(1):1-6. doi: 10.1089/wound.2018.0803 [published Online First: 20190105]

157. Cramer EM, Seneviratne MG, Sharifi H, et al. Predicting the Incidence of Pressure Ulcers in the Intensive Care Unit Using Machine Learning. *EGEMS (Wash DC)* 2019;7(1):49. doi: 10.5334/egems.307 [published Online First: 20190905]

158. Delparte JJ, Flett HM, Scovil CY, et al. Development of the spinal cord injury pressure sore onset risk screening (SCI-PreSORS) instrument: a pressure injury risk decision tree for spinal cord injury rehabilitation. *Spinal Cord* 2021;59(2):123-31. doi: 10.1038/s41393-020-0510-y

159. Deng X, Wang Q, Li M, et al. Predicting the risk of hospital-acquired pressure ulcers in intensive care unit patients based on decision tree. *Chin J Prac Nurs* 2016;32:485-89.

160. Deng XH, Yu T, Hu AL. Predicting the Risk for Hospital-Acquired Pressure Ulcers in Critical Care Patients. *Critical Care Nurse* 2017;37(4):E1-E11. doi: 10.4037/ccn2017548

161. Deschepper M, Labeau SO, Waegeman W, et al. Heterogeneity hampers the identification of general pressure injury risk factors in intensive care populations: A predictive modelling analysis. *Intensive and Critical Care Nursing* 2022;68:103117. doi: 10.1016/j.iccn.2021.103117

162. Do Q, Lipatov K, Ramar K, et al. Pressure Injury Prediction Model Using Advanced Analytics for At-Risk Hospitalized Patients. *Journal of patient safety* 2022;18(7):e1083-e89.

163. Eshetie TC, Moldovan M, Caughey GE, et al. Development of a Multivariable Prediction Model for Risk of Hospitalization With Pressure Injury After Entering Residential Aged Care. *Journal of the American Medical Directors Association* 2023;24(3):299-306.e9. doi: 10.1016/j.jamda.2022.12.009

164. Gao L, Yang LN, Li XQ, et al. The use of a logistic regression model to develop a risk assessment of intraoperatively acquired pressure ulcer. *Journal of Clinical Nursing* 2018;27(15-16):2984-92. doi: 10.1111/jocn.14491

165. Goodwin TR, Demner-Fushman D. A customizable deep learning model for nosocomial risk prediction from critical care notes with indirect supervision. *Journal of the American Medical Informatics Association* 2020;27(4):567-76. doi: 10.1093/jamia/ocaa004

166. Hou L, Yao, L., 2010. Logistic regression analysis of risk factors of pressure sore and building of discrimnant analysis model. *Shanghai Nurs* 2010;1

167. Hu Y-H, Lee Y-L, Kang M-F, et al. Constructing Inpatient Pressure Injury Prediction Models Using Machine Learning Techniques. *CIN: Computers, Informatics, Nursing* 2020;38(8):415-23. doi: 10.1097/cin.0000000000000604

168. Hyun S, Moffatt-Bruce S, Cooper C, et al. Prediction Model for Hospital-Acquired Pressure Ulcer Development: Retrospective Cohort Study. *Jmir Medical Informatics* 2019;7(3) doi: 10.2196/13785

169. Poss J, Murphy KM, Woodbury MG, et al. Development of the interRAI Pressure Ulcer Risk Scale (PURS) for use in long-term care and home care settings. *BMC geriatrics* 2010;10:67. doi: 10.1186/1471-2318-10-67

170. James A. Machine Learning Risk Assessment Model for Hospital Acquired Pressure Injuries. 2021

171. Jin Y, Jin T, Lee SM. Automated Pressure Injury Risk Assessment System Incorporated Into an Electronic Health Record System. *Nurs Res* 2017;66(6):462-72. doi: 10.1097/nnr.0000000000000245

172. Kaewprag P, Newton C, Vermillion B, et al. Predictive Modeling for Pressure Ulcers from Intensive Care Unit Electronic Health Records. *AMIA Jt Summits Transl Sci Proc* 2015;2015:82-6. [published Online First: 2015/03/25]

173. Kaewprag P, Newton C, Vermillion B, et al. Predictive models for pressure ulcers from intensive care unit electronic health records using Bayesian networks. *Bmc Medical Informatics and Decision Making* 2017;17 doi: 10.1186/s12911-017-0471-z

174. Predictive modeling for the prevention of hospital-acquired pressure ulcers. AMIA Annual Symposium Proceedings; 2006. AMIA Symposium.

175. Ladios-Martin M, Fernández-de-Maya J, Ballesta-López F-J, et al. Predictive modeling of pressure injury risk in patients admitted to an intensive care unit. *American Journal of Critical Care* 2020;29(4):e70-e80.

176. Lee SK, Shin JH, Ahn J, et al. Identifying the Risk Factors Associated with Nursing Home Residents' Pressure Ulcers Using Machine Learning Methods. *Int J Environ Res Public Health* 2021;18(6) doi: 10.3390/ijerph18062954 [published Online First: 2021/03/13]

177. Levy JJ, Lima JF, Miller MW, et al. Machine Learning Approaches for Hospital Acquired Pressure Injuries: A Retrospective Study of Electronic Medical Records. *Front Med Technol* 2022;4:926667. doi: 10.3389/fmedt.2022.926667 [published Online First: 2022/06/16]

178. Li HL, Lin SW, Hwang YT. Using Nursing Information and Data Mining to Explore the Factors That Predict Pressure Injuries for Patients at the End of Life. *Cin-Computers Informatics Nursing* 2019;37(3):133-41. doi: 10.1097/cin.0000000000000489

179. Li Q, 李清, 苏强, et al. Pressure Injury Analysis and Prediction Based on Machine Learning Methods. *同济大学学报 (自然科学版)(英文版)* 2020;48(10):1530-36.

180. Nakagami G, Yokota S, Kitamura A, et al. Supervised machine learning-based prediction for in-hospital pressure injury development using electronic health records: A retrospective observational cohort study in a university hospital in Japan. *International Journal of Nursing Studies* 2021;119 doi: 10.1016/j.ijnurstu.2021.103932

181. Ossai CI, O’Connor L, Wickramasighe N. Real-Time Inpatients Risk Profiling in Acute Care: A Comparative Study of Falls and Pressure Injuries Vulnerabilities. *University of Maribor: Maribor, Slovenia* 2021: 35–50.

182. Park SK, Park HA, Hwang H. Development and Comparison of Predictive Models for Pressure Injuries in Surgical Patients: A Retrospective Case-Control Study. *J Wound Ostomy Continence Nurs* 2019;46(4):291-97. doi: 10.1097/won.0000000000000544

183. Setoguchi Y, Ghaibeh AA, Mitani K, et al. Predictability of Pressure Ulcers Based on Operation Duration, Transfer Activity, and Body Mass Index Through the Use of an Alternating Decision Tree. *Journal of Medical Investigation* 2016;63(3-4):248-55. doi: 10.2152/jmi.63.248

184. Shui AM, Kim P, Aribindi V, et al. Dynamic Risk Prediction for Hospital-Acquired Pressure Injury in Adult Critical Care Patients. *Critical Care Explorations* 2021;3(11):e0580. doi: 10.1097/cce.0000000000000580

185. Šín P, Hokynková A, Marie N, et al. Machine learning-based pressure ulcer prediction in modular critical care data. *Diagnostics* 2022;12(4):850.

186. Song J, Gao Y, Yin PB, et al. The Random Forest Model Has the Best Accuracy Among the Four Pressure Ulcer Prediction Models Using Machine Learning Algorithms. *Risk Management and Healthcare Policy* 2021;14:1175-87. doi: 10.2147/rmhp.S297838

187. Song W, Kang M-J, Zhang L, et al. Predicting pressure injury using nursing assessment phenotypes and machine learning methods. *Journal of the American Medical Informatics Association* 2021;28(4):759-65. doi: 10.1093/jamia/ocaa336

188. Sotoodeh M, Gero ZH, Zhang W, et al. Pressure Ulcer Injury in Unstructured Clinical Notes: Detection and Interpretation. *AMIA Annu Symp Proc* 2020;2020:1160-69. [published Online First: 20210125]

189. Sprigle S, McNair D, Sonenblum S. Pressure Ulcer Risk Factors in Persons with Mobility-Related Disabilities. *Adv Skin Wound Care* 2020;33(3):146-54. doi: 10.1097/01.ASW.0000653152.36482.7d

190. Aloweni F, Ang SY, Fook-Chong S, et al. A prediction tool for hospital-acquired pressure ulcers among surgical patients: Surgical pressure ulcer risk score. *Int Wound J* 2019;16(1):164-75. doi: 10.1111/iwj.13007 [published Online First: 2018/10/05]

191. Su CT, Wang PC, Chen YC, et al. Data Mining Techniques for Assisting the Diagnosis of Pressure Ulcer Development in Surgical Patients. *Journal of Medical Systems* 2012;36(4):2387-99. doi: 10.1007/s10916-011-9706-1

192. Sun Z-W, Guo M-R, Yang L-Z, et al. Risk factor analysis and risk prediction model construction of pressure injury in critically ill patients with cancer: a retrospective cohort study in China. *Medical Science Monitor: International Medical Journal of Experimental and Clinical Research* 2020;26:e926669-1.

193. Tang Z, Li N, Xu J. Construction of a Risk Prediction Model for Intraoperative Pressure Injuries: A Prospective, Observational Study. *Journal of PeriAnesthesia Nursing* 2021;36(5):473-79. doi: 10.1016/j.jopan.2020.11.006

194. Vyas K, Samadani A, Milosevic M, et al. Additional value of augmenting current subscales in braden scale with advanced machine learning technique for pressure injury risk assessment. 2020 IEEE International Conference on Bioinformatics and Biomedicine (BIBM): IEEE, 2020:2993-95.

195. Walther F, Heinrich L, Schmitt J, et al. Prediction of inpatient pressure ulcers based on routine healthcare data using machine learning methodology. *Scientific Reports* 2022;12(1):5044.

196. Wang Y, Jiang X, Yu K, et al. Infrared thermal images classification for pressure injury prevention incorporating the convolutional neural networks. *IEEE Access* 2021;9:15181-90.

197. Anthony D, Clark M, Dallender J. An optimization of the Waterlow score using regression and artificial neural networks. *Clinical Rehabilitation* 2000;14(1):102-09. doi: 10.1191/026921500670250429

198. Xu J, Chen D, Deng X, et al. Development and validation of a machine learning algorithm–based risk prediction model of pressure injury in the intensive care unit. *International Wound Journal* 2022;19(7):1637-49.

199. Yang Q, Wang G, Jiang B, et al. Study on risk prediction model of unavoidable pressure ulcers in cancer patients based on decision tree. *Journal of Nursing Science* 2019;34(13):4-7.

200. Norton D. Geriatric nursing problems. *Int Nurs Rev* 1962;9:39-41.

201. Bale S, Finlay I, Harding KG. Pressure sore prevention in a hospice. *J Wound Care* 1995;4(10):465-8. doi: 10.12968/jowc.1995.4.10.465

202. Bienstein C. Risikopatienten erkennen mit der erweiterten Nortonskala [Risk patients detected with the extended Norton scale]. Dekubitus - Prophylaxe undTherapie. Frankfurt/Main: Verlag Krankenpflege 1991.

203. Ek AC. Prediction of pressure sore development. *Scand J Caring Sci* 1987;1(2):77-84. doi: 10.1111/j.1471-6712.1987.tb00603.x

204. Ek AC, Unosson M, Bjurulf P. The modified Norton scale and the nutritional state. *Scandinavian journal of caring sciences* 1989;3(4):183-87.

205. Ek A-C, Unosson M, Larsson J, et al. The development and healing of pressure sores related to the nutritional state. *Clinical Nutrition* 1991;10(5):245-50.

206. Ek AC, Nordstrom M, Berglund B. Quality indicators for patients with or with risk of developing pressure ulcers. In: Idwall E, ed. Quality Indicators in Nursing Omvardnad 1. Stockhold: Spri publications 1997.

207. Stotts NA, Paul SM. Pressure ulcer development in surgical patients. *Advances in Skin & Wound Care* 1988;1(3):24.

208. Perneger TV, Gaspoz JM, Rae AC, et al. Contribution of individual items to the performance of the norton pressure ulcer prediction scale. *Journal of the American Geriatrics Society* 1998;46(10):1282-86. doi: 10.1111/j.1532-5415.1998.tb04547.x

209. Comparison of pressure sore risk calculators. Proceedings of the first European conference on advances in wound management; 1991.

210. Birtwistle J. Pressure sore formation and risk assessment in intensive care. *Care of the Critically Ill* 1994;10:154-54.

211. Schoonhoven L, Grobbee DE, Donders ART, et al. Prediction of pressure ulcer development in hospitalized patients: a tool for risk assessment. *Quality & Safety in Health Care* 2006;15(1):65-70. doi: 10.1136/qshc.2005.015362

212. Lowthian P. The practical assessment of pressure sore risk. *Care–Science and Practice* 1987;5(4):3-7.

213. Ramstadius B. Preventing institution-acquired pressure ulcers. *Australian Nursing and Midwifery Journal* 2000;7(10):34.

214. Lindgren M, Unosson M, Krantz AM, et al. A risk assessment scale for the prediction of pressure sore development: reliability and validity. *Journal of advanced nursing* 2002;38(2):190-99.

215. Rose P, Cohen R, Amsel R. Development of a scale to measure the risk of skin breakdown in critically ill patients. *Am J Crit Care* 2006;15:337.

216. Cohen SR, Rose P. Development of a scale to measure the risk of skin breakdown in the critically ill. *The Canadian Nurse* 2005;101(1):11.

217. Suriadi Sanada H, Sugama J, Thigpen B, et al. Development of a new risk assessment scale for predicting pressure ulcers in an intensive care unit. *Nursing in critical care* 2008;13(1):34-43.

218. Salzberg CA, Byrne DW, Cayten CG, et al. A NEW PRESSURE ULCER RISK ASSESSMENT SCALE FOR INDIVIDUALS WITH SPINAL CORD INJURY1. *American Journal of Physical Medicine & Rehabilitation* 1996;75(2):96-104.

219. Salzberg C, Byrne D, Kabir R, et al. Predicting pressure ulcers during initial hospitalization for acute spinal cord injury. *Wounds-A Compendium of Clinical Research and Practice* 1999;11(2):45-57.

220. M. S. Pressure sores. In: C. N, ed. Concept Clarification in Nursing Rockville, Md: Aspen 1982:357-82.

221. McCormack HC. A pressure sore risk scale for use with older people. *Professional nurse (London, England)* 1996;11(10):673-76.

222. Lowery MT. A pressure sore risk calculator for intensive care patients: 'the Sunderland experience'. *Intensive Crit Care Nurs* 1995;11(6):344-53. doi: 10.1016/s0964-3397(95)80452-8

223. Slowikowski GC, Funk M. Factors Associated With Pressure Ulcers in Patients in a Surgical Intensive Care Unit. *Journal of Wound Ostomy and Continence Nursing* 2010;37(6):619-26. doi: 10.1097/WON.0b013e3181f90a34

224. Page KN, Barker AL, Kamar J. Development and validation of a pressure ulcer risk assessment tool for acute hospital patients. *Wound Repair and Regeneration* 2011;19(1):31-37. doi: 10.1111/j.1524-475X.2010.00647.x

225. Corniello AL, Moyse T, Bates J, et al. Predictors of pressure ulcer development in patients with vascular disease. *Journal of vascular nursing : official publication of the Society for Peripheral Vascular Nursing* 2014;32(2):55-62. doi: 10.1016/j.jvn.2013.07.002

226. Waterlow J. Pressure sores: a risk assessment card. *Nursing Times* 1985;81:49-55.

227. Cook M, Hale C, Watson B. Interrater reliability and the assessment of pressure-sore risk using an adapted Waterlow Scale. *Clinical Effectiveness in Nursing* 1999;3(2):66-74.

228. Papanikolaou P, Clark M, Lyne PA. Improving the accuracy of pressure ulcer risk calculators: some preliminary evidence. *International Journal of Nursing Studies* 2002;39(2):187-94. doi: 10.1016/s0020-7489(01)00011-6

229. Watkinson C. Developing a pressure sore risk assessment scale. *Prof Nurse* 1997;12(5):341-6, 48.

230. Berlowitz DR, Brandeis GH, Anderson JJ, et al. Evaluation of a risk-adjustment model for pressure ulcer development using the Minimum Data Set. *J Am Geriatr Soc* 2001;49(7):872-6. doi: 10.1046/j.1532-5415.2001.49176.x

231. Ladios-Martin M, Fernández-de-Maya J, Ballesta-López FJ, et al. Predictive Modeling of Pressure Injury Risk in Patients Admitted to an Intensive Care Unit. *Am J Crit Care* 2020;29(4):e70-e80. doi: 10.4037/ajcc2020237

232. Cho I, Park I, Kim E, et al. Using EHR data to predict hospital-acquired pressure ulcers: a prospective study of a Bayesian Network model. *Int J Med Inform* 2013;82(11):1059-67. doi: 10.1016/j.ijmedinf.2013.06.012 [published Online First: 20130724]

1. Search strategies for EMBASE in Ovid Syntax, In Health Information Research Unit Hedges project.Ontario:HIRU;2022: [Health Information Research Unit - HIRU ~ Search Strategies for EMBASE in Ovid Syntax (mcmaster.ca)](https://hiru.mcmaster.ca/hiru/HIRU_Hedges_EMBASE_Strategies.aspx)  Accessed 2022-09-29 and
   Search strategies for MEDLINE in Ovid Syntax, In Health Information Research Unit Hedges project.Ontario:HIRU;2022: [Health Information Research Unit - HIRU ~ Search Strategies for MEDLINE in Ovid Syntax and the PubMed translation (mcmaster.ca)](https://hiru.mcmaster.ca/hiru/HIRU_Hedges_MEDLINE_Strategies.aspx)  Accessed 2022-09-29. [↑](#footnote-ref-2)
2. Search strategies for EMBASE in Ovid Syntax, In Health Information Research Unit Hedges project.Ontario:HIRU;2022: [Health Information Research Unit - HIRU ~ Search Strategies for EMBASE in Ovid Syntax (mcmaster.ca)](https://hiru.mcmaster.ca/hiru/HIRU_Hedges_EMBASE_Strategies.aspx)  Accessed 2022-09-29 and
   Search strategies for MEDLINE in Ovid Syntax, In Health Information Research Unit Hedges project.Ontario:HIRU;2022: [Health Information Research Unit - HIRU ~ Search Strategies for MEDLINE in Ovid Syntax and the PubMed translation (mcmaster.ca)](https://hiru.mcmaster.ca/hiru/HIRU_Hedges_MEDLINE_Strategies.aspx)  Accessed 2022-09-29. [↑](#footnote-ref-3)
3. SR / MA / HTA / ITC - MEDLINE, Embase, PsycInfo. In: CADTH Search Filters Database. Ottawa: CADTH; 2022: <https://searchfilters.cadth.ca/link/33>. Accessed 2022-09-29. [↑](#footnote-ref-4)
4. Geersing GJ, Bouwmeester W, Zuithoff P, et al. Search filters for finding prognostic and diagnostic prediction

   studies in Medline to enhance systematic reviews. *PLoS One* 2012;7(2):e32844.

   <doi:10.1371/journal.pone.0032844> [published Online First: 2012/03/07] [↑](#footnote-ref-5)
5. Wilczynski NL, Haynes RB. Optimal search strategies for detecting clinically sound prognostic studies

   EMBASE: and analytic survey. *J Am Med Inform Assoc* 2005;12(4):481-5. [doi: 10.1197/jamia.M175](doi:%2010.1197/jamia.M175)2 [published

   Online First: 2005/04/02] [↑](#footnote-ref-6)
6. Ingui BJ, Rogers MA. Searching for clinical prediction rules in MEDLINE. *J Am Med Inform Assoc* 2001;8(4):391-7. [doi: 10.1136/jamia.2001.0080391](doi:%2010.1136/jamia.2001.0080391) [published Online First: 2001/06/22] [↑](#footnote-ref-7)
